# Developing a Comprehensive List of Criteria to Evaluate the Characteristics and Quality of eHealth Smartphone Apps: Systematic Review

**DOI:** 10.2196/48625

**Published:** 2024-01-15

**Authors:** Janette Ribaut, Annette DeVito Dabbs, Fabienne Dobbels, Alexandra Teynor, Elisabeth Veronica Mess, Theresa Hoffmann, Sabina De Geest

**Affiliations:** 1 Institute of Nursing Science Department Public Health University of Basel Basel Switzerland; 2 Department of Hematology University Hospital Basel Basel Switzerland; 3 School of Nursing Department of Acute & Tertiary Care University of Pittsburgh Pittsburgh, PA United States; 4 Clinical Translational Science Institute University of Pittsburgh Pittsburgh, PA United States; 5 Academic Center for Nursing and Midwifery Department of Public Health and Primary Care KU Leuven Leuven Belgium; 6 Department of Computer Science University of Applied Sciences Augsburg Germany; 7 Department Pflege und Betreuung Genossenschaft Alterszentrum Kreuzlingen Kreuzlingen Switzerland

**Keywords:** telemedicine, smartphone, mobile apps, program evaluation, decision-making, systematic review, mobile phone

## Abstract

**Background:**

The field of eHealth is growing rapidly and chaotically. Health care professionals need guidance on reviewing and assessing health-related smartphone apps to propose appropriate ones to their patients. However, to date, no framework or evaluation tool fulfills this purpose.

**Objective:**

Before developing a tool to help health care professionals assess and recommend apps to their patients, we aimed to create an overview of published criteria to describe and evaluate health apps.

**Methods:**

We conducted a systematic review to identify existing criteria for eHealth smartphone app evaluation. Relevant databases and trial registers were queried for articles. Articles were included that (1) described tools, guidelines, dimensions, or criteria to evaluate apps, (2) were available in full text, and (3) were written in English, French, German, Italian, Portuguese, or Spanish. We proposed a conceptual framework for app evaluation based on the dimensions reported in the selected articles. This was revised iteratively in discussion rounds with international stakeholders. The conceptual framework was used to synthesize the reported evaluation criteria. The list of criteria was discussed and refined by the research team.

**Results:**

Screening of 1258 articles yielded 128 (10.17%) that met the inclusion criteria. Of these 128 articles, 30 (23.4%) reported the use of self-developed criteria and described their development processes incompletely. Although 43 evaluation instruments were used only once, 6 were used in multiple studies. Most articles (83/128, 64.8%) did not report following theoretical guidelines; those that did noted 37 theoretical frameworks. On the basis of the selected articles, we proposed a conceptual framework to explore 6 app evaluation dimensions: *context, stakeholder involvement*, *features and requirements*, *development processes*, *implementation,* and *evaluation*. After standardizing the definitions, we identified 205 distinct criteria. Through consensus, the research team relabeled 12 of these and added 11 more—mainly related to ethical, legal, and social aspects—resulting in 216 evaluation criteria. No criteria had to be moved between dimensions.

**Conclusions:**

This study provides a comprehensive overview of criteria currently used in clinical practice to describe and evaluate apps. This is necessary as no reviewed criteria sets were inclusive, and none included consistent definitions and terminology. Although the resulting overview is impractical for use in clinical practice in its current form, it confirms the need to craft it into a purpose-built, theory-driven tool. Therefore, in a subsequent step, based on our current criteria set, we plan to construct an app evaluation tool with 2 parts: a short section (including 1-3 questions/dimension) to quickly disqualify clearly unsuitable apps and a longer one to investigate more likely candidates in closer detail. We will use a Delphi consensus-building process and develop a user manual to prepare for this undertaking.

**Trial Registration:**

PROSPERO International Prospective Register of Systematic Reviews CRD42021227064; https://www.crd.york.ac.uk/prospero/display_record.php?ID=CRD42021227064

## Introduction

### Background

eHealth, that is, “the use of information and communication technology for health” [1], can support the delivery of interventions for self-management support and behavior change in patients with acute and chronic illnesses [2,3]. According to the World Health Organization (WHO) [4], self-care health interventions can be classified into individual agency (eg, promoting awareness about self-care), health information seeking (eg, education for informed health decision-making), social and community support (eg, peer mentorship and counseling), personal health tracking (eg, home-based records for health and diagnostic data), self-diagnosis of health conditions (eg, self-testing), self-management of health (eg, self-medication or treatment), individuals’ links to their health systems (eg, individuals sharing data with health care professionals [HCPs]), and individuals’ financial outlays for health (eg, expenses for prescription medicines). However, a recent evaluation of self-care interventions delivered via eHealth apps noted that only 20% of the 100 included apps used evidence-based information, whereas experienced HCPs considered only 32% to be useful and deemed 52% to be misleading and 11% to be dangerous [5].

Searching for a common characteristic linking the most effective apps, several systematic reviews and meta-analyses have found that those developed on firm theoretical foundations are more likely to be effective [6,7]. However, a systematic review of health-promoting smartphone apps found that only 55.6% of the included 27 studies described a theoretical basis for their smartphone app development [8]. A 2018 review found that only 8 (1.2%) of 681 smartphone apps to support medication adherence had documented evidence of their effectiveness. Such evidence is health care systems’ main consideration regarding certification and reimbursement [9]. Furthermore, although a user-centered design (sometimes also called human-centered design) and the involvement of patients and HCPs in the development of smartphone apps is known to provide insights into end users’ needs and helps ensure both relevant, reliable content and high quality [9,10], only 84 (12.3%) of the apps in this review had been developed in collaboration with HCPs. None reported patient involvement in their development processes [9].

Owing to the increasing availability of eHealth smartphone apps, it is vitally important and increasingly challenging for HCPs to identify, evaluate, and recommend relevant, trustworthy, and high-quality eHealth smartphone apps [11-14]. One tempting way for HCPs to form a first idea of an eHealth smartphone app’s quality is the star ratings and written reviews it receives on an app store [15]. However, this information is often subjective and distorted by individuals, comes from unverified or fraudulent sources, or provides no insights on an eHealth app’s quality [15-18]. HCPs also face a lack of reliable guidance on evaluating eHealth smartphone apps’ applicability to clinical practice [13,19,20]. Therefore, many are now struggling to describe and evaluate eHealth smartphone apps. A guideline regarding their characteristics and quality using standardized methods that will allow HCPs to propose reliable apps to their patients is needed [21,22].

Previous efforts to evaluate apps have generally focused on guidance for researchers [23-25]. Although the criteria were often overly complex or tailored to specific health areas, they also tended to be incomplete. Their underlying theories, scientific rationales, and development processes have rarely been described [20,25-27]. Furthermore, their unsuitable foci, nontransparent development processes, complexity, and often excessive time demands make them a poor fit for clinical practice. Finally, the existing instruments used a variety of criteria that only partially overlapped [20,23-27]. A clear description and evaluation of an app is important as, in rapidly evolving fields, even small changes or improvements to an app can have significant impacts on its use and usefulness [28]. To date, evaluation tools to help clinicians describe and evaluate eHealth apps, allowing them to recommend high-quality apps to their patients and share their thoughts using common terminology, are lacking [29,30].

### Objectives

**Figure 1:**
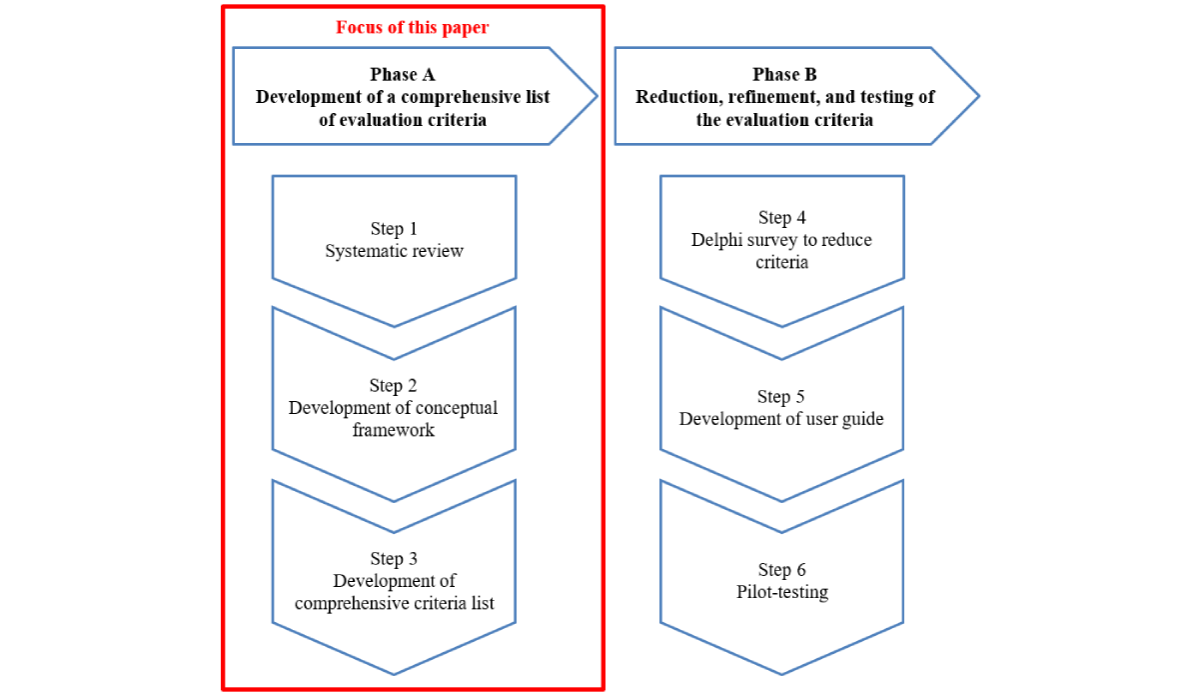
Overview of the 2 phases and steps in the development of the eHealth smartphone app evaluation tool (the focus of this paper is framed on the left side).

Therefore, the aim of this study was to obtain an overview of the evaluation criteria used in the literature. This process was conducted in three steps: (1) conducting a systematic review to identify existing criteria for evaluating eHealth apps, (2) developing a conceptual framework for the evaluation of eHealth smartphone apps, and (3) developing a comprehensive list of criteria for describing and evaluating eHealth smartphone apps. This was the foundational phase 1 of an overarching project to develop and pilot-test a theory-based tool to help HCPs evaluate the characteristics and quality of eHealth smartphone apps in a practical and standardized way (Figure 1). Phase 2 will involve narrowing down, refining, and testing the evaluation criteria via 3 further steps: conducting a Delphi survey to narrow down the criteria (step 4), developing a user guide for the processes of description and evaluation (step 5), and pilot-testing the user guide and processes with HCPs (step 6). This paper focuses on reporting on the foundational phase 1 and outlining the proposed steps for phase 2.

## Methods

### Design

We used a 3-step descriptive, iterative, and developmental approach in phase 1. We first conducted a systematic review, then iteratively developed a new conceptual framework, and finished by compiling a comprehensive list of criteria for the evaluation of eHealth apps. The methodology for each of these steps is described in detail in the following sections. As this study did not deal with human participants or identifiable data, no ethics approval was needed.

### Step 1: Systematic Review

To identify existing criteria for evaluating eHealth apps, we conducted a systematic review complying with the *Cochrane Handbook for Systematic Reviews of Interventions* [31]. The manuscript was written following the PRISMA (Preferred Reporting Items for Systematic Reviews and Meta-Analyses) guidelines [32].

#### Protocol and Registration

Our review was registered in PROSPERO (CRD42021227064 [33]). No other protocol has been published.

#### Eligibility Criteria

Our systematic review included studies on any health condition fulfilling the following inclusion criteria: all had to be primary studies or reviews (1) explicitly describing tools, guidelines, dimensions, instruments, criteria or items, or development processes for tools to evaluate eHealth smartphone apps; (2) clearly describing the evaluation of interventions delivered via eHealth smartphone apps according to predefined (self-developed or existing) criteria; (3) having full-text versions available; and (4) being available in English, French, German, Italian, Portuguese, or Spanish. We incorporated criteria encompassing a broad spectrum of health care areas, including health promotion, prevention, and both physical and mental health. Recognizing the interconnected nature of physical and mental health and the diverse purposes and user groups for which eHealth is used, we integrated a wide array of objectives and stakeholders into our evaluation. Although these areas may exhibit distinct characteristics, it is conceivable that there are fundamental criteria that could be consistently applied in evaluating eHealth apps across different domains. These fundamental criteria may encompass aspects such as user-friendliness, data security, privacy, and usability, forming a shared foundation for evaluation to ensure that essential quality aspects are addressed. In summary, our systematic review encompasses these comprehensive topics to provide a thorough evaluation of eHealth quality criteria, which are applicable to diverse health care needs. Our aim was to encourage consistency and standardization in the evaluation process. Articles were excluded if they (1) described criteria to evaluate interventions delivered via eHealth websites and videos, among other media, but not smartphone apps; and (2) were study protocols, conference abstracts, editorials, or letters to the editor.

#### Information Sources

We queried the MEDLINE (OvidSP), CENTRAL (via Cochrane), CINAHL (EBSCOhost), and Web of Science databases. Supplementary searches were conducted on trial registries (ClinicalTrials.gov and WHO trial registry) and the reference lists of the included papers. The search was conducted on December 5, 2022. No time restrictions were imposed.

#### Search Strategy

We developed our MEDLINE search string based on the terms used in articles on (partially) similar topics [34-41] combined with key Medical Subject Heading and free-text terms (see the search strategy in Multimedia Appendix 1). For the other databases, we adapted the search string accordingly. We combined thematic blocks with various keywords related to *eHealth*, *smartphone*, *application*, *evaluation*, and *tool*. No filters were applied.

#### Selection Process

All identified titles and abstracts were independently screened for relevance by 2 reviewers (JR and TH). The full texts were assessed by the same reviewers using the criteria described previously. The reasons for full-text exclusion were reported. In one case of disagreement, an independent third researcher (SDG) contributed to help reach a consensus.

#### Data Collection Process and Data Items

In total, 2 reviewers (JR and TH) independently extracted the data and cross-checked their results. We extracted information on the author, year, country, research question or study aim, design, operating system, population or specific condition, main intended intervention purpose, name of the tool, and framework or theoretical guidance. The intended purpose of each eHealth app–delivered intervention was categorized according to the WHO classification for self-care health interventions [4]: individual agency, health information seeking, social and community support, personal health tracking, self-diagnosis of health conditions, self-management of health, individuals’ links to their health systems, and individuals’ financial outlays for health. Specific eHealth app quality evaluation dimensions or criteria were extracted and tabulated in a separate table.

#### Study Assessment

The included studies were assessed using the Appraisal of Guidelines for Research and Evaluation–II (AGREE-II) instrument [42], which is widely used to evaluate guideline development processes. As many of the included studies did not a priori intend to develop an evaluation guideline, this instrument might not have been the best option for all studies. However, as we were mainly interested in the justification and development of the dimensions or criteria used in the studies, this instrument provided us with the best support for evaluating these aspects. The AGREE-II instrument consists of 23 items classified into 6 domains (3 items on scope and purpose; 3 on stakeholder involvement; 8 on rigor of development; 3 on clarity of presentation; 4 on applicability; and 2 on editorial independence, ie, whether funding body and competing interests were reported). We concluded our AGREE-II assessment by rating the degree to which each included study described each domain. For this, we used a 4-descriptor scale: *accurately* (all AGREE-II items fulfilled), *partially* (two-thirds of all AGREE-II items fulfilled), *hardly* (one-third of all AGREE-II items fulfilled), and *not at all* (0 AGREE-II items fulfilled).

### Step 2: Development of a Conceptual Framework

The original dimensions of the frameworks reported in the selected articles were listed in a table. Similar descriptions of dimensions were merged. The first draft of the proposed conceptual framework and graphical representations was reviewed and discussed with various stakeholders (researchers, clinicians, designers, and software developers with backgrounds in nursing, medicine, ethics, and informatics). During these discussions, the participants recommended that we distinguish between technical dimensions (eg, design, usability, security, safety, and privacy) and those that focused on content (eg, evidence base and scientific evaluation). It was also recommended that the dimensions be presented as a linear, step-by-step process. During these meetings, the first author (JR) took notes and recorded the proposed changes until consensus was reached on the next version.

The second draft of the framework was discussed with 18 international volunteers (patient representatives, researchers, clinicians, and technology developers) from diverse backgrounds in health care (eg, psychology, nursing, and pharmacy) who were participating in a public webinar on quality evaluation of eHealth technology. As it was a public webinar, only limited data on participant demographics were collected (Table S1 in Multimedia Appendix 2). A survey via AhaSlides (AhaSlides Pte Ltd) to rate the importance and clarity of the dimensions and subgroups (1=not important; 5=very important) and open-ended questions to add missing dimensions or subgroups were used to engage with the participants. In addition, the participants were engaged to add comments orally that the first author (JR) put in writing. The quantitative data were analyzed descriptively (eg, frequency and mean), whereas the qualitative data were analyzed using the mind-mapping technique. The participants found the *technology* dimension too large (ie, covering too many subtopics) and partially unclear. Therefore, it was recommended to split this dimension into technological concerns (eg, technical requirements, security, safety, and privacy) and functional requirements (eg, the user-centeredness and usability of the design). In addition, they understood eHealth evaluation as a cyclical process and recommended presenting the conceptual framework as a continuous circuit as opposed to the initially linear process recommended previously.

Their feedback was used to draft a third version of the framework, which was presented and discussed with 34 researchers, clinicians, and technology developers (Table S2 in Multimedia Appendix 2; only 1 person overlaps with the volunteers from the webinar) mainly with a background in pharmacy who participated in the Next Chapter in Patient Care Conference in April 2022 in Pärnu, Estonia. This time, a survey via Mentimeter (Mentimeter AB) was used to rate the importance and clarity of the dimensions and subgroups (1=not important; 5=very important), and open-ended questions were used to add missing dimensions or subgroups. In addition, the participants were engaged to add comments orally while the first author (JR) took notes. Quantitative data were analyzed descriptively, whereas qualitative data were analyzed using the mind-mapping technique. The Next Chapter in Patient Care participants recommended highlighting the overarching nature of the ethical, legal, and social aspects, which must be considered in all eHealth smartphone app evaluation dimensions. This resulted in a fourth version reflecting the general character of relevant ethical, legal, and social considerations.

Subsequent rounds of discussion and feedback with the research team focused on the scope and relationships between the dimensions. The participants highlighted the legal, ethical, and social aspects to be treated as part of the context. In addition, they agreed that stakeholder involvement should be seen as another overarching aspect that is important in all dimensions of eHealth app evaluation. This discussion resulted in the final version of our new conceptual framework for evaluating eHealth apps: the eHealth Smartphone App Evaluation (eHAPPI) framework.

### Step 3: Development of a Comprehensive Criteria List

The eHAPPI framework was then used to synthesize all the eHealth smartphone app evaluation criteria identified in the selected studies. The redundant criteria were combined. The classification of the criteria according to the eHAPPI dimensions and suggestions for changes and regarding additional or irrelevant criteria were discussed and refined by the research team according to consensus.

## Results

### Step 1: Systematic Review

#### Study Selection

**Figure 2:**
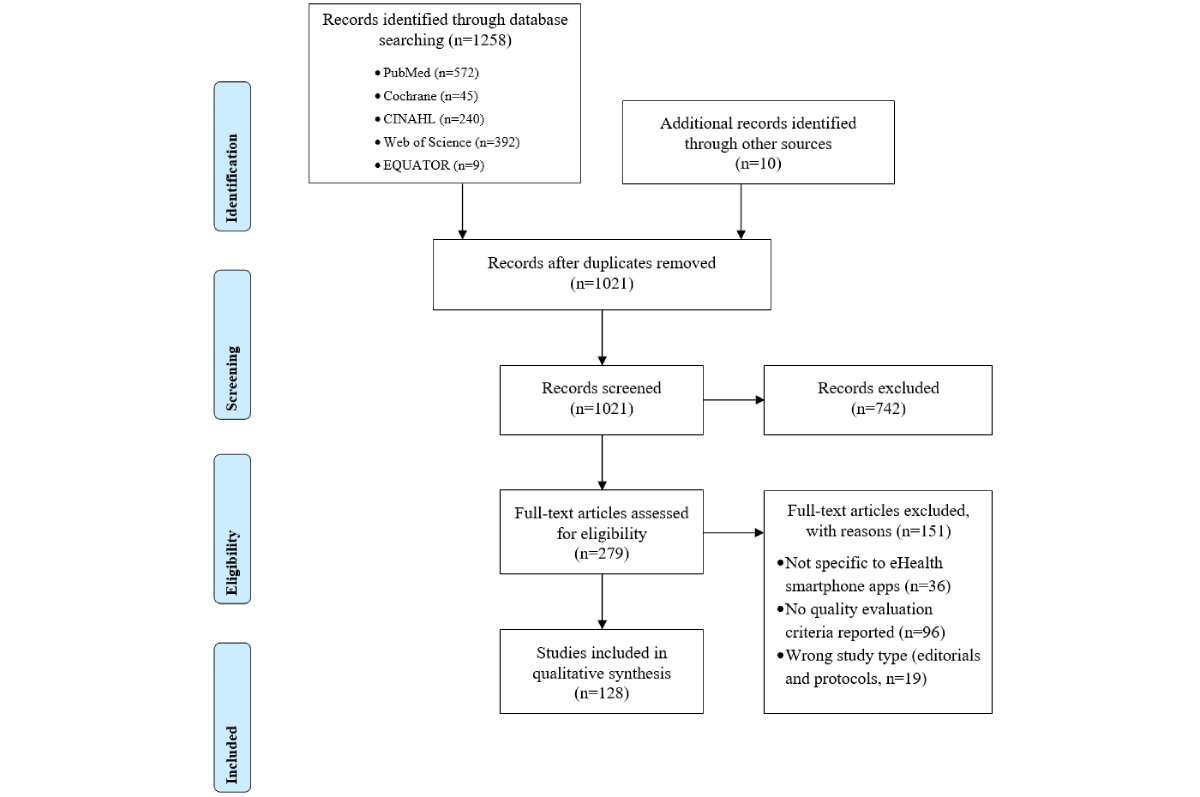
PRISMA (Preferred Reporting Items for Systematic Reviews and Meta-Analyses) flow diagram.

The results of our study selection process are presented in the PRISMA flow diagram (Figure 2). The search strategy described previously yielded 1021 nonduplicate titles. After screening of the titles and abstracts as well as full-text assessment for eligibility, our final analysis included 128 articles that met all the inclusion criteria (Multimedia Appendix 3 [43]).

#### Study Characteristics

##### General Description

A detailed description of the characteristics of the included studies can be found in Multimedia Appendix 4. The years of publication ranged from 2013 to 2022. All but 1.6% (2/128) of the articles were written in English—1 was in French [44] and the other in Spanish [45]. Four-fifths of the studies (101/128, 78.9%) were conducted in Western Europe, North America, or Australia. The studies used a variety of designs, mainly cross-sectional reviews of existing apps (42/128, 32.8%); reports on various app development and evaluation approaches (29/128, 22.7%); quantitative, qualitative cross-sectional, or longitudinal user testing of a single app (27/128, 21.1%); or different forms of reviews (15/128, 11.7%). The 128 included studies involved apps covering 30 topics, such as mental health, health promotion, or support for specific physical conditions (eg, heart disease and diabetes). However, only 50.8% (65/128) of the studies provided enough detail to categorize them according to the WHO classification for self-care interventions for health and well-being [4].

##### Evaluation Tools

In total, the included studies used 142 distinct tools to evaluate eHealth apps. Although 76.6% (98/128) of the included studies used a single evaluation tool, 23.4% (30/128) used multiple tools and scales. Almost one-quarter of the studies (30/128, 23.4%) used an evaluation tool with investigator-developed criteria and then provided only scantily described development processes or theoretical backgrounds for those criteria. The most frequently used tool was the Mobile App Rating Scale (33/128, 25.8%) followed by its adapted versions (8/128, 6.3%) and the System Usability Scale (22/128, 17.2%). Less frequently used tools were the Post-Study System Usability Questionnaire (2/128, 1.6%), the Questionnaire for User Interaction Satisfaction (2/128, 1.6%), and the quality of experience survey (2/128, 1.6%). A total of 43 other tools were used in only 0.8% (1/128) of the studies each. In total, 10.2% (13/128) of the studies used qualitative methods (eg, interviews and focus groups) to generate the app evaluation criteria. In some cases (4/128, 3.1%), the origin of the criteria was unclear, or similar names were used for different tools.

##### Theoretical Frameworks

Most studies (83/128, 64.8%) did not clearly report a theoretical underpinning. The 32% (41/128) that did used 59 different frameworks, including various non–eHealth-specific behavioral, social, or implementation theories (10/59, 17%), the technology acceptance model (7/59, 12%), heuristic evaluation (5/59, 8%), models of the International Organization for Standardization (3/59, 5%), the (extended) Unified Theory of Acceptance and Use of Technology (3/59, 5%), or user-centered design (2/59, 3%). In total, 29 frameworks were used in only 2% (1/59) of the studies each. Of the 59 frameworks used in the included studies, 16 (27%) guided the development and 43 (73%) guided the evaluation of eHealth smartphone apps. In 1.6% (2/128) of the studies, different frameworks were used for development and evaluation.

#### Study Assessment

On average, the studies described 8.6 (SD 2.4; range 3-15) of the AGREE-II instrument’s 23 items [42]. Few studies described the scientific or theoretical basis and development processes of the app evaluation criteria that they applied. The most completely described or justified domains were related to *scope and purpose* (116/128, 90.6% described it accurately; 11/128, 8.6% described it partially; and 1/128, 0.8% hardly described it), *editorial independence* (91/128, 71.1% described it accurately; 30/128, 23.4% described it partially; and 7/128, 5.5% did not describe it at all), and *stakeholder involvement* (13/128, 10.2% described it accurately; 42/128, 32.8% described it partially; 70/128, 54.7% hardly described it; and 3/128, 2.3% did not describe it at all). The least fulfilled domains were *applicability* (1/128, 0.8% described it accurately; 2/128, 1.6% described it partially; 22/128, 17.2% hardly described it; and 103/128, 80.5% did not describe it at all), *rigor of development* (13/128, 10.2% described it partially; 53/128, 41.4% hardly described it; and 62/128, 48.4% did not describe it at all), and *clarity of presentation* (14/128, 10.9% described it accurately; 32/128, 25% described it partially; 52/128, 40.6% hardly described it; and 30/128, 23.4% did not describe it at all).

### Step 2: Development of a Conceptual Framework

The full list of frameworks and original eHealth evaluation dimensions identified in the selected studies can be found in Multimedia Appendix 5 [43,45-47]. Some dimensions were included in only a few frameworks, and no framework included all possible dimensions.

**Figure 3:**
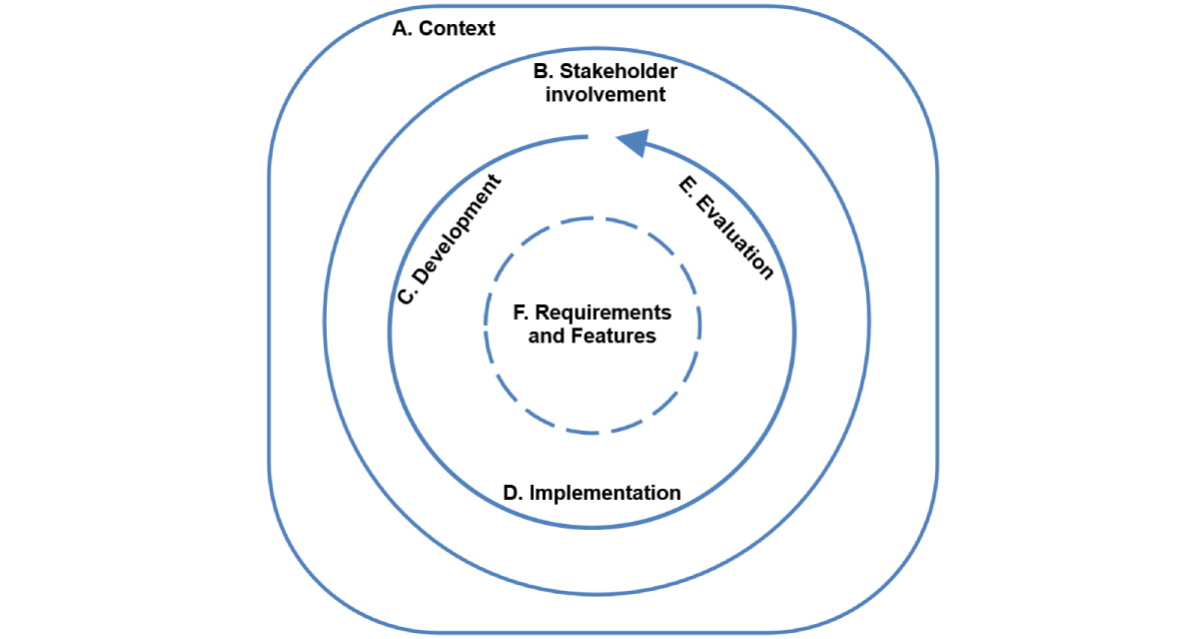
The final eHealth Smartphone App Evaluation framework.

The condensed dimensions were presented graphically and refined iteratively with the stakeholders until consensus was reached and no further adaptions were needed. The final eHAPPI conceptual framework (Figure 3) consists of six interrelated dimensions: (1) *context*, (2) *stakeholder involvement*, (3) *development processes*, (4) *implementation*, (5) *evaluation*, and (6) *features and requirements*.

A detailed definition of each dimension, including the subgroups, is presented in Textbox 1. *Context* describes a set of unique factors and conditions in which the app will be implemented [48]. This is an overarching dimension that depends on and, in turn, influences the other domains. *Stakeholder involvement* is essential in all aspects of eHealth. It involves the active engagement of relevant partners in all processes of the app life cycle, from conceptualization to sustainable implementation (eg, with end users, HCPs, researchers, and health insurers) [49,50].

Several subgroups were defined to further outline and structure the framework: *basic information* (concerning the app) and *ethical, legal*, *and social aspects* were seen as relevant subgroups of the *context* dimension. The *features* *and* *requirements* dimension was assigned 4 subgroups: *evidence-based content*; *functionality*; *usability*, *privacy*, *and security*; and *performance.* Similarly, the *development process* dimension was divided into *cocreation/user-centered design* and *characteristics of the development team*, and *adoption (integration into daily life)* and *maintenance* were seen as relevant subgroups of the *implementation* dimension. Finally, the *evaluation* dimension included only 1 subgroup: *scientific evaluation*.

**Overview of framework dimensions, subgroups, and definitions.**

**Context**

- *Context* describes the set of unique factors and conditions in which the app is implemented. During the implementation process, the app, implementation, and context interact, influencing, modifying, facilitating, or hindering one another. As an overarching concept, context encompasses a physical location and roles, relationships, and interactions at various levels (ethical, legal, social, geographical, epidemiological, and political) [48,51].
- *Basic information* describes general information concerning the app (eg, name, URL, or available languages).
- *Ethical, legal, and social aspects* describe activities to help evaluators consider ethical, legislative, and social implications of an app’s development and implementation. These aspects cannot be completely separated from each other as they overlap somewhat [28].
  *Ethics* describes activities to understand and explore *the moral life*, wherein *morality* includes beliefs, norms of behaviors, principles, and rules that guide individual and institutional behavior. Morality is a widely shared set of norms that result from a certain social consensus. Ethical aspects include the prevailing moral norms and values during the development and implementation of apps. Although moral values are influenced by cultural, sociopolitical, religious, and economic differences, many ethical reflections are shared by all countries and societies. Regarding apps, important ethical topics include *benefit-harm balance, autonomy, respect for persons*, and *justice and equity and legislation* (when using the app, ethical challenges may arise that were not considered in existing legislation and regulations) [28].
  *Legal aspects* include rules and regulations that must be considered when developing and implementing apps. Rules and regulations are designed to protect the rights and interests of the patients and other members of society (eg, legislation on patients’ rights; data protection laws; or the regulations, rights, and obligations of health care professionals [HCPs] in general). Important legal topics are, for example, *autonomy of the patient* (eg, legal requirements), *privacy of the patient* (eg, no use of “unnecessary” patient information), *equality in health care, ethical aspects* (eg, impact on achievement of human rights), *authorization and safety* (eg, laws and rules regarding safety), *ownership and liability*, and *regulation of the market* [28].
  *Social aspects* describe human-centered activities that approach end users and their social networks as reference points in an app’s development and implementation. These include groups of patients or individuals who might require special consideration (eg, vulnerable populations, people living in remote communities, people with learning difficulties, older people, ethnic minority individuals, and immigrants). Patients’, individuals’, and relatives’ perspectives should be considered when developing and implementing apps. Some social groups may be important for a particular app (eg, ethnic minority individuals and individuals with disabilities), which should be specified. Important social topics include *patients’ perspectives* (eg, expectations and wishes), *social group aspects* (accessibility), and *communication aspects* (eg, explanation of treatment choices) [28]

**Stakeholder involvement**

- *Stakeholder involvement* describes the active engagement of relevant partners in all processes of the smartphone app life cycle, from conceptualization to sustainable implementation (eg, end users, HCPs, researchers, and health insurers [49,50].
  Note: for end-user involvement in the development process, see the *Cocreation/user-centered design* section under the *Development process* subheading in this textbox).

**Development process**

- *Development process* describes all activities performed regarding the app’s creation, such as activities related to its aim, target population, guiding principles, applied approaches, stakeholder contributions, required changes for subgroups, or continuing uncertainties [52-54].
- Cocreation of technologies or user-centered design: *cocreation* describes a stepwise process in which partners (eg, patients, caregivers, and HCPs) are actively involved in the strategic design and planning of the app’s development and implementation at every stage of the process [55].
  *User-centered design* is a multidisciplinary design approach using cyclic iteration and evaluation. As it actively involves users to improve the developers’ understanding of their requirements and wishes, this approach is seen as essential regarding product benefits and usability [56].
- *Characteristics of the development team* describes information about the multidisciplinary group responsible for the creation of the working, validated app. Important characteristics of the development team include the name of the app provider (developer and manufacturer of technology and content), contact details, organization attributes, and funding, as well as any conflicts of interest [57].

**Evaluation**

- *Evaluation* describes the assessment of the app’s efficacy, effectiveness, cost-effectiveness, safety, implementation, and impact [58].
- *Scientific evaluation* is the systematic assessment of the app’s efficacy, cost-effectiveness, safety, implementation, and impact through observation, measurement, and experimentation in a scientific study. Such an evaluation is essential to reliably measure an app’s effects and outcomes as a basis for decision-making [58-60].

**Implementation**

- *Implementation* describes the uptake and sustainable integration of evidence-based innovations such as apps into routine use [61].
- *Adoption* (ie, integration into daily life) deals with the app’s uptake (ie, activities focused on using the app in everyday life). This includes the user’s reaction to the app with respect to desired activities and interactions, such as downloads, clicks, and data entries. User engagement, that is, the user’s investment in learning about and participating in the app (eg, time and memory load), is an important prerequisite for the app’s long-term integration into daily life [62-64].
- *Maintenance* covers activities that ensure that an app is sustained in an acceptable and safe condition to perform its specified functions. Maintenance also includes repair as a stand-alone action to restore deteriorated or damaged parts. The activities needed to ensure the required performance of the app during its lifetime include updates, performance evaluations, and the planning and execution of necessary repairs [65].

**Features and requirements**

- *Features and requirements* describe the app and the characteristics or attributes it must provide to meet the users’ needs (ie, its *information content, functionality, usability, security, privacy*, and *performance*). The indications, performance features, and app options may differ for different generations or versions of an app. Important *features and requirements* topics include app description, claimed benefits, care level of use, reference values, or cutoff points used [28, 66]. A clear description of the app is important as, in rapidly evolving fields, even small changes or improvements to an app can have large impacts on its use and benefits. The description should enable those who are not familiar with the app to understand what it does, how it works, and how it can be used..
- *Evidence-based content* describes information, subject matter, and data content made available by the app based on the best available scientific knowledge and clinical expertise [67].
- *Functionality* describes the properties or functional requirements that affect the app’s use (ie, its *features, components*, and *usefulness*). In the context of self-monitoring, there are specific requirements related to design issues, particularly those concerning wearable technologies, as well as requirements regarding the behavioral aspects that are part of the design. These requirements need to be considered when evaluating a product or system for self-monitoring. The *functionality* information is intended to summarize the app’s overall suitability for use in a particular situation [38,68,69].
- *Usability, privacy*, and *security* are nonfunctional requirements (ie, they deal with how the system should operate in terms of usability, security, privacy, design, modularity, modifiability, reliability, availability, portability, and operability).
  *Usability* describes the extent to which an app can be used by its target users to achieve its intended goals *effectively, efficiently,* and *satisfactorily*. Ideally, an app should be *easy to use, easy to learn*, and *easy to understand* [70].
  *Privacy* describes the protection of and control over personal data during the app’s processing operations [71].
  *Security* describes the provision of safeguards that serve the security of the individual or community. For example, this helps prevent or avoid poverty, hardship, theft, or espionage. Security is a major component of a stable, relatively predictable environment in which people can pursue their goals without interference, harm, or the fear of them. This includes protecting the app and associated data from events that could cause loss or severe damage, such as fire, burglary, theft, or vandalism [72,73].
- *Performance* describes whether an app works quickly and without errors and does not cause problems. Important performance topics include reliability and scalability (ie, whether an app still works properly when the number of users increases [74,75]).

### Step 3: Development of a Comprehensive Criteria List

In total, 205 criteria for describing and evaluating eHealth smartphone apps were reported in the selected articles. None of the articles included all the possible criteria. The use of terminology differed within the publications or was attributed different meanings. For example, several were named *usability* but referred to different aspects of that criterion, such as *ease of use*, *usefulness*, or *speed*. Others, such as *research-backed*, *scientific references*, *information accuracy*, and *information quality*, were named differently but clearly referred to a single criterion—in this case, *evidence-based information*.

Using the new eHAPPI framework, it is clear that most studies reported criteria that focused on usability, evidence-based content, functionality, or scientific evaluation. However, only 1 reported criterion dealt with context, and only 5 dealt with development processes.

The research team’s discussions emphasized that some criteria are objective (eg, *average rating in the app store* and *purpose of the app*). In contrast, others are more subjective (eg, *matching the needs of the target population* and *intention to use*). In addition, several were dependent on the tested app’s purpose or content (eg, *whether an app community exists* and *features to support behavior change*).

The research team agreed on how all the criteria were classified into dimensions and how most were formulated. However, to improve understanding or align with known formulations, they suggested revisions to the wording of 12 criteria [28]. On the basis of the research team’s recommendations (Multimedia Appendix 6 [25,45-47,76-85]), 11 new criteria were added (Multimedia Appendix 7 [28]). These additions were mainly to the *ethical, legal, and social aspects* section. No criteria were deemed completely irrelevant, so none were removed. Finally, the research team agreed to classify these added criteria into the existing dimensions.

The final list contained 216 criteria for describing and evaluating eHealth smartphone apps (Multimedia Appendix 8). Although this list was comprehensive, our discussions highlighted that it was not practical for use by HCPs in clinical practice. The research team agreed that, in the next phase, a short version with only 1 to 3 items per dimension would be useful to make a quick initial decision (ie, acting as an algorithm to gauge whether an app should be given further consideration). Only if an app passed this pretest would it undergo a more thorough evaluation using detailed criteria and offering nuanced results. In addition, the research team recommended a specific algorithm with thresholds that could be adjusted depending on each tested app’s purpose and context.

The research team recommended that the next step for the project’s second phase be a Delphi process to condense the list and develop the proposed decision support tool. This process will have two aims: (1) to provide a means to reach a consensus and (2) to develop a useful and feasible (ie, practical for use in clinical practice) tool to describe and evaluate eHealth smartphone apps.

Moreover, participants expressed concerns that the necessary information to complete the tool may be difficult to find. Therefore, the development of a user guide for HCPs on how to apply the tool and where to typically find the required information was also proposed for the next phase. Finally, the eHealth smartphone app evaluation tool and user guide will need to be pilot-tested with HCPs.

## Discussion

### Principal Findings

Although the eHealth field is rapidly expanding and evolving, there is no consensus on how the quality of eHealth apps should be defined and evaluated by HCPs [13,19,20,86]. In this paper, we described how we developed a comprehensive list of criteria to evaluate eHealth apps. We used a meticulous methodological approach to derive the list, consisting of a systematic literature review and iterative rounds with stakeholders from various backgrounds to compose a comprehensive framework—the eHAPPI—and use it to synthesize all the criteria identified in the selected studies. We found 6 overarching dimensions (ie, *context*, *stakeholder involvement*, *features and requirements*, *development processes*, *implementation*, and *evaluation*) of eHealth app evaluation and 205 criteria in the literature. A research team discussion resulted in 11 additional criteria, bringing the new total to 216. Using this comprehensive list, HCPs would be able to evaluate eHealth apps designed for diverse health care needs. No original studies included all the dimensions or all the criteria. Most of the selected studies (83/128, 64.8%) did not describe an underlying framework or theoretical guidance or how their criteria were developed. In addition, a general lack of common terminology among the included studies further complicated efforts toward comparison and direct application in clinical practice.

Although the proposed complete list of criteria was comprehensive, applicable to diverse health care needs, and applied consistent terminology, its length and depth limited the feasibility of using it in clinical practice. As a stand-alone reference, this list would be better suited for comprehensive evaluations of a broad range of eHealth apps by a regulatory body. Therefore, we proposed a 2-step approach to developing these criteria for use by HCPs. The first step would be defining a few critical initial decision criteria. A more detailed description and assessment would be useful only for positive evaluations using these first criteria. Using such an algorithm would allow the most obviously inferior apps to be quickly excluded from the process.

However, our progress in this direction was slowed by a lack of consensus regarding which criteria were essential and which would simply be nice to have [23]. As a compromise, we agreed that developing the proposed algorithm would require more expertise than we had and further expert discussion. Therefore, for the second phase of this project, we proposed a Delphi process [87] to guide the further development and fine-tuning of the final evaluation tool.

Although we did not reach a consensus on which criteria qualify as essential, our group discussions provided insights into which qualities to consider in describing and evaluating apps as well as how an optimal tool might be structured. For example, given the dynamic and rapidly evolving use of apps in clinical practice [29,88], flexibility is a significant concern [23,25]. Therefore, the research team recommended an algorithm not only whose cutoff criteria can be modified to fit each evaluated app’s purpose and context but also whose overall functionalities can evolve alongside the surrounding technology [89].

Another point of discussion concerned the difficulty we encountered in finding the information to complete this evaluation tool. HCPs who are less familiar with eHealth apps may have difficulty gathering even basic data, such as the name of an app’s developer or its latest update [13,21,22]. More technologically savvy individuals may have trouble finding information on that app’s scientific basis, how or whether its development processes included stakeholder involvement, or which strategies were used in its implementation. Therefore, we suggest that the proposed tool include a user guide describing why such criteria are important and where to find and how to judge the required information. This echoes a recommendation by the European Network to Advance Best Practices and Technology on Medication Adherence in their Cooperation in Science and Technology Action (CA19132), which facilitates the use of a web-based repository of information on medication adherence technology [90,91]. Although eHealth app developers clearly need to provide relevant details in a clear and easily accessible way, health educators also need to include eHealth evaluation in HCP education and training curricula.

The previous discussion provides the foundation for conducting phase 2 of this study. This phase has three goals: (1) to conduct a Delphi survey to narrow down the number of criteria and develop an algorithm for initial decision-making, (2) to develop a user guide, and (3) to pilot-test the resulting iteration.

### Limitations

This study has several limitations. Most notably, at this point in the project, although our list of eHealth app description and evaluation criteria is comprehensive, it remains a preliminary version. That is, despite discussions with various interdisciplinary experts, phase 1 did not produce an in-depth consensus on the essential criteria for a richly detailed but broadly feasible means of evaluating eHealth apps. This drove the decision to design a 2-phase project. In phase 2, which will be a Delphi study [87], we aim to develop a criteria-sorting algorithm. With this in place, the phase will culminate in a version of an eHealth app evaluation tool for pilot-testing.

In addition, all the included studies were assessed using the AGREE-II instrument [42], which was specifically designed to assess clinical practice guideline development reports. Considering the high level of heterogeneity across many of the study characteristics, direct comparability using a single tool was limited. However, although other instruments would have been more suitable in many cases, using various instruments would have yielded equally varied results. As we were primarily interested in the rationales and development processes that supported the dimensions and criteria, the AGREE-II scales provided a consistent assessment of these aspects.

Finally, the participants in the discussion rounds for the development of the conceptual framework were primarily health care researchers and professionals. There were very few technology developers or industry representatives present, and only 2 patient representatives participated. This may have resulted in a limited consideration of the patient perspective and an increased risk of interventions exacerbating existing health care inequalities [92]. Therefore, we included in the *context* dimension of the eHAPPI framework subgroups focusing on ethical and social aspects. These subgroups aim to underscore the necessity of addressing the risk of intervention-generated inequalities.

### Comparison With Prior Work

Although 23 of the existing tools were explicitly intended for HCPs [14,43,46,47,76-86,93-100], none of these were complete; rather, they were too focused on specific conditions, or their theoretical justifications or development processes were not described. Such omissions make it difficult for HCPs in clinical practice to comprehensively but feasibly describe and evaluate eHealth apps in a standardized way to guide the recommendation of relevant, reliable, and high-quality apps to their patients [11,13,14]. Other studies supplemented the dimensions and criteria for describing and evaluating apps. However, it remains unclear which criteria are essential and how detailed they need to be. Recently, there has been much discussion about how to define and evaluate eHealth quality and what criteria are needed for an app to be used in the health care system [23,89,101]. Future findings from the planned phase 2 will likely provide a basis for further discussion on this topic among app developers or providers, HCPs, patients, researchers, and policy makers. Our first comprehensive list of criteria as a result of phase 1 provides an excellent basis for the next steps in phase 2 to develop a new eHealth app evaluation tool.

The need for a tool to describe and evaluate eHealth apps and help HCPs and their patients navigate the digital health ecosystem is pressing [23]. Our path to a proposed resolution has been quite complex as this field is also complex. After listing the criteria identified via a literature review, we developed them through expert discussions, revealing important improvement areas. In particular, compared with recommendations from the Health Technology Assessment Core Model [28], the criteria concerning the ethical, legal, and social aspects of eHealth apps were deemed incomplete. Therefore, in addition to adapting many criteria, we added several.

### Contribution of This Study

This study focuses on addressing the rapidly growing and somewhat chaotic field of eHealth, particularly the challenges faced by HCPs when it comes to evaluating and recommending health-related smartphone apps to their patients. This study’s contribution lies in its comprehensive methodology for gathering and categorizing existing criteria for evaluating health apps, which is essential as no single framework or evaluation tool effectively serves this purpose. The methodology involved a systematic review of the literature, which resulted in the identification of 216 distinct evaluation criteria organized within a conceptual framework comprising 6 app evaluation dimensions. These dimensions encompass various aspects, including the app’s context, stakeholder involvement in its development, features, development processes, implementation, and evaluation. This study highlights the need for a more purpose-built, theory-driven tool to help HCPs assess and recommend apps effectively and outlines plans to create a 2-part app evaluation tool based on the gathered criteria, which will expedite the process of disqualifying unsuitable apps and scrutinizing potential candidates more closely. This study serves as a crucial foundational step toward developing a practical tool that can guide HCPs in evaluating and recommending health-related apps.

### Conclusions

Developing a tool comprehensive enough for HCPs to reliably describe and evaluate the full range of eHealth apps yet short enough to be feasible for daily clinical practice is a daunting challenge. After our literature review yielded a list of criteria too bulky for routine use, there was a lack of consensus either on terminology or on relevance to define and evaluate app quality. In this report of phase 1, we provided our initial comprehensive overview of 216 relevant criteria used in the selected studies to describe, evaluate, and recommend eHealth apps. To condense this list to a more manageable size, in phase 2, we will formulate and apply a robust consensus-building process to generate a list of criteria ranked by importance, followed by the creation of an algorithm to produce short- and long-form evaluations to match the characteristics of the apps to be evaluated. In addition, the development of a user guide and pilot-testing of the tool are planned. As a basis for informed guidance and decision-making, such a tool will help HCPs reliably describe and evaluate eHealth apps for their patients.

## Appendix

Multimedia Appendix 1 MEDLINE search strategy.

Multimedia Appendix 2 Demographics of participants.

Multimedia Appendix 3 Included articles.

Multimedia Appendix 4 Characteristics of the included studies.

Multimedia Appendix 5 Original frameworks and dimensions.

Multimedia Appendix 6 Revised criteria.

Multimedia Appendix 7 Additional criteria.

Multimedia Appendix 8 Complete criteria list.

## Abbreviations

AGREE-II: Appraisal of Guidelines for Research and Evaluation–II
eHAPPI: eHealth Smartphone App Evaluation
HCP: health care professional
PRISMA: Preferred Reporting Items for Systematic Reviews and Meta-Analyses
WHO: World Health Organization

## References

[1] Using e-health and information technology to improve health 2020. World Health Organization.

[2] Bevans M, El-Jawahri A, Tierney D, Wiener L, Wood WA, Hoodin F, Kent EE, Jacobsen PB, Lee SJ, Hsieh MM, Denzen EM, Syrjala KL (2017). National institutes of health hematopoietic cell transplantation late effects initiative: the patient-centered outcomes working group report. Biol Blood Marrow Transplant.

[3] Gee PM, Greenwood DA, Paterniti DA, Ward D, Miller LM (2015). The eHealth enhanced chronic care model: a theory derivation approach. J Med Internet Res.

[4] (2021). Classification of self-care interventions for health: a shared language to describe the uses of self-care interventions. World Health Organization.

[5] Mueller SM, Jungo P, Cajacob L, Schwegler S, Itin P, Brandt O (2019). The absence of evidence is evidence of non-sense: cross-sectional study on the quality of psoriasis-related videos on YouTube and their reception by health seekers. J Med Internet Res.

[6] Taylor N, Conner M, Lawton R (2012). The impact of theory on the effectiveness of worksite physical activity interventions: a meta-analysis and meta-regression. Health Psychol Rev.

[7] Dombrowski S, Sniehotta F, Avenell A, Johnston M, MacLennan G, Araújo-Soares V (2012). Identifying active ingredients in complex behavioural interventions for obese adults with obesity-related co-morbidities or additional risk factors for co-morbidities: a systematic review. Health Psychol Rev.

[8] Schoeppe S, Alley S, Van Lippevelde W, Bray NA, Williams SL, Duncan MJ, Vandelanotte C (2016). Efficacy of interventions that use apps to improve diet, physical activity and sedentary behaviour: a systematic review. Int J Behav Nutr Phys Act.

[9] Ahmed I, Ahmad N, Ali S, Ali S, George A, Saleem Danish H, Uppal E, Soo J, Mobasheri MH, King D, Cox B, Darzi A (2018). Medication adherence apps: review and content analysis. JMIR Mhealth Uhealth.

[10] Eckert T, Wunsch K, Fiedler J, Woll A (2022). SMARTMOVE-Involving families in the development and implementation of mHealth interventions. Pravention Und Gesundheitsforderung.

[11] Magnol M, Eleonore B, Claire R, Castagne B, Pugibet M, Lukas C, Tournadre A, Vergne-Salle P, Barnetche T, Truchetet ME, Ruyssen-Witrand A (2021). Use of eHealth by patients with rheumatoid arthritis: observational, cross-sectional, multicenter study. J Med Internet Res.

[12] Taeger J, Müller-Graff FT, Hagen R, Rak K (2021). [Application areas of medical apps in otolaryngology]. HNO.

[13] Cummings E, Borycki E, Roehrer E (2013). Issues and considerations for healthcare consumers using mobile applications. Stud Health Technol Inform.

[14] Knitza J, Simon D, Lambrecht A, Raab C, Tascilar K, Hagen M, Kleyer A, Bayat S, Derungs A, Amft O, Schett G, Hueber AJ (2020). Mobile health usage, preferences, barriers, and eHealth literacy in rheumatology: patient survey study. JMIR Mhealth Uhealth.

[15] Kuehnhausen M, Frost V (2013). Trusting smartphone Apps? To install or not to install, that is the question. Proceedings of the 2013 IEEE International Multi-Disciplinary Conference on Cognitive Methods in Situation Awareness and Decision Support.

[16] Dinour LM, Pole A (2022). Evaluation of breastfeeding app features: content analysis study. JMIR Pediatr Parent.

[17] Chiang M (2012). Networked Life: 20 Questions and Answers.

[18] Girardello A, Michahelles F (2010). AppAware: which mobile applications are hot?. Proceedings of the 12th international conference on Human computer interaction with mobile devices and services.

[19] Stach M, Kraft R, Probst T, Messner E, Terhorst Y, Baumeister H (2020). Mobile health app database - a repository for quality ratings of mHealth apps. Proceedings of the 2020 IEEE 33rd International Symposium on Computer-Based Medical Systems.

[20] Krick T (2021). Evaluation frameworks for digital nursing technologies: analysis, assessment, and guidance. An overview of the literature. BMC Nurs.

[21] Quinn S, Bond R, Nugent C (2017). Quantifying health literacy and eHealth literacy using existing instruments and browser-based software for tracking online health information seeking behavior. Comput Human Behav.

[22] Lam M, Hines M, Lowe R, Nagarajan S, Keep M, Penman M, Power E (2016). Preparedness for eHealth: health sciences students’ knowledge, skills, and confidence. J Inf Technol Educ Res.

[23] Nebeker C, Bartlett Ellis RJ, Torous J (2020). Development of a decision-making checklist tool to support technology selection in digital health research. Transl Behav Med.

[24] Dansky KH, Thompson D, Sanner T (2006). A framework for evaluating eHealth research. Eval Program Plann.

[25] Stoyanov SR, Hides L, Kavanagh DJ, Zelenko O, Tjondronegoro D, Mani M (2015). Mobile app rating scale: a new tool for assessing the quality of health mobile apps. JMIR Mhealth Uhealth.

[26] Anthony Berauk VL, Murugiah MK, Soh YC, Chuan Sheng Y, Wong TW, Ming LC (2018). Mobile health applications for caring of older people: review and comparison. Ther Innov Regul Sci.

[27] Gomis M, Gil D, Lopez L, Brossa V, Mirabet S, Roig E, Mangues M (2016). Impact of mHealth in heart transplant management (mHeart). Int J Integr Care.

[28] (2016). Work package 8. HTA core model® version 3.0. EUnetHTA Joint Action.

[29] Kuziemsky C, Lau F (2016). Handbook of eHealth Evaluation: An Evidence-based Approach.

[30] Simera I, Moher D, Hirst A, Hoey J, Schulz K, Altman D (2010). Transparent and accurate reporting increases reliability, utility, and impact of your research: reporting guidelines and the EQUATOR Network. BMC Med.

[31] Higgins JP, Green SE (2011). Cochrane Handbook for Systematic Reviews of Interventions: Cochrane Book Series. Volume 4.

[32] Page MJ, Moher D, Bossuyt PM, Boutron I, Hoffmann TC, Mulrow CD, Shamseer L, Tetzlaff JM, Akl EA, Brennan SE, Chou R, Glanville J, Grimshaw JM, Hróbjartsson A, Lalu MM, Li T, Loder EW, Mayo-Wilson E, McDonald S, McGuinness LA, Stewart LA, Thomas J, Tricco AC, Welch VA, Whiting P, McKenzie JE (2021). PRISMA 2020 explanation and elaboration: updated guidance and exemplars for reporting systematic reviews. BMJ.

[33] Ribaut J, DeVito Dabbs A, Teynor A, Dobbels F, De Geest S (2021). Development of an evaluation tool to assess and evaluate the characteristics and quality of eHealth applications: a systematic review and consensus finding. National Institute for Health and Care Research.

[34] McKay FH, Cheng C, Wright A, Shill J, Stephens H, Uccellini M (2018). Evaluating mobile phone applications for health behaviour change: a systematic review. J Telemed Telecare.

[35] Vukovic V, Favaretti C, Ricciardi W, de Waure C (2018). Health technology assessment evidence on e-health/m-health technologies: evaluating the transparency and thoroughness. Int J Technol Assess Health Care.

[36] Chi NC, Demiris G (2015). A systematic review of telehealth tools and interventions to support family caregivers. J Telemed Telecare.

[37] Boudreaux ED, Waring ME, Hayes RB, Sadasivam RS, Mullen S, Pagoto S (2014). Evaluating and selecting mobile health apps: strategies for healthcare providers and healthcare organizations. Transl Behav Med.

[38] Eysenbach G, CONSORT-EHEALTH Group (2011). CONSORT-EHEALTH: improving and standardizing evaluation reports of Web-based and mobile health interventions. J Med Internet Res.

[39] Bessell TL, McDonald S, Silagy CA, Anderson JN, Hiller JE, Sansom LN (2002). Do Internet interventions for consumers cause more harm than good? A systematic review. Health Expect.

[40] Barello S, Triberti S, Graffigna G, Libreri C, Serino S, Hibbard J, Riva G (2016). eHealth for patient engagement: a systematic review. Front Psychol.

[41] Eland-de Kok P, van Os-Medendorp H, Vergouwe-Meijer A, Bruijnzeel-Koomen C, Ros W (2011). A systematic review of the effects of e-health on chronically ill patients. J Clin Nurs.

[42] (2019). Appraisal of guidelines for research and evaluation (AGREE) II instrument. The AGREE Next Steps Consortium.

[43] Guan Z, Sun L, Xiao Q, Wang Y (2019). Constructing an assessment framework for the quality of asthma smartphone applications. BMC Med Inform Decis Mak.

[44] East-Richard C, Laplante L, Vézina J, Cellard C (2018). L’évaluation psychologique par le biais des applications mobiles. Can Psychol.

[45] Grau I, Kostov B, Gallego JA, Grajales Iii F, Fernández-Luque L, Sisó-Almirall A (2016). Método de valoración de aplicaciones móviles de salud en español: el índice iSYScore. Semergen.

[46] Chan S, Torous J, Hinton L, Yellowlees P (2015). Towards a framework for evaluating mobile mental health apps. Telemed J E Health.

[47] Wyatt JC, Thimbleby H, Rastall P, Hoogewerf J, Wooldridge D, Williams J (2015). What makes a good clinical app? Introducing the RCP Health Informatics Unit checklist. Clin Med (Lond).

[48] Pfadenhauer LM, Gerhardus A, Mozygemba K, Lysdahl KB, Booth A, Hofmann B, Wahlster P, Polus S, Burns J, Brereton L, Rehfuess E (2017). Making sense of complexity in context and implementation: the Context and Implementation of Complex Interventions (CICI) framework. Implement Sci.

[49] Pollock A, Campbell P, Struthers C, Synnot A, Nunn J, Hill S, Goodare H, Watts C, Morley R (2017). Stakeholder involvement in systematic reviews: a protocol for a systematic review of methods, outcomes and effects. Res Involv Engagem.

[50] Brugha R, Varvasovszky Z (2000). Stakeholder analysis: a review. Health Policy Plan.

[51] Leppla L, Mielke J, Kunze M, Mauthner O, Teynor A, Valenta S, Vanhoof J, Dobbels F, Berben L, Zeiser R, Engelhardt M, De Geest S, SMILe study team (2020). Clinicians and patients perspectives on follow-up care and eHealth support after allogeneic hematopoietic stem cell transplantation: a mixed-methods contextual analysis as part of the SMILe study. Eur J Oncol Nurs.

[52] Craig P, Dieppe P, Macintyre S, Michie S, Nazareth I, Petticrew M, Medical Research Council Guidance (2008). Developing and evaluating complex interventions: the New Medical Research Council Guidance. BMJ.

[53] Process development definition. Law Insider.

[54] Duncan E, O'Cathain A, Rousseau N, Croot L, Sworn K, Turner KM, Yardley L, Hoddinott P (2020). Guidance for reporting intervention development studies in health research (GUIDED): an evidence-based consensus study. BMJ Open.

[55] Brandsen T, Honingh M, Brandsen T, Verschuere B, Steen T (2018). Definitions of co-production and co-creation. Co-Production and Co-Creation: Engaging Citizens in Public Services.

[56] Mao J, Vredenburg K, Smith PW, Carey T (2005). The state of user-centered design practice. Commun ACM.

[57] Development team. Agile Innovative Solutions.

[58] (1989). PubMed MeSH term: program evaluation. National Center for Biotechnology Information.

[59] Usmani AM, Meo SA (2011). Evaluation of science. Sudan J Paediatr.

[60] PubMed MeSH term: science. National Center for Biotechnology Information.

[61] Eccles MP, Mittman BS (2006). Welcome to implementation science. Implement Sci.

[62] Proctor E, Silmere H, Raghavan R, Hovmand P, Aarons G, Bunger A, Griffey R, Hensley M (2011). Outcomes for implementation research: conceptual distinctions, measurement challenges, and research agenda. Adm Policy Ment Health.

[63] Vande Moere A, Tomitsch M, Wimmer C, Christoph B, Grechenig T (2012). Evaluating the effect of style in information visualization. IEEE Trans Vis Comput Graph.

[64] Boy J, Detienne F (2015). Storytelling in information visualizations: does it engage users to explore data?. Proceedings of the 33rd Annual ACM Conference on Human Factors in Computing Systems.

[65] (2014). Maintenance and repair of concrete structures ? Part 1: general princi-ples (ISO standard no. 16311-1:2014). International Organization for Standardization.

[66] Requirement 2022. Wikipedia.

[67] (2009). PubMed MeSH term: evidence-based practice. National Center for Biotechnology Information.

[68] Cheu LR, Casals A, Cuxart A, Parra A (2005). Towards the definition of a functionality index for the quantitative evaluation of hand-prosthesis. Proceedings of the 2005 IEEE/RSJ International Conference on Intelligent Robots and Systems.

[69] Martinez WH, Wilcke H (1979). The importance of functionality of vegetable protein in foods. Soy Protein and Human Nutrition.

[70] (2018). Ergonomics of human-system interaction: part 11: usability (ISO standard no. 9241-11:2018). International Organization for Standardization.

[71] (2021). Privacy protection: Privacy guidelines for smart cities (ISO standard no. 27570:2021). International Organization for Standardization.

[72] Brooks DJ (2010). What is security: definition through knowledge categorization. Secur J.

[73] Cobb M Physical security. Physical Security TechTarget.

[74] Lo O, Fan L, Buchanan W, Thuemmler C (2013). Conducting performance evaluation of an e-health platform. Information Systems and Technology for Organizations in a Networked Society.

[75] Martínez-Pérez B, de la Torre-Díez I, López-Coronado M (2015). Experiences and results of applying tools for assessing the quality of a mHealth app named heartkeeper. J Med Syst.

[76] Jain YS, Garg A, Jhamb D, Jain P, Karar A (2019). Preparing India to leverage power of mobile technology: development of a bilingual mobile health tool for heart patients. Cardiovasc Hematol Agents Med Chem.

[77] Collins R (2019). Nurses’ perceived usefulness of secure texting applications for the purpose of patient car. Online J Nurs Inform.

[78] Groen G, Jörns-Presentati A, Dessauvagie A, Seedat S, van den Heuvel LL, Suliman S, Grobler G, Jansen R, Mwape L, Mukwato P, Chapima F, Korhonen J, Stein DJ, Jonker D, Mudenda J, Turunen T, Valtiņš K, Beinaroviča A, Grada L, Lahti M (2022). Development of a mobile application for detection of adolescent mental health problems and feasibility assessment with primary health care workers. Issues Ment Health Nurs.

[79] Sanatkar S, Counson I, Mackinnon A, Bartholomew A, Glozier N, Harvey S (2022). Preliminary investigation of shift, a novel smartphone app to support junior doctors' mental health and well-being: examination of symptom progression, usability, and acceptability after 1 month of use. J Med Internet Res.

[80] Kaliyadan F, Ashique K (2020). Use of mobile applications in dermatology. Indian J Dermatol.

[81] Kwan V, Hagen G, Noel M, Dobson K, Yeates K (2017). Healthcare at your fingertips: the professional ethics of smartphone health-monitoring applications. Ethics Behav.

[82] Olfert MD, Barr ML, Hagedorn RL, Long DM, Haggerty TS, Weimer M, Golden J, Maurer MA, Cochran JD, Hendershot T, Whanger SL, Mason JD, Hodder SL (2019). Feasibility of a mHealth approach to nutrition counseling in an Appalachian state. J Pers Med.

[83] Sudol NT, Adams-Piper E, Perry R, Lane F, Chen KT (2019). In search of mobile applications for patients with pelvic floor disorders. Female Pelvic Med Reconstr Surg.

[84] Vasiloglou MF, Christodoulidis S, Reber E, Stathopoulou T, Lu Y, Stanga Z, Mougiakakou S (2020). What healthcare professionals think of "nutrition and diet" apps: an international survey. Nutrients.

[85] Yasini M, Marchand G (2015). Mobile health applications, in the absence of an authentic regulation, does the usability score correlate with a better medical reliability?. Stud Health Technol Inform.

[86] Johnson E, Emani VK, Ren J (2016). Breadth of coverage, ease of use, and quality of mobile point-of-care tool information summaries: an evaluation. JMIR Mhealth Uhealth.

[87] Fitch K, Bernstein SJ, Aguilar M, Burnand B, LaCalle JR, Lazaro P, van het Loo M, McDonnell J, Vader J, Kahan JP (2001). The RAND/UCLA Appropriateness Method User's Manual.

[88] Rooij T, Marsh S (2016). eHealth: past and future perspectives. Per Med.

[89] (2021). Digital health trends 2021: innovation, evidence, regulation, and adoption. IQVIA Inc.

[90] van Boven JF, Tsiligianni I, Potočnjak I, Mihajlović J, Dima AL, Nabergoj Makovec U, Ágh T, Kardas P, Ghiciuc CM, Petrova G, Bitterman N, Kamberi F, Culig J, Wettermark B (2021). European network to advance best practices and technology on medication adherence: mission statement. Front Pharmacol.

[91] Nabergoj Makovec U, Goetzinger C, Ribaut J, Barnestein-Fonseca P, Haupenthal F, Herdeiro M, Grant SP, Jácome C, Roque F, Smits D, Tadic I, Dima AL, European Network to Advance Best practices and technoLogy on medication adherencE (ENABLE), European Network to Advance Best Practices and TechnoLogy on Medication AdherencE (ENABLE) (2022). Developing a medication adherence technologies repository: proposed structure and protocol for an online real-time Delphi study. BMJ Open.

[92] Veinot T, Mitchell H, Ancker J (2018). Good intentions are not enough: how informatics interventions can worsen inequality. J Am Med Inform Assoc.

[93] Ehrler F, Weinhold T, Joe J, Lovis C, Blondon K (2018). A mobile app (BEDSide mobility) to support nurses' tasks at the patient's bedside: usability study. JMIR Mhealth Uhealth.

[94] Shalan A, Abdulrahman A, Habli I, Tew G, Thompson A (2018). YORwalK: desiging a smartphone exercise application for people with intermittent claudication. Stud Health Technol Inform.

[95] Chang O, Patel VL, Iyengar S, May W (2021). Impact of a mobile-based (mHealth) tool to support community health nurses in early identification of depression and suicide risk in Pacific Island Countries. Australas Psychiatry.

[96] Chirambo GB, Muula AS, Thompson M, Hardy VE, Heavin C, Connor YO, Mastellos N, Andersson B, Donoghue JO (2021). End-user perspectives of two mHealth decision support tools: electronic community case management in Northern Malawi. Int J Med Inform.

[97] Fijačko N, Masterson Creber R, Gosak L, Kocbek P, Cilar L, Creber P, Štiglic G (2021). A review of mortality risk prediction models in smartphone applications. J Med Syst.

[98] Jiang S, Lv M, Wu T, Chen W, Zhang J (2022). A smartphone application for remote adjustment of warfarin dose: development and usability study. Appl Nurs Res.

[99] Shen Z, Zhang Y, Yang C, Liu J, Huang C, Zhang X, Zhang Y, Lin Y (2022). A smart-phone app for fluid balance monitoring in patients with heart failure: a usability study. Patient Prefer Adherence.

[100] Kettlewell J, Phillips J, Radford K, dasNair R (2018). Informing evaluation of a smartphone application for people with acquired brain injury: a stakeholder engagement study. BMC Med Inform Decis Mak.

[101] Kyhlstedt M (2022). The need for action by evaluators and decision makers in Europe to ensure safe use of medical software. Front Med Technol.

