# Supplementary material for: Developing a Comprehensive List of Criteria to Evaluate the Characteristics and Quality of eHealth Smartphone Apps: Systematic Review
Source: JMIR Mhealth Uhealth. 2024 Jan 15;12:e48625. doi: 10.2196/48625 (PMC10825776; doi:10.2196/48625)
Supplement: Multimedia Appendix 1 [file mhealth_v12i1e48625_app1.pdf]

This is a Multimedia Appendix to a full manuscript published in the J Med Internet Res. For full copyright and citation information see http://dx.doi.org/10.2196/48625

MEDLINE search strategy.

Based on the terms used in other articles on (partially) similar topics (1-8), we developed the search strategy for Ovid MEDLINE. It was adapted for Cochrane, CINAHL, Embase and Web of Science using the Systematic Review Accelerator (9). The search strategy consists of each a block with nested brackets on “eHealth”, “Smartphone”, “Application”, “Evaluation” and “Tool”. No filters were applied.

(("Tele-monitoring"[Title/Abstract] OR "Telemonitoring"[Title/Abstract] OR "Tele-healthcare"[Title/Abstract] OR "Telehealthcare"[Title/Abstract] OR "Tele-care"[Title/Abstract] OR "tele-health"[Title/Abstract] OR "tele-medicine"[Title/Abstract] OR "Telecare"[Title/Abstract] OR "Interactive health"[Title/Abstract] OR "mobile Health"[Title/Abstract] OR "m-Health"[Title/Abstract] OR "mHealth"[Title/Abstract] OR "electronic health"[Title/Abstract] OR "e-Health"[Title/Abstract] OR "eHealth"[Title/Abstract] OR "digital medicine"[Title/Abstract] OR "digital health"[Title/Abstract] OR "Telehealth"[Title/Abstract] OR "Telemedicine"[Title/Abstract] OR “Telemedicine”[MeSH Terms])

AND

(“Computers, Handheld”[MeSH] OR "Handheld Computers"[tiab] OR "Palmtop Computer"[tiab] OR "Palmtop Computers"[tiab] OR "Palm-Top Computer"[tiab] OR "Palm-Top Computers"[tiab] OR “Personal Digital Assistant”[tiab] OR "PDA Computer"[tiab] OR "Pocket PC"[tiab] OR "Pocket PCs"[tiab] OR "Tablet Computer"[tiab] OR "Tablet Computers"[tiab] OR "Palm Pilot"[tiab] OR "Palm Pilots"[tiab] OR "Smart Phones"[Title/Abstract] OR "Smart Phone"[Title/Abstract] OR "Smartphones"[Title/Abstract])

AND

("Mobile Applications"[MeSH Terms] OR "Mobile app*"[Title/Abstract] OR "Portable electronic app*"[Title/Abstract] OR "Portable software app*"[Title/Abstract]))

AND

(("Program Evaluation"[MeSH Terms] OR "Evaluation"[Title/Abstract] OR "Program Appropriateness"[Title/Abstract] OR "health technology assessment"[Title/Abstract])

OR

("Practice Guidelines as Topic"[MeSH Terms] OR "Best Practice"[Title/Abstract] OR "Best Practices"[Title/Abstract] OR "Guidelines"[Title/Abstract] OR "Guideline"[Title/Abstract] OR "Guide"[Title/Abstract] OR "Checklist"[MeSH Terms] OR "Checklist"[Title/Abstract] OR "Checklists"[Title/Abstract] OR "Tool"[Title/Abstract] OR "Tools"[Title/Abstract]))
