# Supplementary material for: Developing a Comprehensive List of Criteria to Evaluate the Characteristics and Quality of eHealth Smartphone Apps: Systematic Review
Source: JMIR Mhealth Uhealth. 2024 Jan 15;12:e48625. doi: 10.2196/48625 (PMC10825776; doi:10.2196/48625)
Supplement: Multimedia Appendix 2 [file mhealth_v12i1e48625_app2.pdf]

This is a Multimedia Appendix to a full manuscript published in the J Med Internet Res. For full copyright and citation information see http://dx.doi.org/10.2196/48625

Demographics of participants in the development of the conceptual framework

Table A1. Demographics of volunteers of the public online webinar (n=18).

		N (%)
**Role / profession**		
	Patient representative	2 (11.1%)
	Researcher	8 (44.4%)
	Clinician	3 (16.7%)
	Technology developer	3 (16.7%)
	Missing	2 (11.1%)
**Background**		
	Nursing	2 (11.1%)
	Pharmacy	5 (27.8%)
	Psychology	2 (11.1%)
	Biology	1 (5.6%)
	Medical Sciences	1 (5.6%)
	Informatics, engineering	3 (16.7%)
	NA (patient representative)	2 (11.1%)
	Missing	2 (11.1%)
**Continent**		
	Europe	13 (72.2%)
	Asia	1 (5.6%)
	North America	1 (5.6%)
	Australia	1 (5.6%)
	Missing	2 (11.1%)

Table A2. Demographics of NCPC participants (n=34).

		N (%)
**Role / profession**		
	Researcher	19 (55.9%)
	Clinician	8 (23.5%)
	Technology developer	3 (8.8%)
	Industry	3 (8.8%)
	Missing	1 (2.9%)
**Background**		
	Nursing	2 (5.9%)
	Pharmacy	24 (70.6%)
	Psychology	1 (2.9%)
	Medical Sciences	1 (2.9%)
	Informatics, engineering	3 (8.8%)
	Communication, policy, international affairs	3 (8.8%)
**Education**		
	PhD	8 (23.5%)
	Master	11 (32.4%)
	Master student	12 (35.3%)
	Bachelor	3 (8.8%)
**Continent**		
	Europe	30 (88.2%)
	Asia	1 (2.9%)
	Australia	1 (2.9%)
	Missing	2 (5.9%)
