# Supplementary material for: Developing a Comprehensive List of Criteria to Evaluate the Characteristics and Quality of eHealth Smartphone Apps: Systematic Review
Source: JMIR Mhealth Uhealth. 2024 Jan 15;12:e48625. doi: 10.2196/48625 (PMC10825776; doi:10.2196/48625)
Supplement: Multimedia Appendix 3 [file mhealth_v12i1e48625_app3.pdf]

This is a Multimedia Appendix to a full manuscript published in the J Med Internet Res. For full copyright and citation information see http://dx.doi.org/10.2196/48625

Included articles.

Table A3. Summary of included articles (n=128) [a]

Author, year & country	Research question / study aim	Design	Operating system	Population / condition	Main intervention purpose	Tool	Tool focus	Target user of tool	Framework / theoret. guidance	Framework focus
Agnew et al. 2022 UK	To evaluate the quality of apps related to shoulder pain.	Observational cross-sectional study to review available apps	Android & iOS	Shoulder pain	NA	Mobile Application Rating Scale (MARS)	Quality classification & rating	Researcher	NR	NA
Ahmed et al. 2020 Canada	To examine consumers' perceptions of different versions of an app with nutrition information.	Randomized controlled trial	Android & iOS	Nutrition	Health information seeking	Self-developed criteria	Usability and functionality	User	Multi-dimensional framework for assessing health app quality [1]	Evaluation
Al Ayubi et al. 2014 USA	To review characteristics of mHealth for physical activity promotion, to develop an mHealth app that meets such characteristics, and to conduct a feasibility study of the app.	App development and evaluation	Android	Physical Activity	Health tracking; Social and community support	Fundamental Technical Characteristics; Five Usability Factors	Usability and feasibility	Developer, researcher & user	Behavior change systems; Heuristic evaluation [2], Technology Acceptance Model (TAM) [3]; Unified Theory of Acceptance and Use of Technology (UTAUT) [4]	Behavior change systems: Development Heuristic evaluation, TAM, UTAUT: Evaluation
Alhuwail et al. 2020 Kuwait	To systematically assess depression and anxiety apps available for Arabic speakers.	Cross-sectional review of existing apps	Android & iOS	Depression & Anxiety	NA	MARS	Quality classification & rating	Researcher	NR	NA
Alnasser et al. 2018 Saudi Arabia	To identify the relationship between adherence to evidence-informed practices, user expectations, and app user experiences.	App development and evaluation	Android & iOS	Weight loss	Health tracking	System Usability Scale (SUS); Qualitative word clouds	Usability	User	NR	NA
Amawi et al. 2022 Jordan	To assess the convenience, quality, safety and efficacy of apps for cancer patient.	Observational cross-sectional study to review available apps	Android & iOS	Cancer care	NA	MARS	Quality classification & rating	Researcher	NR	NA
Ambrosini et al. 2018 Australia	To test the acceptability and relative validity of a dietary assessment app.	Longitudinal user test of an app	iOS	Dietary assessment	Health tracking	Self-developed criteria	Acceptability and usability	User	NR	NA

		(quantitative survey)								
Anderson et al. 2016 Australia	To identify relevant apps for academic evaluation, synthesize a checklist for app quality and reliability, and propose a method for evaluating app usability.	Systematic checklist development	Android & iOS	Chronic Diseases	NA	App Chronic Disease Checklist; MARS	Engagement, functionality, ease of use, information management; Quality rating & classification	Researcher	Heuristic evaluation [2]; TAM [3]	Development
Armenta et al. 2019 Mexico & USA	To understand the interaction of the users with the ActEarly mobile app to improve its usability.	Cross-sectional user test of an app (mixed-method)	Android	Children milestone tracking	Health information seeking; Health tracking; Link to health system	Short version SUS; Qualitative think aloud	Usability	User	NR	NA
Báez Gutiérrez et al. 2022 Spain	To identify and assess the quality of apps for patients diagnosed with hematological malignancies	Observational cross-sectional study to review available apps	Android & iOS	Hematologic malignancy	NA	MARS	Quality classification & rating	Researcher	NR	NA
Bauer et al. 2018 USA	To describe the design, development, and deployment of a mobile system to support Collaborative Care.	App development and evaluation	Android	Posttraumat. stress or bipolar disorder	Health information seeking; Health tracking; Link to health system	SUS; Qualitative open-ended questions	Usability; Usability and functionality	User & Researcher	Principles for digital development [5]	Development
Ben-Mussa et al. 2018 UK	To evaluate the most popular medical apps in the UK for compliance with the Health On the Net (HON) Foundation principles.	Cross-sectional review of existing apps	Android & iOS	NA	NA	Modified HON Foundation principles	Information quality	User	HON based on widely accepted ethical principles [6]	Evaluation
Bennion et al. 2019 UK	To evaluate apps that were identified as being used or recommended by the National Health Service.	Cross-sectional review of existing apps	NA	Mental Health	NA	Self-developed criteria	Clinician &, academic involvement, evidence, psychologic approach	Developers, National Health Service	NR	NA
Bentvelsen et al. 2021 Netherlands	To obtain an overview and evaluate available apps for prevention of health care-associated infections.	Scoping review	Android & iOS	Prevention of health care-associated infections	NA	MARS; Functionality categories by Institute for Healthcare Informatics	Quality classification & rating; Functionality	Researcher	NR	NA

Bining et al. 2022 Canada	To evaluate the quality, usefulness, therapeutic potential, and security of publicly available apps to support unpaid cancer caregivers.	Observational cross-sectional study to review available apps	Android & iOS	Cancer caregiver	NA	MARS	Quality classification & rating	Researcher	NR	NA
Birkhoff et al.2018 USA	To examine the usability and acceptability of a health tracking app.	Longitudinal user test of an app (mixed-method)	Android & iOS	Oncology patients on radiation treatments	Health tracking; Link to health system	Quantitative survey; Qualitative open-ended questions	Usability and usability	User	TAM [3]	Evaluation
Biviji et al. 2021 USA	To assess user experience, perceived benefits, and general feedback on maternal and infant health apps by analyzing publicly available user reviews.	Observational cross-sectional study to review available apps	Android & iOS	Maternal and infant health	Health information-seeking; Self-management	Self-developed questionnaire	Content analysis	Researcher	NR	NA
Bosse et al. 2022 USA	To evaluate the perceived usability of existing and planned features of an app to facilitate opioid use disorder treatment.	Cross-sectional user test of an app (mixed-method)	NA	Opioid use disorder treatment	Link to health system	Self-developed questionnaire	Usefulness, usability, functionality	User	User Centered Design [7]	Development
Boulos et al. 2014 USA & UK	To examine the state of the art in mobile clinical and health-related apps.	Scoping Review	Android, Blackberry OS, iOS & Windows	NA	NA	Happtique Health App Certification Standards	Information quality, usability, performance, security, privacy	Certification company	NR	NA
Caballero et al. 2021 Spain	To evaluate an app that extracts contextual information from users, returns it to the server, and augments the information in the app.	App development and evaluation	Android	Pollen allergy	Health information-seeking; Health tracking; Self-management	SUS	Usability	User & researcher	NR	NA
Chan et al. 2015 USA	To propose criteria to assess smartphone, wearable devices and smartwatch apps for mental health.	Checklist development, not clearly reported	Android, Blackberry OS, iOS & Windows	Mental Health	NA	Self-developed criteria	Usefulness, usability, infrastructure	User and health care professional (HCP)	Usefulness, usability, and infrastructure of software dimensions	Evaluation
Chang et al. 2020 Fiji	To transform screening tools for depression and suicide risk into app decision support for community nurses, and to evaluate efficiency,	App development and evaluation	NA	Mental Health	Self-diagnosis of health conditions	SUS	Usability	HCP	Center for Epidemiological Studies Depression Scale (CES-D) [8]; Suicide Behaviors Questionnaire-Revised (SBQ-R) [9]	Development

	effectiveness, and usability.									
Chirambo et al. 2021 Malawi	To explore end users perspectives of two already implemented decision support tools for community case management in Northern Malawi.	Mixed-methods study (survey and individual interviews)	Android	Access to health care services in marginalized communities	Health information-seeking; Self-diagnosis of health conditions	MARS	Quality classification & rating	HCP	(MARS)	Evaluation
Choi et al. 2018 USA	To identify and assess the functionalities of available apps to support sleep self-management.	Cross-sectional review of existing apps	Android, iOS & Windows	Sleep	NA	MARS; IMS Institute for Healthcare Informatics functionality scores	Quality classification & rating; Functionality	Researcher	NR	NA
Collins 2019 USA	To explore nurses' willingness to adopt secure apps and their perception of ease of use.	Cross-sectional survey	NA	NA	NA	Intention to Use survey	Intention to use	HCP	TAM [3]	Evaluation
Cruz Zapata et al. 2018 Spain & Morocco	To propose a software requirement catalogue for the development or evaluation of mHealth apps.	Catalogue development by SIREN (SImple REuse of software requiremeNts) methodology	NA	NA	NA	Usability on mobile health applications-specifications	Usability	Developer and auditor	ISO/IEC/IEEE 29148:2011 [10]; SIREN [11]	SIREN: Development ISO: Evaluation
Davalbhakta et al. 2020 India	To provide an overview of mobile apps around COVID-19 and an assessment of their functions and quality.	Systematic Review	NA	COVID-19	NA	MARS	Quality classification & rating	Researcher	NR	NA
Davis et al. 2017 USA	To critique existing apps using criteria and health literacy guidelines.	Cross-sectional review of existing apps	iOS	Parenting	NA	Patient Education Materials Assessment Tool	Information quality	Researcher	NR	NA
Dubov et al. 2021 USA	To test feasibility, usability, user engagement and satisfaction of an evidence-informed suicide prevention app.	Qualitative interviews and focus groups	NA	Suicide prevention among transgender persons	Health tracking; Self-management	Interview guide adapted from SUS and Standardized User Experience Percentile Rank Questionnaire	Usability; User experience	User	Meyer Minority Stress framework [12]	Development
Dunn Lopez et al. 2021 USA	To determine readability, types of functions, and linkage to authoritative sources of evidence for self-care	Observational cross-sectional study to review available apps	Android & iOS	Heart failure patients	NA	Self-developed questionnaire	Readability, functionality, evidence base	Researcher	TAM	Evaluation

	focused apps for heart failure patients.									
East-Richard et al. 2018 Canada	To assess evaluative mobile apps in psychology and neuropsychology.	Cross-sectional review of existing apps	Android, iOS & Windows	Psychological or neuro-psychological assessment	NA	Adapted Ordre des conseillers/ères d'orientation et des psychoédu-cateurs/rices du Québec (OCCOPPQ) questionnaire	Usability, accuracy, information quality	Researcher	Testing standards of practice (OCCOPPQ) and standards for mobile device interfaces [13]; Operating system design guidelines [14-16]	Evaluation
Egan et al. 2022 UK	To test feasibility, acceptability and usability of a digital health app for caregivers to improve physical activity.	Longitudinal user test of an app (mixed-method)	Android	Physical activity in caregivers	Health information-seeking; Self-management	Self-developed questionnaire	Feasibility, usability, acceptability	User	Transtheoretical model of behavior change [17]; extended UTAUT (UTAUT2) [18]	Evaluation
Ehrler et al. 2018 Switzerland	To assess the usability of the BEDSide Mobility app to support nursing workflow.	Cross-sectional user test of an app (mixed-method)	Android	NA	Health tracking; Link to health system	SUS; Qualitative think aloud & eye-tracking	Usability	HCP	NR	NA
Eisner et al. 2019 UK	To develop an app for monitoring early signs, basic and psychotic symptoms; and qualitatively evaluate its long-term acceptability.	App development and evaluation	NA	Psychosis relapse	Health tracking	Self-developed criteria	Acceptability	User	NR	NA
Fijacko et al. 2021 Slovenia	To evaluate the quality of apps that include mortality prediction models.	Observational cross-sectional study to review available apps	Android & iOS	Mortality risk prediction	Self-diagnosis of health conditions	uMARS	Engagement, functionality, aesthetics, information quality	HCP	NR	NA
Forman et al. 2019 USA	To evaluate the feasibility, acceptability, and preliminary effectiveness of a weight loss app.	Longitudinal user test of an app (quantitative survey)	iOS	Dietary lapses	Health tracking; Self-management	Technology Acceptance Model Scale	Satisfaction, perceived usefulness, usability, technical problems	User	TAM [3]	Evaluation
Fuller-Tyszkiewicz et al. 2018 Australia	To evaluate usability of a personalized, self-guided, app-based intervention for depression.	Longitudinal user test of an app (mixed-method)	NA	Depression	Health information seeking; Health tracking; Self-management	SUS; Qualitative open-ended questions	Usability	User & researcher	NR	NA

Gartenberg et al. 2013 USA	To describe the development of an app for insomnia treatment that is easy to use and has a high perceived benefit.	App development and evaluation	iOS	Insomnia and sleep-related problems	Health tracking; Self-management	Hierarchical Task Analysis, User satisfaction evaluation survey	Problem identification	Developer & user	Goals, Operators, Methods, and Selection rules (GOMS) modeling method [19]; Cognitive Behavior Therapy (CBT) [20]	Evaluation
Goetz et al. 2017 Germany	To analyze perceptions and requirements for pregnancy apps from patients' perspective and their impact on daily clinical routine.	Qualitative interviews	NA	Pregnancy	NA	Self-developed criteria	Usability, acceptability, impact on HCP-patient interaction	User	NR	NA
Gomes et al. 2022 Portugal	To present the results of performed usability tests for both web and mobile software applications of the Help2Care platform to train caregivers.	Cross-sectional user test of an app (mixed-method)	NA	Caregiver training	Health information-seeking; Link to health system	Self-developed questionnaire	Usability, suggestions for improvement	User	NR	NA
Gorini et al. 2018 Italy	To analyze variables that influence mHealth acceptance / adoption and to promote guidelines for future resources and apps.	Approach development, not clearly reported	Android, Blackberry OS, iOS & Windows	Chronic conditions	NA	P5 mHealth approach	Supporting innovative and personalized ways to improve quality of care	Developer	P5 medicine [21]	Development
Grau et al. 2016 Spain	To develop a tool for evaluating the reliability of health apps.	Review & Delphi survey	Android & iOS	NA	NA	iSYScore	Popularity & interest, trust & quality, useful	User	NR	NA
Groen et al. 2022 Zambia & South Africa	To describe the development and feasibility assessment of a mobile screening application for detecting mental disorders among adolescents.	App development and evaluation	Android	Mental health problems among adolescents	Health information-seeking	MARS	Quality classification & rating	HCP	Areas of focus for feasibility studies [22]	Evaluation
Guan et al. 2019 China	To develop a reliable framework to assess content and behavior change strategies of asthma apps.	Review & Delphi survey	NA	Asthma	Individual agency; Health information-seeking	Asthma apps assessment	Information quality, behavior change strategies, design	HCP and researcher	Asthma apps assessment framework [23]	Evaluation
Hammond et al. 2021 Australia	To identify psychoedu-cational social anxiety apps, report descriptive and technical	Observational cross-sectional study to review available apps	Android & iOS	People with social anxiety	NA	MARS	Quality classification & rating	Researcher	NR	NA

	information and evaluate app quality.								
Hampton et al. 2020 UK	To assessed the usability of a dermatology app.	Longitudinal user test of an app (quantitative survey)	Android & iOS	Skin	Health tracking	Self-developed criteria	Usability	User	NR	NA
Hartzler et al. 2016 USA	To assess the acceptability, usability and utility of a self-developed app to guide design considerations.	App development and evaluation	iOS	Diabetes mellitus and depression	Social and community support; Health tracking; Self-management	SUS; Qualitative open-ended questions	Usability; Intention to use	User	NR	NA
Hsieh et al. 2018 USA	To develop a fall risk app and to determine its usability in healthy, older adults.	App development and evaluation	Android	Fall risk in health older adults	Self-diagnosis of health conditions	SUS; Qualitative open-ended questions	Usability	User	NR	NA
Islam et al. 2022 Australia	To systematically review and evaluate high-quality apps for diabetes medication adherence, and present their technical features.	Observational cross-sectional study to review available apps	Android & iOS	Diabetes medication adherence	NA	MARS	Quality classification & rating	Researcher	NR	NA
Jain et al. 2019 India	To develop an educational mobile app and pilot test in an Indian clinical setting.	App development and evaluation	Android	Heart patients	Health information-seeking	MARS; User satisfaction questionnaire	Quality classification & rating; Satisfaction	User, HCP & developer	NR	NA
Jeffrey et al. 2019 Australia	To evaluate the experiences, barriers and facilitators to app usage and determine recommendations to improve app use.	Qualitative interviews	Android & iOS	Type 2 Diabetes	NA	Qualitative interview guide	Ease of use, usefulness, acceptance, aesthetics	User	TAM [3]; Factors Influencing App Use (modified from Health Belief model & Health Information Technology Acceptance Model, HITAM) [24]	Evaluation
Jeon et al. 2016 South Korea	To develop and evaluate four apps that provide tailored nursing recommendations for metabolic syndrome management.	App development and evaluation	Android, iOS & Windows	Metabolic syndrom	Social and community support; Health tracking; Self-management	SUS; System Usability Questionnaire	Usability	Expert	NR	NA
Jiam et al. 2017 USA	To describe the development of an app used to communicate	App development and evaluation	iOS	Neuro-developmental disabilities	Peronal health tracking; Self-management;	Self-developed criteria	User interface, experience	NR	NR	NA

	and organize health care information.				Link to health system					
Jiang et al. 2022 China	To design and develop an app to provide remote Warfarin dose adjustment and to evaluate its usability	App development and evaluation	WeChat	Warfarin dose adjustment	Health information seeking; Self-management; Link to health system	SUS	Usability	User & HCP	NR	NA
Jin & Kim 2015 South Korea	To develop and evaluate an evaluation tool for health apps.	App development and evaluation	NA	NA	NA	Healthcare Smartphone Applications Rating Tool	Validity, reliability	User	Three types of evaluation factors (content, technology, interface design) [25]	Evaluation
Johnson et al. 2016 USA	To evaluate the extent of coverage, ease of use, and quality across mobile devices and operating systems of six selected mobile point-of-care tools.	App development and evaluation	Android & iOS	NA	Health information seeking	Criteria based on Healthcare Information and Management Systems Society and Nielsen (2011)	Breadth of coverage, quality of evidence, usability of design	HCP	International Classification of Diseases, Ninth Revision (ICD-9)	Evaluation
Jovičić et al. 2019 Serbia, Poland & Italy	To review and evaluate existing apps to manage laboratory medicine data.	Cross-sectional review of existing apps	Android, iOS & Windows	Management of laboratory medicine data	NA	MARS	Quality classification & rating	Researcher	NR	NA
Kabir et al. 2021 Australia	To assess the method and software quality of foot measurement apps, and to determine their potential for use in clinical practice.	Observational cross-sectional study to review available apps	Android & iOS	Foot measurement in pedorthic	NA	FootMARS	Usability, reliability, functionality, efficiency	Software developer	NR	NA
Kaliyadan et al. 2020 Saudi Arabia & India	To discuss categories and scientific validity of dermatology apps.	Cross-sectional review of existing apps	Android, iOS & Windows	Dermatology	NA	Unclear origin of the criteria used	App selection criteria	HCP	NR	NA
Karsalia & Malik 2022 USA	To evaluate the quality apps for pelvic organ prolapse and urinary incontinence, analyze user sentiment, and evaluate information understandability, quality and actionability.	Observational cross-sectional study to review available apps	Android & iOS	Pelvic organ prolapse and urinary incontinence	NA	Xcertia guidelines; DISCERN tool; Patient Education Materials Assessment Tool (PEMAT)	Operability, privacy, security, content, usability; Information quality	Researcher	NR	NA

Karasneh et al. 2020 Jordan	To identify and evaluate apps related to period tracking.	Systematic review	Android & iOS	Period tracking	NA	MARS	Quality classification & rating	Researcher	NR	NA
Kettlewell et al. 2018 UK	To explore barriers and enablers for the uptake and use of an app in clinical practice, identify potential adaptations, and evaluate a model for engagement.	Mixed-methods study (quantitative surveys, qualitative focus groups)	NA	Brain injury	Health tracking; Self-management; Link to health system	MARS; SUS	Quality classification & rating; Usability	All stakeholder	Behavior Change Wheel (BCW) [26]	Development
Knitza et al. 2019 Germany	To provide an overview of rheumatology apps, evaluate quality and describe for patients and rheumatologists.	Cross-sectional review of existing apps	Android & iOS	Chronic rheumatic diseases	NA	MARS	Quality classification & rating	Researcher, developer & HCP	NR	NA
Kwan et al. 2017 Canada	To discuss ethical issues, risks and benefits when implementing health-monitoring apps in clinical practice; and to provide guidance on health-monitoring apps.	Scoping Review	NA	Mental Health	Health tracking; Link to health system	Summary of ethical recommendation s	Guide ethical decision-making process	HCP	Canadian Code of Ethics for Psychologists [27]	Evaluation
Lagan et al. 2020 USA	To evaluate the most accessible apps for bipolar disorder.	Cross-sectional review of existing apps	iOS	Bipolar disorders	NA	Self-developed criteria	Accessibility, privacy, clinical foundation, engagement, interoperability	Researcher	American Psychiatric Association's App Evaluation Framework [28]	Evaluation
Lalloo et al. 2017 Canada	To characterize and evaluate the content and functionality of commercial pain self-management apps.	Cross-sectional review of existing apps	Android, iOS & Windows	Postoperative Pain	NA	Self-developed criteria	Content, HCP involvement, scientific research	Researcher	NR	NA
Langarizadeh et al. 2022 Iran	To design and evaluate a mobile-based nutrition education application for infertile women.	App development and evaluation	Android	Nutrition for infertile women	Health information-seeking	Questionnaire for User Interaction Satisfaction (QUIS) [29]	Usability	User	NR	NA
Lao et al. 2022 China	To understand usability and satisfaction of an app to improve cardiovascular risk factors and effects of cardiac rehabilitation for patients after percutaneous coronary intervention.	Longitudinal user test of an app (qualitative interviews)	NA	Cardiac rehabilitation after percutaneous coronary intervention	Health information-seeking; Health tracking; Link to health system	Self-developed questionnaire	User experience	User	Social Cognitive Theory [30]	Development

Lau et al. 2021 USA	To evaluate the quality of successful apps, and the relationships between objective app quality, subjective user ratings, and evidence-based behavior change techniques.	Observational cross-sectional study to review available apps	Android & iOS	Mental health	NA	MARS	Quality classification & rating	Researcher	NR	NA
Le Marne et al. 2018 Australia	To evaluate a newly developed app to educate and facilitate management of adolescents with epilepsy.	Longitudinal user test of an app (mixed-method)	Android & iOS	Adolescents with Epilepsy	Health information-seeking; Health tracking; Link to health system	MARS	Quality classification & rating	User	NR	NA
Li et al. 2020 China	To evaluate postpartum depression apps available in China.	Cross-sectional review of existing apps	Android & iOS	Postpartum depression	NA	MARS	Quality classification & rating	Researcher	NR	NA
Logsdon et al. 2019 USA	To develop an app prototype to increase a new mother's ability to monitor her own health.	App development and evaluation	NA	Mothers after childbirth	Health information-seeking; Health tracking	MARS	Quality classification & rating	User	NR	NA
Lorca-Cabrera et al. 2021 Spain	To analyze the technical and functional characteristics of apps for caregivers of chronically ill.	Observational cross-sectional study to review available apps	Android & iOS	Caregivers of patients with chronic conditions	NA	Self-developed questionnaire	Technical aspects, functionality	Researcher	NR	NA
Manning et al. 2021 Australia	To test feasibility, acceptability, and effectiveness of an alcohol avoidance app, and explore alcohol consumption and craving.	Longitudinal user test of an app (quantitative survey)	Android & iOS	Alcohol consumption at hazardous levels or above	Individual agency	uMARS	Acceptablity	User	NR	NA
Mansson et al. 2020 Sweden	To develop a self-test app for balance and leg strength in older adults.	App development and evaluation	Android	Balance and leg strength in older adults	Self-diagnosis of health conditions	Qualitative think aloud & group discussions (used questions unclear)	User experience	Developer	Optimized Honeycomb model for user experience [31]	Evaluation

Maramba et al. 2019 UK	To identify, explore, and summarize the current methods used in the usability testing of eHealth apps.	Scoping Review	NA	Mental health, cancer, nutrition, child health, diabetes, cardiovascular disease, HIV, smoking	NA	SUS; System Usability Questionnaire; TAM Questionnaire; Task Index; AdEQUATE (questionnAire for Evaluation of QUAlity in TElemedicine systems)	Usability	User & HCP	NR	NA
Martinengo et al 2022 Singapore	To systematically evaluate adherence to clinical guidelines on depression of the information offered by mental health apps available in major commercial app stores.	Observational cross-sectional study to review available apps	Android & iOS	Mental health	Health information-seeking	Self-developed questionnaire	Content analysis, technical aspects	Researcher	NR	NA
Martinez-Perez et al. 2013 Spain	To develop a tool to assess the Quality of Experience of mHealth apps to improve the quality of existing and new apps.	Systematic checklist development	Android, Blackberry OS & iOS	NA	NA	Quality of Experience survey	User experience	Developer	NR	NA
Martinez-Perez et al. 2015 Spain	To compare two different tools for assessing the quality of the Heartkeeper mHealth app for self-management of heart diseases.	Cross-sectional review of existing apps	Android	Heart diseases and conditions	Health information-seeking; Health tracking; Self-management	Quality of Experience survey; Android guidelines by Google	User experience	Developer	NR	NA
McKay et al. 2018 Australia	To investigate current best practice approaches for health app evaluation.	Systematic Review	Android, Blackberry OS, iOS & Windows	Health behavior change	NA	MARS; Coventry, Aberdeen & London-Revised taxonomy (CALO-RE); Flesch-Kincaid Grade Level readability formula	Quality classification & rating; Taxonomy of behavior change techniques; Content analysis	NR	Heuristic evaluation [2]; Behaviour change taxonomy [32]	Evaluation
McKay et al. 2019 Australia	To review and test healthy lifestyle apps in view of functionality and	Cross-sectional review of existing apps	iOS	Healthy lifestyle	NA	MARS;	Quality classification & rating;	Researcher & developer	NR	NA

	potential to encourage behavior change.					App Behavior Change Scale (ABACUS)	Potential for behavior change			
Mehdi et al. 2020 Germany	To providing an overview of existing tinnitus apps.	Cross-sectional review of existing apps	NA	Tinnitus	NA	MARS	Quality classification & rating	User and developer	NR	NA
Mobasheri et al. 2014 UK	To evaluate the evidence base and HCP involvement of apps for breast disease.	Cross-sectional review of existing apps	Android, Blackberry OS, iOS & Windows	Breast desease	NA	Self-developed criteria	Evidence base, degree of HCP involvement	Researcher	NR	NA
Naccache et al. 2021 France	To explore acceptability and user experience of an app prototype for adolescents with anorexia nervosa.	App development and evaluation	NA	Adolescents with anorexia nervosa	Individual agency; Health information seeking; Self-management	User Experience Questionnaire (UEQ)	User experience	User	Cognitive-behavioral therapy; Motivational interview strategies	Development
Naslund et al. 2016 Lebanon	To assess acceptability of a wearable device and app to support a weight loss intervention in patients with serious mental illness.	Longitudinal user test of an app (mixed-method)	Android & iOS	Serious mental illness	Social and community support; Health tracking; Self-management	Adapted Usability, Satisfaction, Ease of use (USE) [33] questionnaire	Usability, satisfaction	User	National Institutes of Health (NIH) Stage Model of Behavioral Therapies Research (for study design) [34]	Development
Negreiros et al. 2022 Brazil	To identify and evaluate fitness apps that were used to increase the level of physical activity and improve the overall health of healthy adulty.	Observational cross-sectional study to review available apps	NA	Physical activity in healthy adults	NA	MARS	Quality classification & rating	Researcher	NR	NA
Nquyen et al. 2021 Canada	To review diabetes apps' usability, features, clinical safety of insulin calculators and evaluate the quality of exportable blood glucose reports.	Observational cross-sectional study to review available apps	Android & iOS	Diabetes mellitus type 2	NA	MARS	Quality classification & rating	Researcher	NR	NA
Nicolaidou et al. 2022 Cyprus	To develop and evaluate a prototype for a gamified, theory-based mobile app to enhance undergraduate students' resilience.	App development and evaluation	NA	Mental health	Individual agency; Health information seeking	SUS	Usability	User	NR	NA
Nolen et al. 2018 USA	To develop and test an app prototype for the prevention of Early Childhood Caries.	App development and evaluation	iOS	Oral Health of children ≤6 years	Individual agency; Health information-seeking; Health tracking; Self-management	Self-developed criteria	Usability	User	Heuristic evaluation [2]	Evaluation

O'Reilly & Laws 2019 Austria	To evaluate a pilot app in view of the app functionality and messaging.	Cross-sectional user test of an app (qualitative focus groups)	Android & iOS	Women with prior gestational diabetes mellitus	Individual agency; Social and community support; Health tracking	Qualitative focus groups	Functionality, information quality	Developer	NR	NA
Olfert et al. 2019 USA	To examine the feasibility of a personalized nutrition tracking app.	Longitudinal user test of an app (mixed-method)	Android & iOS	Nutrition tracking	Health tracking; Self-management; Link to health system	Unclear origin of the criteria used	NR	HCP	NR	NA
Oyebode et al. 2020 Canada	To determine factors positively and negatively affecting the effectiveness of mental health apps.	Cross-sectional review of existing apps	Android & iOS	Mental Health	NA	Qualitative user reviews	Identification of factors affecting effectiveness	Developer	Machine learning	Evaluation
Potzel et al. 2021 Germany	To develop a theory- and evidence-based mHealth intervention to change risk behaviors in women during the first 5 years post-gestational diabetes mellitus.	App development and evaluation	iOS	Women after gestational diabetes mellitus	Health information-seeking; Health tracking; Self-management	SUS; uMARS	Usability; Engagement, functionality, aesthetics, information quality	User	NR	NA
Quinn et al. 2019 USA	To determine the usability of a mobile app in community-based older adults.	Longitudinal user test of an app (quantitative survey)	NA	Older adults (≥65 years)	Social and community support; Health tracking; Self-management; Link to health system	SUS; Modified MARS	Usability; Engagement, functionality, aesthetics	User	NR	NA
Reyes et al. 2018 Canada	To evaluate the quality of balance promoting apps and identify strengths and areas of concern.	Cross-sectional review of existing apps	Android & iOS	Balance promotion	NA	MARS	Quality classification & rating	Researcher	NR	NA
Ribeiro et al. 2017 Portugal	To field-test an app to promote cancer prevention behaviors.	Longitudinal user test of an app (mixed-method)	Android & iOS	Cancer prevention behaviors	Individual agency; Health information-seeking; Health tracking; Self-management	Adapted Five Usability Factors; adapted MARS	Usability, feasibility, information quality; Usefulness	User	NR	NA
Rivera et al. 2016 Canada	To characterize the inclusion of evidence-based strategies, HCP involvement, and scientific evaluation of commercial apps.	Cross-sectional review of existing apps	Android, Blackberry OS, iOS & Windows	Weight loss	NA	Self-developed criteria	Evidence-based strategies, HCP involvement, scientific evaluation	Researcher	NR	NA

Saeedi et al. 2016 Persia	To investigate and categorized 200 top Persian medical apps.	Cross-sectional review of existing apps	Android	NA	NA	Unclear origin of the criteria used	Content analysis	Researcher	NR	NA
Salmani et al. 2022 Iran	To develop and evaluate usability of a self-management app for patients with colorectal cancer.	App development and evaluation	Android	Colorectal cancer	Health information-seeking; Health tracking; Self-management	Questionnaire for User Interaction Satisfaction (QUIS)	Usability	User	NR	NA
Sanatkar et al. 2022 Australia	To investigate use and effectiveness of an app on depressive and anxiety symptoms, work and social functioning, safety concerns, and help seeking.	Longitudinal user test of an app (quantitative survey)	Android & iOS	Mental health	Health tracking	Self-developed questionnaire	Usability, acceptability	HCP	NR	NA
Sarzynski et al. 2017 USA	To evaluate an app for accuracy of medication dosing instructions, acceptability of user interface, and patients' medication adherence.	App development and evaluation	iOS	Medication management	Individual agency; Health information-seeking; Health tracking; Self-management	SUS; Qualitative open-ended questions	Usability; Suggestions for improvement	User	NR	NA
Schmidt et al. 2020 Germany	To monitor acceptance and usability of an app in patients with left ventricular assist device (LVAD).	Longitudinal user test of an app (quantitative survey)	Android	LVAD	Health tracking; Link to health system	Adapted app Evaluation Questionnaire	Acceptance, usability	User	NR	NA
Schults et al. 2019 Australia	To outline the evidence on the development, implementation and evaluation of apps in terms of feasibility, acceptability and impact on key pain outcomes.	Scoping Review	NA	Persistent pain (children and young adolescents)	NA	Unclear origin of the criteria used	Content analysis	User	NR	NA
Scott et al. 2017 USA	To gauge interest in app use among patients after colorectal surgery and understand factors affecting patient app use at home.	Longitudinal user test of an app (mixed-method)	Android, Blackberry OS & iOS	Patients after colorectal surgery	Health tracking; Self-management	SUS	Usability	User	NR	NA
Shahmoradi et al. 2021 Iran	To develop and evaluate a self-care app for patients with urinary tract stones.	App development and evaluation	Android	Urinary tract stones	Health information-seeking; Self-management	PSSUQ	Usability, satisfaction	Expert	Heuristic evaluation	Evaluation

Shalan et al. 2018 UK	To develop and evaluate a prototype to promote exercise and track changes in walking ability.	App development and evaluation	Android	Peripheral Arterial Disease	Individual Agency; Health tracking	SUS	Usability	HCP	User Centered Design [7]	Development
Shen et al. 2022 China	To develop a mobile app for technology-facilitated fluid balance monitoring and to determine its usability.	App development and evaluation	NA	Fluid balance monitoring in heart failure	Health-information-seeking; Health tracking	SUS	Usability	User & HCP	NR	NA
Sherwin et al. 2021 USA	To examine usability and feasibility of a medication reminder app for irritable bowel syndrome.	Longitudinal user test of an app (mixed-method)	Android & iOS	Medication adherence in irritable bowel syndrome	Self-management; Link to health system	USE questionnaire [33]	Usefulness, Satisfaction, Ease of use	User	NR	NA
Signorelli et al. 2022 USA	To examine the potential acceptability, feasibility and usability of a mobile-based intervention to promote physical activity in breast cancer patients.	Longitudinal user test of an app (mixed-method)	Android	Breat cancer	Health tracking; Self-management	SUS; uMARS	Usability; Engagement, functionality, aesthetics, information quality	User	Theoretical Framework of Acceptability (TFA)	Evaluation
Singh et al. 2019 USA	To identify and evaluate apps across the spectrum of chronic kidney disease care.	Cross-sectional review of existing apps	Android & iOS	Chronic kidney disease	NA	Self-developed criteria	Engagment, quality, safety, cost	NR	Framework for evaluating patient engagement, quality & safety of mobile health apps [35]	Evaluation
Songtaweesin et al. 2021 Thailand	To adapt an app to improve pre-expostition prophylaxis adherence and persistence for young men who have sex with men (MSM) in Thailand.	Cross-sectional user test of an app (qualitative interviews and focus groups)	Android & iOS	Pre-exposition prophylaxis adherence for young MSM	Health information-seeking; Health tracking; Self-management	Self-developed questionniare	Suggestions for improvement	User	NR	NA
Soomro et al. 2019 Australia	To describe development and evaluation of the TeamDoc app regarding its design, functionality and utility.	App development and evaluation	Android & iOS	Cricket injury surveillance	Health tracking; Link to health system	Modified MARS user version	Quality classification & rating	User	NR	NA
Sudol et al. 2019 USA	To identify and evaluate patient-centered apps in female pelvic medicine and reconstructive surgery.	Cross-sectional review of existing apps	Android & iOS	Female pelvic medicine and reconstructive surgery	NA	Modified APPLICATIONS scoring system	Content analysis	HCP	NR	NA

Torous et al. 2018 Australia, Canada, USA, UK	To provide a narrative review of various schemes toward app evaluations.	Narrative review	NA	Mental Health	NA	Collaborative Health App Rating Teams (CHART)	Interoperability, usability, evidence, risk / privacy / security	User, researcher, expert	American Psychiatric Association app rating framework; ASPECT [36]	Evaluation
Trecca et al. 2021 Italy	To systematically review otolaryngology apps for patients in mobile app stores and the current literature.	Observational cross-sectional study to review available apps	Android & iOS	Otolaryngolog y	NA	MARS	Quality classification & rating	Researcher	NR	NA
Vanderloo et al. 2021 Canada	To create a checklist to aid the members of a trial in searching, identifying, screening, and including selected eHealth resources for participant use in the study intervention.	Guidelines adaptation	Android & iOS	Behavior change in pediatry	NA	MARS	Quality classification & rating	Researcher	NR	NA
Vasiloglou et al. 2020 Switzerland	To examine the acceptability of *Nutrition & Diet* apps, explore feature preferences and identify predictors of acceptance.	Cross-sectional survey	Android, iOS & Windows	Nutrition and Diet	NA	Self-developed criteria	Acceptability, reliability, functionality	HCP	NR	NA
Vasiloglou et al. 2021 Switzerland	To explore the perspectives of end users on the features, current use, and acceptance of nutrition and diet mHealth apps.	Cross-sectional survey	iOS, Android & Windows	Nutrition and Diet	NA	Self-developed questionnaire	Functionality, acceptability, current use	User	NR	NA
Vokinger et al. 2020 Switzerland	To support critical assessment of the purposes and trustworthiness of COVID-19 apps.	Systematic Review	NA	COVID-19	NA	COVID-19 smartphone and web applications framework checklist	Purpose, trustworthi-ness	User & developer	mHealth App Trustworthiness checklist [37]	Evaluation
Werner-Seidler et al. 2019 Australia	To test the feasibility, acceptability and preliminary effects of an app.	Longitudinal user test of an app (mixed-method)	Android & iOS	Sleep difficulties	Health information-seeking; Health tracking; Self-management	Self-developed criteria	Acceptability	User	NR	NA
Winoker et al. 2021 USA	To evaluate the quality of patient-oriented, mobile health apps available for medical management of kidney stone disease.	Observational cross-sectional study to review available apps	Android & iOS	Kidney stone disease	NA	MARS	Quality classification & rating	Researcher	Johns Hopkins Digital Health Scorecard [38]	Evaluation

Wyatt et al. 2015 UK	To develop and pilot a checklist to assess medical apps.	Checklist development, not clearly reported	NA	NA	NA	Royal College of Physicians Health Informatics Unit clinical app quality checklist	Structure, function, impact	HCP	Donabedian's factors of medical care quality [39]	Evaluation
Yasini & Marchand 2015 France	To evaluate if usability scores correlate with medical safety.	Cross-sectional review of existing apps	Android & iOS	NA	NA	Self-developed criteria	Usability, medical safety	User, caregiver and HCP	NR	NA
Yong et al. 2021 Malaysia	To evaluate the acceptance of an app intervention to reduce child injury at home.	Longitudinal user test of an app (qualitative interviews)	Android	Child injury at home	Self-diagnosis of health conditions; Self-management	Self-developed questionnaire	Acceptability	User	Consolidated Framework for Implementation Research (CFIR)	Evaluation
Yu et al. 2021 USA	To examine immigrants' acceptability for adopting a mHealth app to facilitate diabetes 2 self-management.	Cross-sectional user test of an app (mixed-method)	Android	Diabetes type 2	Health tracking; Self-management	Self-developed questionnaire	Acceptability	User	UTAUT	Evaluation
Zapata et al. 2014 Spain	To analyze and assess free mobile personal health records regarding privacy and security.	Systematic Review	Android & iOS	NA	NA	Self-developed criteria	Privacy, security	Developer & stakeholder	Health Insurance Portability and Accountability Act (HIPAA) [40]	Evaluation
Zapata et al. 2015 Spain	To investigate usability evaluation processes described in studies on mHealth apps.	Systematic Review	Android, iOS & Windows	NA	NA	Quality assessment checklist	Usability	Researcher & developer	ISO/IEC 9126–1 [41] ISO/IEC 25010:2011 [42]	Evaluation
Zarnowiecki et al. 2020 Australia	To evaluate nutrition promotion via websites and apps for parents in view of evidence base, parent experience and commercial offerings.	Systematic Review	Android & iOS	Parents influencing children's nutrition	NA	MARS	Quality classification & rating	Researcher	NR	NA

[a]Abbreviations: BCW = Behavior Change Wheel; HCP = Health care professional; HON = Health On the Net; ISO = International Organization for Standardization; LVAD = left ventricular assist device; MARS = Mobile Application Rating Scale; MSM = men who have sex with men; NA = Not applicable; NR = Not reported; SUS = System Usability Scale; TAM = Technology Acceptance Model; UK = United Kingdom; USA = United States of America; USE = Usefulness, Satisfaction, Ease of use questionnaire

References

1. Grundy QH, Wang Z, Bero LA. Challenges in assessing mobile health app quality: a systematic review of prevalent and innovative methods. American Journal of Preventive Medicine; 2016;51(6):1051-9.
2. Nielsen J, Molich R, editors. Heuristic evaluation of user interfaces. Proceedings of the SIGCHI conference on Human factors in computing systems; 1990.
3. Davis FD. User acceptance of information systems: the technology acceptance model (TAM). 1987.
4. Venkatesh V, Morris MG, Davis GB, Davis FD. User acceptance of information technology: Toward a unified view. MIS quarterly; 2003:425-78.
5. Waugaman A. From principle to practice: implementing the principles for digital development. Proceedings of the Principles for Digital Development Working Group; 2016;4.
6. Huckvale K, Car M, Morrison C, Car J. Apps for asthma self-management: a systematic assessment of content and tools. BMC medicine; 2012;10(1):1-11.
7. Schnall R, Mosley JP, Iribarren SJ, Bakken S, Carballo-Diéguez A, Brown III W. Comparison of a user-centered design, self-management app to existing mHealth apps for persons living with HIV. JMIR mHealth and uHealth; 2015;3(3):e4882.
8. Radloff LS. The CES-D scale: A self-report depression scale for research in the general population. Applied psychological measurement; 1977;1(3):385-401.
9. Osman A, Bagge CL, Gutierrez PM, Konick LC, Kopper BA, Barrios FX. The Suicidal Behaviors Questionnaire-Revised (SBQ-R): validation with clinical and nonclinical samples. Assessment; 2001;8(4):443-54.
10. International Organization for Standardization. Systems and software engineering — Life cycle processes — Requirements engineering (ISO standard no. 29148:2011) 2011 [Available from: https://www.iso.org/standard/45171.html.
11. Toval A, Nicolás J, Moros B, García F. Requirements reuse for improving information systems security: a practitioner's approach. Requirements Engineering; 2002;6(4):205-19.
12. Meyer IH. Minority stress and mental health in gay men. Journal of health and social behavior; 1995:38-56.
13. Ordre des conseillers et conseillères d'orientation et des psychoéducateurs et psychoéducatrices du Québec. Normes de pratique du testing en psychologie et en éducation. Montréal, Qc: Institut de recherches psychologiques; 2003.
14. Android. Up and running with material design 2016 [Available from: https://developer.android.com/design/index.html.
15. Apple. iOS Human Interface Guidelines 2016 [Available from: https://developer.apple.com/design/human-interface-guidelines/ios/overview/design-principles/.
16. Microsoft. Design & U. I. 2016 [Available from: https://learn.microsoft.com/en-us/windows/apps/design/.
17. Prochaska JO, DiClemente CC. Stages and processes of self-change of smoking: toward an integrative model of change. Journal of consulting and clinical psychology; 1983;51(3):390.
18. Venkatesh V, Thong JY, Xu X. Consumer acceptance and use of information technology: extending the unified theory of acceptance and use of technology. MIS quarterly; 2012:157-78.
19. Card SK, Moran TP, Newell A. The psychology of human-computer interaction. Hillside. NJ: Lawrence Erlbaum Associates; 1983.

20. Morin CM, Bootzin RR, Buysse DJ, Edinger JD, Espie CA, Lichstein KL. Psychological and behavioral treatment of insomnia: update of the recent evidence (1998–2004). Sleep; 2006;29(11):1398-414.
21. Gorini A, Pravettoni G. P5 medicine: a plus for a personalized approach to oncology. Nature Reviews Clinical Oncology; 2011;8(7):444-.
22. Bowen DJ, Kreuter M, Spring B, Cofta-Woerpel L, Linnan L, Weiner D, et al. How we design feasibility studies. American journal of preventive medicine; 2009;36(5):452-7.
23. Guan Z, Sun L, Xiao Q, Wang Y. Constructing an assessment framework for the quality of asthma smartphone applications. BMC Med Inform Decis Mak; 2019;19(1):192.
24. Jeffrey B, Bagala M, Creighton A, Leavey T, Nicholls S, Wood C, et al. Mobile phone applications and their use in the self-management of Type 2 Diabetes Mellitus: a qualitative study among app users and non-app users. Diabetol Metab Syndr; 2019;11:84.
25. Jin M, Kim J. Development and evaluation of an evaluation tool for healthcare smartphone applications. Telemedicine and e-Health; 2015;21(10):831-7.
26. Michie S, Van Stralen MM, West R. The behaviour change wheel: a new method for characterising and designing behaviour change interventions. Implementation science; 2011;6(1):1-12.
27. Pettifor J, McCarron M, Schoepp G, Stark C, Stewart D. CANADIAN PSYCHOLOGICAL ASSOCIATION. 2000.
28. Torous JB, Chan SR, Gipson SYT, Kim JW, Nguyen TQ, Luo J, et al. A Hierarchical Framework for Evaluation and Informed Decision Making Regarding Smartphone Apps for Clinical Care. Psychiatr Serv; 2018;69(5):498-500.
29. Slaughter L, Harper B, Norman K, editors. Assessing the equivalence of the paper and on-line formats of the QUIS 5.5. Proceedings of Mid Atlantic Human Factors Conference; 1994.
30. Bandura A. Health promotion by social cognitive means. Health education & behavior; 2004;31(2):143-64.
31. Karagianni K. Optimizing the UX Honeycomb—A Small Amendment to the Classic Diagram Hopefully Improves Its UX. UX Collective; 2021.
32. Michie S, Richardson M, Johnston M, Abraham C, Francis J, Hardeman W, et al. The behavior change technique taxonomy (v1) of 93 hierarchically clustered techniques: building an international consensus for the reporting of behavior change interventions. Annals of behavioral medicine; 2013;46(1):81-95.
33. Gao M, Kortum P, Oswald F, editors. Psychometric evaluation of the use (usefulness, satisfaction, and ease of use) questionnaire for reliability and validity. Proceedings of the human factors and ergonomics society annual meeting; 2018: SAGE Publications Sage CA: Los Angeles, CA.
34. Onken LS, Carroll KM, Shoham V, Cuthbert BN, Riddle M. Reenvisioning clinical science: unifying the discipline to improve the public health. Clinical Psychological Science; 2014;2(1):22-34.
35. Singh K, Diamantidis CJ, Ramani S, Bhavsar NA, Mara P, Warner J, et al. Patients' and Nephrologists' Evaluation of Patient-Facing Smartphone Apps for CKD. Clin J Am Soc Nephrol; 2019;14(4):523-9.
36. Torous JB, Chan SR, Yellowlees PM, Boland R. To use or not? Evaluating ASPECTS of smartphone apps and mobile technology for clinical care in psychiatry. The Journal of clinical psychiatry; 2016;77(6):6729.
37. van Haasteren A, Gille F, Fadda M, Vayena E. Development of the mHealth App Trustworthiness checklist. Digital health; 2019;5:2055207619886463.
38. Mathews SC, McShea MJ, Hanley CL, Ravitz A, Labrique AB, Cohen AB. Digital health: a path to validation. NPJ digital medicine; 2019;2(1):1-9.

39. Donabedian A. The Quality of Medical Care: Methods for assessing and monitoring the quality of care for research and for quality assurance programs. Science; 1978;200(4344):856-64.
40. Anderson DR. Health and Human Services Standards for Privacy of Individually Identifiable Health Information. US Att'ys Bull; 2002;50:16.
41. International Organization for Standardization. Software engineering — Product quality — Part 1: Quality model (ISO standard no. 9126-1:2001) 2001 [Available from: https://www.iso.org/standard/22749.html.
42. International Organization for Standardization. Systems and software engineering — Systems and software Quality Requirements and Evaluation (SQuaRE) — System and software quality models (ISO standard no. 25010:2011) 2011 [Available from: https://www.iso.org/standard/35733.html.
