# Supplementary material for: Developing a Comprehensive List of Criteria to Evaluate the Characteristics and Quality of eHealth Smartphone Apps: Systematic Review
Source: JMIR Mhealth Uhealth. 2024 Jan 15;12:e48625. doi: 10.2196/48625 (PMC10825776; doi:10.2196/48625)
Supplement: Multimedia Appendix 4 [file mhealth_v12i1e48625_app4.pdf]

This is a Multimedia Appendix to a full manuscript published in the J Med Internet Res. For full copyright and citation information see http://dx.doi.org/10.2196/48625

Characteristics of studies included

Table A4. Detailed characteristics of studies included in the review

		N (%)
**Location**		
	USA	32 (25.0%)
	Australia	16 (12.5%)
	UK	11 (8.6%)
	Canada	10 (7.8%)
	Spain	8 (6.3%)
	China	5 (3.9%)
	Germany	5 (3.9%)
	Switzerland	4 (3.1%)
	Iran	3 (2.3%)
	Africa	2 (1.6%)
	Multiple countries	7 (5.5%)
**Design**		
	Cross-sectional review of existing apps	42 (32.8%)
	Reports on various app development and evaluation approaches	29 (22.7%)
	Quantitative, qualitative cross-sectional or longitudinal user testing of a single app	27 (21.1%)
	Literature reviews<br>- Systematic review<br>- Scoping review<br>- Review combined with Delphi survey<br>- Narrative review	 7 (5.5%)<br>5 (3.9%)<br>2 (1.6%)<br>1 (0.8%)
	Systematic checklist development (i.e., based on literature review and expert input)	3 (2.3%)
	Cross-sectional survey	3 (2.3%)
	Qualitative interview	2 (1.6%)
	Mixed-methods approach	2 (1.6%)
	Randomized controlled trial	1 (0.8%)
	Not clearly reported	4 (3.1%)
**Operating system**		
	Android	21 (16.4%)
	iOS	11 (8.6%)
	WeChat	1 (0.8%)
	iOS & Android	50 (39.1%)
	Other combinations	17 (13.3%)
	No focus on any specific operating system	28 (21.9%)
**Topic/condition**		
	Mental health	23 (18.0%)
	No specific focus / >1 indication	19 (14.8%)
	Health promotion, e.g., nutrition, weight loss, sleep	17 (13.3%)
	Heart diseases & conditions	7 (5.5%)
	Gynecology, pregnancy and postpartum period	7 (5.5.%)
	Oncology	6 (4.7%)
	Parenting/family health	6 (4.7%)
	Diabetes	5 (3.9%)
	Medication adherence/management	5 (3.9%)
	Nephrology	4 (3.1%)
	Support of caregivers	4 (3.1%)
	Support of HCP, e.g., postoperative care, management of lab data	4 (3.1%)

	Unspecified chronic diseases	3 (2.3%)
	Balance and fall prevention	3 (2.3%)
	Neurological issues in childhood & adolescence	3 (2.3%)
	Covid-19	2 (1.6%)
	Dermatology	2 (1.6%)
	Orthopaedic	2 (1.6%)
	Asthma	1 (0.8%)
	Accidental injuries	1 (0.8%)
	Allergy	1 (0.8%)
	Brain injury	1 (0.8%)
	Otolaryngology	1 (0.8%)
	Tinnitus	1 (0.8%)
**Main purpose of intervention**[a]		
	Personal health tracking	41 (32.0%)
	Health information-seeking	31 (24.2%)
	Self-management of health	29 (22.7%)
	Individuals' linkage to their health systems	17 (13.3%)
	Individual agency	9 (7.0%)
	Social and community support	6 (4.7%)
	Self-diagnosis of health conditions	6 (4.7%)
**Evaluation tools**		
	Mobile App Rating Scale (MARS)	33 (25.8%)
	Investigator-developed criteria	30 (23.4%)
	System Usability Scale (SUS)	22 (17.2%)
	Adapted versions of MARS	8 (6.3%)
	System Usability Questionnaire	2 (1.6%)
	Questionnaire for User Interaction Satisfaction (QUIS)	2 (1.6%)
	Quality of Experience (QoE) survey	2 (1.6%)
**Focus of the tool**		
	Usability	65 (45.8%)
	Quality classification and ratings in general	37 (26.1%)
	Functionality	18 (12.7%)
	Acceptability/feasibility (often not clearly differentiated)	17 (12.0%)
	Information quality	14 (9.9%)
	Underlying/supporting theories/evidence	12 (8.5%)
	Usefulness	12 (8.5%)
	Satisfaction	10 (7.0%)
	Engagement	9 (6.3%)
	Technical matters (e.g., infrastructure/ interoperability)	9 (6.3%)
	Security/privacy	7 (4.9%)
	Design/aesthetics	7 (4.9%)
	User experience	7 (4.9%)
	Content analysis	7 (4.9%)
	Clinician/academic involvement	5 (3.5%)
	Intention to use	4 (2.8%)
	Validity/reliability or behavior change	4 (2.8%)
**Target tool users**		
	End users (e.g., patients or caregivers)	44 (34.4%)
	Researchers	34 (26.6%)
	HCPs	15 (11.7%)
	Software developers	7 (5.5%)
	Experts (not specified)	2 (1.6%)
	Certification company	1 (0.8%)
	>1 stakeholder group	22 (17.1%)
	unclear	3 (2.3%)

**Theoretical frameworks**		
	No clear theoretical underpinning reported	83 (64.8%)
	Various non-eHealth-specific behavioral/social/implementation theories	10 (16.9%)
	Technology Acceptance Model (TAM)	7 (11.9%)
	Heuristic evaluation	5 (8.5%)
	Models of the International Organization for Standardization (ISO)	3 (5.1%)
	(extended) Unified Theory of Acceptance and Use of Technology (UTAUT/UTAUT2)	3 (5.1%)
	User Centered Design	2 (3.4%)

[a]*Classified according to the World Health Organization's classification for self-care interventions for health and well-being [4]. Only 65 studies (50.8%) provided enough detail to classify them.*
