# Supplementary material for: Developing a Comprehensive List of Criteria to Evaluate the Characteristics and Quality of eHealth Smartphone Apps: Systematic Review
Source: JMIR Mhealth Uhealth. 2024 Jan 15;12:e48625. doi: 10.2196/48625 (PMC10825776; doi:10.2196/48625)
Supplement: Multimedia Appendix 5 [file mhealth_v12i1e48625_app5.pdf]

This is a Multimedia Appendix to a full manuscript published in the J Med Internet Res. For full copyright and citation information see http://dx.doi.org/10.2196/48625

Original frameworks and dimensions.

Table A5. List of original dimensions found in the eHealth-related frameworks of the included articles

Framework	Purpose of the framework	Dimensions
American Psychiatric Association's App Evaluation Framework [1]	To offer clinicians and patients an adaptable scaffold for informed decision making	• Gather background information<br>• Risk/Privacy and safety<br>• Benefit/Efficacy<br>• Engagement<br>• Data sharing
Android Design Guideline [2]	To support designers and developers in creating an app	• Animation<br>• Style<br>• Layout<br>• Components<br>• Patterns<br>• Usability
Areas of focus for feasibility studies [3]	To describe different areas of focus used in diverse types of feasibility studies conducted in the field of cancer prevention	Acceptability<br>• (User) Satisfaction<br>• Intent to continue use<br>• Perceived appropriateness<br>• Fit within organizational culture<br>• Perceived positive or negative effects on organization<br><br>Practicality<br>• Positive/negative effects on target participants<br>• Ability of participants to carry out intervention activities<br>• Cost analysis
ASPECT framework [4]	To spark discussion about apps and aid clinicians in determining if an app is ASPECT	• Actionable<br>• Secure<br>• Professional<br>• Evidence-based<br>• Customizable<br>Transparent
Asthma apps assessment framework [5]	An index system to evaluate asthma mHealth apps to select or design asthma apps systematically and scientifically, and to improve quality evaluation standards for apps for chronic and common diseases.	Knowledge<br>• Asthma knowledge<br><br>Skills training for effective self-management<br>• Non-pharmacological strategies<br><br>Behavior change strategies<br>• Goals and planning<br>• Feedback and monitoring<br>• Shaping knowledge<br>• Social support<br>• Prompts<br><br>Design principles<br>• Ease of use

		• Usability
Canadian code of ethics for psychologists [6]	Ethical principles, values, and standards to guide ethical decision making by professional psychologists.	4 hierarchically principles:<br>• Respect for the Dignity of Persons<br>• Responsible Caring<br>• Integrity in relationships<br>• Responsibility to society<br><br>Ethical considerations:<br>• Privacy policies for user data<br>• Secure storage & transmission of data<br>• Credible sources for app content<br>• Evidence for clinical benefit<br>• Obtaining informed consent<br>• Communication of conflicts of interest
Consolidated Framework for Implementation Research (CFIR) [7]	Description of the constructs associated with successful implementation. The constructs come from a range of 19 frameworks or related theories. The CFIR summarized the range of construct terminology and definitions in a regulatory framework.	Potential barriers and facilitators of implementation or intervention outcomes:<br>• Characteristics of an intervention<br>• Outer setting<br>• Inner setting<br>• Characteristics of individuals<br>• Implementation process
Dimensions of evaluation criteria for mental health mobile apps [8]	To encourage the development of professional guidelines and clinical frameworks for evaluating mobile mental health apps	• Usefulness dimension<br>• Integration and infrastructure dimension (incl. privacy and security)<br>• Usability dimension
Donabedian's factors of medical care quality [9]	To assess and monitor the quality of care for research an quality assurance programs.	• Structure<br>• Process<br>• Outcome
Factors Influencing App Use (modified from Health Belief model and Health Information Technology Acceptance Model, HITAM) [10]	Theoretical grounding for research into the consumer experience of mobile phone apps (focus on barriers and facilitators)	Individual perceptions<br>• Perceived susceptibility<br>• Perceived severity<br><br>Modifying factors<br>• Demographics<br>• Perceived threat<br>• Cues to action<br><br>Likelihood of Action<br>• Perceived benefits<br>• Minus perceived barriers<br>• Utilization of diabetes mobile phone application
Framework for Evaluating Patient Engagement, Quality and Safety of Mobile Health Applications [11]	Evaluating patient engagement, quality and safety of mHealth in chronic kidney disease	• General app-related information<br>• General medical information<br>• Patient engagement<br>• Quality<br>• Usability<br>• Safety

Goals, Operators, Methods, and Selection Rules (GOMS) Method [12]	A model of human performance to improve human-computer interaction efficiency by removing useless or unneeded interactions	• Goals<br>• Operators<br>• Methods<br>• Selection
Health Information Technology Acceptance Model (HITAM) [13]	To understand why users accept or reject a health-related technology, and how user acceptance can be improved through technology design.	Health zone<br>• Health status<br>• Health belief & concerns<br>• Behavioral beliefs<br><br>Information Zone<br>• Subjective norm<br>• Health information technology reliability<br>• Normative beliefs<br><br>Technology Zone<br>• Health information technology self-efficacy<br>• Efficacy beliefs<br><br>(Mediating process)<br>• Perceived threat<br>• Perceived usefulness<br>• Perceived ease of use
Health Insurance Portability and Accountability Act (HIPAA) [14]	Federal law that required creation of national standards to protect patient health information	• Confidentiality, integrity, and availability of electronic protected health information<br>• Detection and safeguard against threats to the security<br>• Protection against anticipated impermissible uses or disclosures<br>• Certification of compliance by their workforce
Heuristic evaluation [15]	To identify any problems associated with the design of user interfaces	• Simple and natural dialogue<br>• Speak the user's language<br>• Minimize user memory load<br>• Be consistent<br>• Provide feedback<br>• Provide clearly marked exits<br>• Provide shortcuts<br>• Good error messages<br>• Prevent errors
iOS Human Interface Guideline [16]	To support designers and developers in creating an app	• UI Design Basics<br>• Design strategies<br>• iOS Technologies<br>• UI Elements<br>• Icon and Image Design
iSYScore [17]	To evaluate reliability of a health app, useful criteria before downloading the app, give criteria to developers to improve the quality of their apps	• Popularity and interest<br>• Trust and quality<br>• Usefulness

ISO/IEC/IEE E 29148:2011 standard: Life cycle processes [18]	Life cycle processes — Requirements engineering	• Introduction<br>• References<br>• Verification<br>• Appendices<br>• Specific requirements
ISO/IEC 25010:2011: Systems & software engineering [19]	Systems and software Quality Requirements and Evaluation (SQuaRE) — System and software quality models	Quality in use model<br>• Effectiveness<br>• Efficiency<br>• Satisfaction<br>• Freedom from risk<br>• Context coverage<br><br>Product quality model<br>• Functional suitability<br>• Performance efficiency<br>• Compatibility; Portability<br>• Usability<br>• Reliability<br>• Security<br>• Maintainability
ISO/IEC 9126–1 standard: Software engineering [20]	Software engineering — Product quality — Part 1: Quality model	• Understandability<br>• Learnability<br>• Operability<br>• Attractiveness
Johns Hopkins Digital Health Scorecard [21]	To propose framework that could form the basis for evaluation of digital health solutions including the following domains: technical, clinical, usability, and cost	• Technical quality<br>• Usability<br>• Clinical value<br>• Privacy/security
mHealth App Trustworthiness (mHAT) checklist [22]	Checklist for mHealth app developers with features or actions required for trustworthy mHealth apps according to end-user opinions	• Informational content<br>• Organizational attributes<br>• Social influences<br>• Technology-related features<br>• User control
Microsoft Design and UI [23]	To support designers and developers in creating an app	• Design Basics<br>• Layout<br>• Controls and patterns<br>• Style<br>• Motion<br>• Shell<br>• Input & interactions<br>• Devices<br>• Usability
Modified HON Foundation principles [24]	To evaluate mHealth apps (promotional page, developer website if available, and the app itself)	• Information must be authoritative<br>• Purpose (of the app)<br>• Confidentiality<br>• Information documented, referenced and dated<br>• Justification of claims<br>• (App) contact details<br>• Funding<br>• Editorial & advertising policy

Multi-dimensional framework for assessing health app quality[25]	To assess health app quality to help researchers select quality measures that account for multiple perspectives	• Scientific/clinical basis<br>• Functionality<br>• Usability<br>• Accountability<br>• Impact<br>• Popularity
Optimized Honeycomb model for user experience [26]	To describe what makes a user good experience.	• Findable; Accessible<br>• Useable<br>• Desirable<br>• Credible<br>• Useful<br>• Valuable
Principles for digital development [27]	To guide organizations & developers in integrating best practices into design	• Design with the user<br>• Understand existing ecosystem<br>• Design for scale<br>• Build for sustainability<br>• Be data driven<br>• Use open standards, data, source, and innovation<br>• Reuse and improve<br>• Address privacy and security<br>• Be collaborative
P5 approach to medicine [28]	To find innovative and personalized ways to improve the overall quality of care	• Predictive<br>• Personalized<br>• Preventive<br>• Participatory (social features)<br>• Psycho-educative (UCD)
RCP Health Informatics Unit clinical app quality checklist [29]	To assess the structure, function and impact of medical apps	• Developer of the app & content<br>• Performance<br>• Evidence of outcome improvement
SImple REuse of software requiremeNts (SIREN) methodology [30]	To identify systems descriptions that can be reused (in whole or in part) with a minimal number of changes, reducing the overall development effort.	• System requirements specification<br>• System test specification<br>• Interface requirements specification<br>• Software requirements specification<br>• Software test specification
Technology Acceptance Model (TAM) [31]	To understand why users accept or reject a given technology, and how user acceptance can be improved through technology design.	• External variables<br>• Perceived usefulness<br>• Perceived ease of use<br>• Attitude toward using<br>• Behavioral intention to use<br>• Actual system use
Theoretical Framework of Acceptability (TFA) [32]	A multi-construct theoretical framework of acceptability of health care interventions to assess prospective and retrospective acceptability from the perspective of intervention delivers and recipients.	Influences of acceptability:<br>• Affective attitude<br>• Burden<br>• Ethicality<br>• Intervention coherence<br>• Opportunity costs<br>• Perceived effectiveness<br>• Self-efficacy

Testing standards of practice (OCCOPPQ) and standards for mobile device interfaces [33]	To allow for a qualitative assessment of mobile applications.	• Accuracy of the assessment tool<br>• Intuitiveness of its use<br>• Scientific basis<br>• Visual aesthetics<br>• Language<br>• Advertising<br>• Presence of a user manual<br>• Management of results (their accuracy)<br>• Protected access to data
Three types of evaluation factors [34]	To evaluate health care smartphone apps	• Contents<br>• Technology<br>• Interface design
Unified Theory of Acceptance and Use of Technology (UTAUT) [35]	To understand why users accept or reject a given technology	• Performance expectancy<br>• Effort expectancy<br>• Social influence<br>• Facilitating conditions<br>• Influencing factors
extended Unified Theory of Acceptance and Use of Technology (UTAUT2) [36]	To extend the UTAUT to study acceptance and use of technology in a consumer context.	• Performance expectancy<br>• Effort expectancy<br>• Social influence<br>• Facilitating conditions<br>• Hedonic motivation<br>• Price value<br>• Habit<br>• Influencing factors (age, gender, experience)<br>• Behavioral intention<br>• Use Behavior
User-centered design [37]	A design process to define end users' key components of apps.	• End user involvement<br>• Iterative design process

References

1. Torous JB, Chan SR, Gipson SYT, Kim JW, Nguyen TQ, Luo J, et al. A Hierarchical Framework for Evaluation and Informed Decision Making Regarding Smartphone Apps for Clinical Care. Psychiatr Serv; 2018;69(5):498-500.
2. Android. Up and running with material design 2016 [Available from: https://developer.android.com/design/index.html.
3. Bowen DJ, Kreuter M, Spring B, Cofta-Woerpel L, Linnan L, Weiner D, et al. How we design feasibility studies. American journal of preventive medicine; 2009;36(5):452-7.
4. Torous JB, Chan SR, Yellowlees PM, Boland R. To use or not? Evaluating ASPECTS of smartphone apps and mobile technology for clinical care in psychiatry. The Journal of clinical psychiatry; 2016;77(6):6729.
5. Guan Z, Sun L, Xiao Q, Wang Y. Constructing an assessment framework for the quality of asthma smartphone applications. BMC Med Inform Decis Mak; 2019;19(1):192.
6. Pettifor J, McCarron M, Schoepp G, Stark C, Stewart D. CANADIAN PSYCHOLOGICAL ASSOCIATION. 2000.
7. Damschroder LJ, Aron DC, Keith RE, Kirsh SR, Alexander JA, Lowery JC. Fostering implementation of health services research findings into practice: a consolidated framework for advancing implementation science. Implementation science; 2009;4(1):1-15.
8. Chan S, Torous J, Hinton L, Yellowlees P. Towards a framework for evaluating mobile mental health apps. Telemedicine and e-Health; 2015;21(12):1038-41.
9. Donabedian A. The Quality of Medical Care: Methods for assessing and monitoring the quality of care for research and for quality assurance programs. Science; 1978;200(4344):856-64.
10. Jeffrey B, Bagala M, Creighton A, Leavey T, Nicholls S, Wood C, et al. Mobile phone applications and their use in the self-management of Type 2 Diabetes Mellitus: a qualitative study among app users and non-app users. Diabetol Metab Syndr; 2019;11:84.
11. Singh K, Diamantidis CJ, Ramani S, Bhavsar NA, Mara P, Warner J, et al. Patients' and Nephrologists' Evaluation of Patient-Facing Smartphone Apps for CKD. Clin J Am Soc Nephrol; 2019;14(4):523-9.
12. Card SK, Moran TP, Newell A. The psychology of human-computer interaction. Hillside. NJ: Lawrence Erlbaum Associates; 1983.
13. Kim J, Park H-A. Development of a health information technology acceptance model using consumers’ health behavior intention. J Med Internet Res; 2012;14(5):e2143.
14. Anderson DR. Health and Human Services Standards for Privacy of Individually Identifiable Health Information. US Att'ys Bull; 2002;50:16.
15. Nielsen J, Molich R, editors. Heuristic evaluation of user interfaces. Proceedings of the SIGCHI conference on Human factors in computing systems; 1990.
16. Apple. iOS Human Interface Guidelines 2016 [Available from: https://developer.apple.com/design/human-interface-guidelines/ios/overview/design-principles/.
17. Grau I, Kostov B, Gallego J, Grajales Iii F, Fernández-Luque L, Sisó-Almirall A. Método de valoración de aplicaciones móviles de salud en español: el índice iSYScore. SEMERGEN-Medicina de Familia; 2016;42(8):575-83.
18. International Organization for Standardization. Systems and software engineering — Life cycle processes — Requirements engineering (ISO standard no. 29148:2011) 2011 [Available from: https://www.iso.org/standard/45171.html.

19. International Organization for Standardization. Systems and software engineering — Systems and software Quality Requirements and Evaluation (SQuaRE) — System and software quality models (ISO standard no. 25010:2011) 2011 [Available from: https://www.iso.org/standard/35733.html.
20. International Organization for Standardization. Software engineering — Product quality — Part 1: Quality model (ISO standard no. 9126-1:2001) 2001 [Available from: https://www.iso.org/standard/22749.html.
21. Mathews SC, McShea MJ, Hanley CL, Ravitz A, Labrique AB, Cohen AB. Digital health: a path to validation. NPJ digital medicine; 2019;2(1):1-9.
22. van Haasteren A, Gille F, Fadda M, Vayena E. Development of the mHealth App Trustworthiness checklist. Digital health; 2019;5:2055207619886463.
23. Microsoft. Design & U. I. 2016 [Available from: https://learn.microsoft.com/en-us/windows/apps/design/.
24. Huckvale K, Car M, Morrison C, Car J. Apps for asthma self-management: a systematic assessment of content and tools. BMC medicine; 2012;10(1):1-11.
25. Grundy QH, Wang Z, Bero LA. Challenges in assessing mobile health app quality: a systematic review of prevalent and innovative methods. American Journal of Preventive Medicine; 2016;51(6):1051-9.
26. Karagianni K. Optimizing the UX Honeycomb—A Small Amendment to the Classic Diagram Hopefully Improves Its UX. UX Collective; 2021.
27. Waugaman A. From principle to practice: implementing the principles for digital development. Proceedings of the Principles for Digital Development Working Group; 2016;4.
28. Gorini A, Pravettoni G. P5 medicine: a plus for a personalized approach to oncology. Nature Reviews Clinical Oncology; 2011;8(7):444-.
29. Wyatt JC, Thimbleby H, Rastall P, Hoogewerf J, Wooldridge D, Williams J. What makes a good clinical app? Introducing the RCP Health Informatics Unit checklist. Clinical Medicine; 2015;15(6):519.
30. Toval A, Nicolás J, Moros B, García F. Requirements reuse for improving information systems security: a practitioner's approach. Requirements Engineering; 2002;6(4):205-19.
31. Davis FD. User acceptance of information systems: the technology acceptance model (TAM). 1987.
32. Sekhon M, Cartwright M, Francis JJ. Acceptability of healthcare interventions: an overview of reviews and development of a theoretical framework. BMC health services research; 2017;17(1):1-13.
33. Ordre des conseillers et conseillères d'orientation et des psychoéducateurs et psychoéducatrices du Québec. Normes de pratique du testing en psychologie et en éducation. Montréal, Qc: Institut de recherches psychologiques; 2003.
34. Jin M, Kim J. Development and evaluation of an evaluation tool for healthcare smartphone applications. Telemedicine and e-Health; 2015;21(10):831-7.
35. Venkatesh V, Morris MG, Davis GB, Davis FD. User acceptance of information technology: Toward a unified view. MIS quarterly; 2003:425-78.
36. Venkatesh V, Thong JY, Xu X. Consumer acceptance and use of information technology: extending the unified theory of acceptance and use of technology. MIS quarterly; 2012:157-78.
37. Schnall R, Mosley JP, Iribarren SJ, Bakken S, Carballo-Diéguez A, Brown III W. Comparison of a user-centered design, self-management app to existing mHealth apps for persons living with HIV. JMIR mHealth and uHealth; 2015;3(3):e4882.
