# Supplementary material for: Developing a Comprehensive List of Criteria to Evaluate the Characteristics and Quality of eHealth Smartphone Apps: Systematic Review
Source: JMIR Mhealth Uhealth. 2024 Jan 15;12:e48625. doi: 10.2196/48625 (PMC10825776; doi:10.2196/48625)
Supplement: Multimedia Appendix 6 [file mhealth_v12i1e48625_app6.docx]

## Multimedia Appendix 6: Revised criteria.

Table A6. Original criteria and revised formulation. ^a^

|  | **Original criteria** | **Revised criteria** |
| --- | --- | --- |
| **A. CONTEXT  (ethical aspects)** |  |  |
|  | *Does the app contain misleading information, risks, or may otherwise cause harm to the users? If yes, what misleading information, risks, or harms?* [1-7] | *What are the known and estimated harms or risks for patients when implementing or not implementing the app?* [1-7] |
|  | *Does the app offer any benefits? If yes, which ones?* On which level (i.e., personal level, societal or population level)? [3, 8, 9] | *What are the known and estimated benefits for patients when implementing or not implementing the app?* On which level (i.e., personal level, societal or population level)? [3, 8, 9] |
| **A. CONTEXT  (legal aspects)** |  |  |
|  | *Does the app follow any laws, inter-/national regulations and/or (security) standards* (e.g., MDR/CE mark, GDPR, HIPAA, ISO)? [3, 8, 10-14] | *What inter-/national authorizations, standards and/or register listings does the app have* (e.g., MDR/CE mark, GDPR, HIPAA, ISO)? [3, 8, 10-14] |
| **A. CONTEXT  (social aspects)** |  |  |
|  | Do you think users feel comfortable using this app by themselves, or do you think they would need to enlist help from someone else (e.g., a caregiver, family, friends, forum)? [15-17] | Do you think users feel comfortable using this app by themselves *(according to the average literacy level)*, or do you think they would need to enlist help from someone else (e.g., a caregiver, family, friends, forum)? [15-17] |
|  | Does the app provide support in explaining the condition / disability to others? [18, 19] | Does the app provide users support in explaining their conditions / disabilities to others *(e.g., family, friends)*? [18, 19] |
|  | Does the app provide all the information, functions and capabilities *needed by and appropriate for* the target population? [1, 15, 19-31] | Does the app provide all the information, functions and capabilities *expected and wished for by* the target population? [1, 15, 19-31] |
|  | *How does* the app *affect* the clinician-patient relationship? [3, 9, 32-34] | *What is the estimated impact of* the app *on* the clinician-patient relationship? [3, 9, 32-34] |
|  | Does the app increase *access to services / information technology? [34-36]* | How does the app affect *access to health services? [34, 35]*  How does the app affect *access to information technology?* [36] |
| **B. STAKEHOLDER INVOLVEMENT** |  |  |
|  | Were HCP / clinicians involved in the app and/or content development process? If yes, which *people* were involved in which processes and how? [6, 9, 12, 37-43] | Were HCPs / clinicians involved in the app and/or content development process? If yes, which *types of stakeholders* were involved in which processes and how? [6, 9, 12, 37-43] |
|  | Were academics / researchers involved in app and/or content development process? If yes, which *people* were involved in which processes and how? [42, 43] | Were academics / researchers involved in app and/or content development process? If yes, which *types of stakeholders* were involved in which processes and how? [42, 43] |
| **D. IMPLEMENTATION (adoption)** |  |  |
|  | *What is your willingness to use* the app/specific functionalities in daily life (e.g., longer than 6 months)? [5, 17, 22, 23, 33, 44-52] | *Do you intend to use* the app/specific functionalities in daily life (e.g., longer than 6 months)? [5, 17, 22, 23, 33, 44-52] |
| **F. FEATURES & REQUIREMENTS (security)** |  |  |
|  | Does the app provide secure storage of user data (e.g., on the device or server, *country*, cloud backup, duration of data storage, data encryption / anonymization)? [1, 2, 6, 8-10, 18, 22, 26, 32, 36, 53, 54] | Does the app provide secure storage of user data (e.g., on the device or server, *country of server location, access to the server***,** cloud backup, duration of data storage, data encryption / anonymization)? [1, 2, 6, 8-10, 18, 22, 26, 32, 36, 53, 54] |

^a^adapted sections in italics.

**References**

1. Lagan S, Ramakrishnan A, Lamont E, Ramakrishnan A, Frye M, Torous J. Digital health developments and drawbacks: a review and analysis of top-returned apps for bipolar disorder. Int J Bipolar Disord; 2020;8(1):39.

2. Kaliyadan F, Ashique KT. Use of mobile applications in dermatology. Indian Journal of Dermatology; 2020;65(5):371.

3. Torous J, Firth J, Huckvale K, Larsen ME, Cosco TD, Carney R, et al. The emerging imperative for a consensus approach toward the rating and clinical recommendation of mental health apps. The Journal of nervous and mental disease; 2018;206(8):662-6.

4. Oyebode O, Alqahtani F, Orji R. Using machine learning and thematic analysis methods to evaluate mental health apps based on user reviews. IEEE Access; 2020;8:111141-58.

5. Collins R. Nurses’ perceived usefulness of secure texting applications for the purpose of patient care. Online Journal of Nursing Informatics; 2019;23(1).

6. Mobasheri MH, Johnston M, King D, Leff D, Thiruchelvam P, Darzi A. Smartphone breast applications–What's the evidence? The Breast; 2014;23(5):683-9.

7. Mansson L, Wiklund M, Öhberg F, Danielsson K, Sandlund M. Co-creation with older adults to improve user-experience of a smartphone self-test application to assess balance function. International journal of environmental research and public health; 2020;17(11):3768.

8. Vokinger KN, Nittas V, Witt CM, Fabrikant SI, von Wyl V. Digital health and the COVID-19 epidemic: an assessment framework for apps from an epidemiological and legal perspective. Swiss Med Wkly; 2020;150:w20282.

9. Kwan V, Hagen G, Noel M, Dobson K, Yeates K. Healthcare at your fingertips: the professional ethics of smartphone health-monitoring applications. Ethics & Behavior; 2017;27(8):615-31.

10. Zapata BC, Niñirola AH, Fernández-Alemán JL, Toval A, editors. Assessing the privacy policies in mobile personal health records. 2014 36th Annual International Conference of the IEEE Engineering in Medicine and Biology Society; 2014: IEEE.

11. Wyatt JC, Thimbleby H, Rastall P, Hoogewerf J, Wooldridge D, Williams J. What makes a good clinical app? Introducing the RCP Health Informatics Unit checklist. Clinical Medicine; 2015;15(6):519.

12. Boulos MNK, Brewer AC, Karimkhani C, Buller DB, Dellavalle RP. Mobile medical and health apps: state of the art, concerns, regulatory control and certification. Online journal of public health informatics; 2014;5(3):229.

13. Lorca-Cabrera J, Martí-Arques R, Albacar-Riobóo N, Raigal-Aran L, Roldan-Merino J, Ferré-Grau C. Mobile Applications for Caregivers of Individuals with Chronic Conditions and/or Diseases: Quantitative Content Analysis. Int J Med Inform; 2021;145:104310.

14. Vasiloglou MF, Christodoulidis S, Reber E, Stathopoulou T, Lu Y, Stanga Z, et al. Perspectives and Preferences of Adult Smartphone Users Regarding Nutrition and Diet Apps: Web-Based Survey Study. JMIR Mhealth Uhealth; 2021;9(7):e27885.

15. Hsieh KL, Fanning JT, Rogers WA, Wood TA, Sosnoff JJ. A Fall Risk mHealth App for Older Adults: Development and Usability Study. JMIR Aging; 2018;1(2):e11569.

16. Jeffrey B, Bagala M, Creighton A, Leavey T, Nicholls S, Wood C, et al. Mobile phone applications and their use in the self-management of Type 2 Diabetes Mellitus: a qualitative study among app users and non-app users. Diabetol Metab Syndr; 2019;11:84.

17. Caballero P, Ortiz G, Garcia-de-Prado A, Boubeta-Puig J. Paving the way to collaborative context-aware mobile applications: a case study on preventing worsening of allergy symptoms. Multimedia Tools and Applications; 2021;80(14):21101-33.

18. Jiam N, Hoon Jr AH, Hostetter C, Khare M. IIAM (important information about me): a patient portability profile app for adults, children and families with neurodevelopmental disabilities. Disability and Rehabilitation: Assistive Technology; 2017;12(6):599-604.

19. O'Reilly SL, Laws R. Health‐e mums: Evaluating a smartphone app design for diabetes prevention in women with previous gestational diabetes. Nutrition & dietetics; 2019;76(5):507-14.

20. Jeon E, Park H, Jo S, Kang H, Lee JY. Mobile apps providing tailored nursing interventions for patients with metabolic syndrome. Nursing Informatics 2016: IOS Press; 2016. p. 510-4.

21. Maramba I, Chatterjee A, Newman C. Methods of usability testing in the development of eHealth applications: A scoping review. Int J Med Inform; 2019;126:95-104.

22. Al Ayubi SU, Parmanto B, Branch R, Ding D. A persuasive and social mHealth application for physical activity: a usability and feasibility study. JMIR mHealth and uHealth; 2014;2(2):e2902.

23. Naslund JA, Aschbrenner KA, Bartels SJ. Wearable devices and smartphones for activity tracking among people with serious mental illness. Mental health and physical activity; 2016;10:10-7.

24. Stoyanov SR, Hides L, Kavanagh DJ, Zelenko O, Tjondronegoro D, Mani M. Mobile app rating scale: a new tool for assessing the quality of health mobile apps. JMIR mHealth and uHealth; 2015;3(1):e3422.

25. Jain YS, Garg A, Jhamb DK, Jain P, Karar A. Preparing India to Leverage Power of Mobile Technology: Development of a Bilingual Mobile Health Tool for Heart Patients. Cardiovasc Hematol Agents Med Chem; 2019;17(2):125-34.

26. Anderson K, Burford O, Emmerton L. App chronic disease checklist: protocol to evaluate mobile apps for chronic disease self-management. JMIR Res Protoc; 2016;5(4):e6194.

27. Bentvelsen RG, Holten E, Chavannes NH, Veldkamp KE. eHealth for the prevention of healthcare-associated infections: a scoping review. Journal of Hospital Infection; 2021;113:96-103.

28. Kabir MA, Rahman SS, Islam MM, Ahmed S, Laird C. Mobile Apps for Foot Measurement in Pedorthic Practice: Scoping Review. Jmir Mhealth and Uhealth; 2021;9(3).

29. Signorelli GR, Monteiro-Guerra F, Rivera-Romero O, Nunez-Benjumea FJ, Fernandez-Luque L. Breast Cancer Physical Activity Mobile Intervention: Early Findings From a User Experience and Acceptability Mixed Methods Study. JMIR Form Res; 2022;6(6).

30. Gao M, Kortum P, Oswald F, editors. Psychometric evaluation of the use (usefulness, satisfaction, and ease of use) questionnaire for reliability and validity. Proceedings of the human factors and ergonomics society annual meeting; 2018: SAGE Publications Sage CA: Los Angeles, CA.

31. Groen G, Jörns-Presentati A, Dessauvagie A, Seedat S, van den Heuvel LL, Suliman S, et al. Development of a Mobile Application for Detection of Adolescent Mental Health Problems and Feasibility Assessment with Primary Health Care Workers. Issues in Mental Health Nursing; 2022;43(11):1046-55.

32. Goetz M, Müller M, Matthies LM, Hansen J, Doster A, Szabo A, et al. Perceptions of patient engagement applications during pregnancy: a qualitative assessment of the patient’s perspective. JMIR mHealth and uHealth; 2017;5(5):e7040.

33. Eisner E, Drake RJ, Berry N, Barrowclough C, Emsley R, Machin M, et al. Development and Long-Term Acceptability of ExPRESS, a Mobile Phone App to Monitor Basic Symptoms and Early Signs of Psychosis Relapse. JMIR Mhealth Uhealth; 2019;7(3):e11568.

34. Lao SSW, Chair SY. The feasibility of smartphone-based application on cardiac rehabilitation for Chinese patients with percutaneous coronary intervention in Macau: a qualitative evaluation. Int J Qual Stud Health Well-being; 2022;17(1):2023940.

35. Olfert MD, Barr ML, Hagedorn RL, Long DM, Haggerty TS, Weimer M, et al. Feasibility of a mhealth approach to nutrition counseling in an Appalachian state. Journal of personalized medicine; 2019;9(4):50.

36. Vasiloglou MF, Christodoulidis S, Reber E, Stathopoulou T, Lu Y, Stanga Z, et al. What healthcare professionals think of “nutrition & diet” apps: an international survey. Nutrients; 2020;12(8):2214.

37. Ben-Mussa A, Paget AM. Popular apps on the medical category targeting patients and the general public in the United Kingdom: Do they conform to the Health On the Net Foundation principles? Health Informatics Journal; 2018;24(3):259-76.

38. Lalloo C, Shah U, Birnie KA, Davies-Chalmers C, Rivera J, Stinson J, et al. Commercially available smartphone apps to support postoperative pain self-management: scoping review. JMIR mHealth and uHealth; 2017;5(10):e8230.

39. Sudol NT, Adams-Piper E, Perry R, Lane F, Chen KT. In search of mobile applications for patients with pelvic floor disorders. Female Pelvic Medicine & Reconstructive Surgery; 2019;25(3):252-6.

40. Yasini M, Marchand G. Mobile health applications, in the absence of an authentic regulation, does the usability score correlate with a better medical reliability? MedInfo; 2015;216:127-31.

41. Saeedi MG, Kalhori SRN, Nouria R, Yasini M, Nouri R, Yasini M, editors. Persian mHealth apps: A cross sectional study based on use case classification. MIE; 2016.

42. Bennion MR, Hardy GE, Moore RK, Kellett S, Millings A. e-Therapies in England for stress, anxiety or depression: how are apps developed? A survey of NHS e-therapy developers. BMJ Health Care Inform; 2019;26(1).

43. Grau I, Kostov B, Gallego J, Fernández-Luque L, Sisó-Almirall A. Assessment method for mobile health applications in Spanish: The iSYScore index. Semergen; 2016;42(8):575-83.

44. Egan KJ, Hodgson W, Imperatore G, Dunlop MD, Maguire R, Kirk A. Supporting Physical Activity for Informal Caregivers during and beyond COVID-19: Exploring the Feasibility, Usability and Acceptability of a Digital Health Smartphone Application, 'CareFit'. Int J Environ Res Public Health; 2022;19(19).

45. Sanatkar S, Counson I, Mackinnon A, Bartholomew A, Glozier N, Harvey S. Preliminary Investigation of Shift, a Novel Smartphone App to Support Junior Doctors' Mental Health and Well-being: Examination of Symptom Progression, Usability, and Acceptability After 1 Month of Use. J Med Internet Res; 2022;24(9):e38497.

46. Yong TSM, Perialathan K, Ahmad M, Juatan N, Majid LA, Johari MZ. Perceptions and Acceptability of a Smartphone App Intervention (ChildSafe) in Malaysia: Qualitative Exploratory Study. Jmir Pediatrics and Parenting; 2021;4(2).

47. Bosse JD, Hoffman K, Wiest K, Todd Korthuis P, Petluri R, Pertl K, et al. Patient evaluation of a smartphone application for telehealth care of opioid use disorder. Addict Sci Clin Pract; 2022;17(1):50.

48. Scott AR, Alore EA, Naik AD, Berger DH, Suliburk JW. Mixed-methods analysis of factors impacting use of a postoperative mHealth app. JMIR mHealth and uHealth; 2017;5(2):e6728.

49. Brooke J. SUS-A quick and dirty usability scale. Usability evaluation in industry; 1996;189(194):4-7.

50. Hartzler AL, Venkatakrishnan A, Mohan S, Silva M, Lozano P, Ralston JD, et al., editors. Acceptability of a team-based mobile health (mHealth) application for lifestyle self-management in individuals with chronic illnesses. 2016 38th Annual International Conference of the IEEE Engineering in Medicine and Biology Society (EMBC); 2016: IEEE.

51. Werner-Seidler A, Wong Q, Johnston L, O'Dea B, Torok M, Christensen H. Pilot evaluation of the Sleep Ninja: a smartphone application for adolescent insomnia symptoms. BMJ Open; 2019;9(5):e026502.

52. Schmidt T, Mewes P, Hoffmann JD, Müller-von Aschwege F, Glitza JI, Schmitto JD, et al. Improved aftercare in LVAD patients: Development and feasibility of a smartphone application as a first step for telemonitoring. Artif Organs; 2020;44(3):248-56.

53. Chan S, Torous J, Hinton L, Yellowlees P. Towards a framework for evaluating mobile mental health apps. Telemedicine and e-Health; 2015;21(12):1038-41.

54. Martínez-Pérez B, de la Torre-Díez I, López-Coronado M, Rodrigues JJ, editors. Are mobile health cloud apps better than native? 2015 IEEE international conference on communications (ICC); 2015: IEEE.
