# Supplementary material for: Developing a Comprehensive List of Criteria to Evaluate the Characteristics and Quality of eHealth Smartphone Apps: Systematic Review
Source: JMIR Mhealth Uhealth. 2024 Jan 15;12:e48625. doi: 10.2196/48625 (PMC10825776; doi:10.2196/48625)
Supplement: Multimedia Appendix 7 [file mhealth_v12i1e48625_app7.docx]

## Multimedia Appendix 7: Additional criteria.

Table A7. Additional criteria.

| **A. CONTEXT (ethical aspects)** |  |
| --- | --- |
|  | Does the use of the app produce additional information that is not directly related to the current care of the patient and may violate their right to respect for privacy? [1] |
|  | What are the benefits and harms of the app for relatives, other patients, organizations, commercial entities, society, etc.? [1] |
|  | Does the implementation or use of the app affect the patient´s capability and possibility to exercise autonomy? [1] |
|  | Is there a need for any specific interventions or supportive actions concerning information in order to respect patient autonomy when the app is used? [1] |
|  | Does the implementation or use of the app affect the patient’s dignity, moral, religious or cultural integrity? [1] |
| **A. CONTEXT (legal aspects)** |  |
|  | Does the implementation or use of the app affect the realization of basic human rights? [1] |
| **A. CONTEXT (social aspects)** |  |
|  | Is the app used for/by individuals that are especially vulnerable? [1] |
|  | What expectations and wishes do patients have regarding the app and what do they expect to gain from it? [1, 2] |
|  | How do patients perceive the app? [1] |
|  | What is the app’s burden on caregivers? [1] |
|  | What specific issues may need to be communicated to patients to improve adherence? [1] |

**References**

1. EUnetHTA Joint Action. 2, Work Package 8. HTA Core Model® version 3.0. 2016.

2. Gao M, Kortum P, Oswald F, editors. Psychometric evaluation of the use (usefulness, satisfaction, and ease of use) questionnaire for reliability and validity. Proceedings of the human factors and ergonomics society annual meeting; 2018: SAGE Publications Sage CA: Los Angeles, CA.
