# Supplementary material for: Developing a Comprehensive List of Criteria to Evaluate the Characteristics and Quality of eHealth Smartphone Apps: Systematic Review
Source: JMIR Mhealth Uhealth. 2024 Jan 15;12:e48625. doi: 10.2196/48625 (PMC10825776; doi:10.2196/48625)
Supplement: Multimedia Appendix 8 [file mhealth_v12i1e48625_app8.pdf]

This is a Multimedia Appendix to a full manuscript published in the J Med Internet Res.
For full copyright and citation information see http://dx.doi.org/10.2196/48625

Complete criteria list

Table A8. Complete criteria list with all 216 criteria.

**DIMENSION /** sub-group	**Criteria**
**A. CONTEXT**	
	- Have measures been taken in terms of *context compatibility* (i.e., understanding the existing ecosystem and the needs of the target population through contextual analysis) and the corresponding real tasks (e.g., navigation in the real world))? [1-7]
Basic information	- App name [8-15]<br>- URL [8, 16]<br>- App store category (e.g., Medical, Health & Fitness, Lifestyle, Entertainment) [15-19]<br>- Platform availability (iOS, Android, other) [3, 6, 9-14, 16, 18, 20-25]<br>- Available languages [10, 18, 19, 21]<br>- Brief description, overview, product information available [3, 14, 16, 24, 26]<br>○ The description of the app is attractive and accurate [1, 14, 16, 21, 27-29]<br>- Presence of free “lite” version [12]<br>- Installation: in the mobile device or not required, as the main part of the app is in a cloud [25]<br>- App size (per platform) [10, 12, 21, 30]<br>- Version (per platform) [12-15, 21, 23]<br>- App store release date (per platform) [10, 17, 21]<br>- Date of last update (per platform) [10, 12, 14, 15, 18, 21, 25]<br>- What is the oldest platform version (e.g., iOS, Android) supported? [21]<br>- Number of downloads [9, 12, 16]
Ethical aspects	- Were ethical considerations taken into account in the development and implementation of the app? (unspecific criteria) [31]<br>- What are the known and estimated harms or risks for patients when implementing or not implementing the app? [5, 17, 21, 22, 30, 32, 33]<br>- What are the known and estimated benefits for patients when implementing or not implementing the app? On which level (i.e., personal level, societal or population level)? [8, 32, 34]<br>- Do the stated benefits depend on a certain number of persons using the app? [8]<br>- How much time is required for the user to derive some benefit from the app? [1]<br>- Overall, do the benefits of the app outweigh the harm, inconvenience and costs to the user? [13, 34]<br>- What are the benefits and harms of the app for relatives, other patients, organizations, commercial entities, society, etc.? [35]<br>- Do you have concerns about privacy, confidentiality and/or location of data storage? [34, 36-38]<br>- Does the use of the app produce additional information that is not directly related to the current care of the patient and may violate their right to respect for privacy? [35]<br>- Does the app obtain informed consent? [16, 34, 39]<br>- If data is collected and/or analyzed: Is there a clearly stated, understandable and meaningful purpose for data collection and/or analysis? [8, 32]<br>- Can users get information about the results of the data analysis? [8]<br>- Does the app not send unwanted messages (spams, excessive notifications) [30]

	- Is the payment pattern fair? [30]<br>- Does the apps seem to fragment care by siloing data? [32]<br>- Does the implementation or use of the app affect the patient´s capability and possibility to exercise autonomy? [35]<br>- Is there a need for any specific interventions or supportive actions concerning information in order to respect patient autonomy when the app is used? [35]<br>- Does the implementation or use of the app affect the patient's dignity, moral, religious or cultural integrity? [35]
Legal aspects	- What inter-/national authorizations, standards and/or register listings does the app have (e.g., MDR/CE mark, GDPR, HIPAA, ISO)? [8, 13, 18, 32, 39-41]<br>- Are there general terms and conditions of use for the app? [42]<br>- Does the app take into account patient safety, e.g., clear warning about the app's diagnostic limitations, the possibility and impact of "false-negative" results, mentioning the possibility (or absence of one) of substituting its results for medical advice; does it supply emergency contact information; is there any notable consideration of suicidality? [1, 5, 8, 12, 19, 21, 23, 30-32, 34, 39]<br>- Does the app inform the end user about the voluntary nature of participation? [8]<br>- Do end users act as the proprietors of the data generated by the app (i.e., does the app seek explicit end-user permission before sharing data with third parties?)? [8, 21, 43]<br>- Does the implementation or use of the app affect the realization of basic human rights? [35]
Social aspects	- Were social aspects taken into account in the app's development and implementation? (unspecific criteria) [4]<br>- Do you think users feel comfortable using this app by themselves (according to the average literacy level), or do you think they would need to enlist help from someone else (e.g., a caregiver, family, friends, or an online forum)? [36, 44, 45]<br>- Does an app community exist? [7, 14, 15, 19, 24]<br>- Does the app connect with other affected people, users or peers? [6, 20, 38, 43]<br>- Does the app offer the possibility to learn from other users? [37]<br>- Does the app provide users support in explaining their conditions / disabilities to others (e.g., family, friends)? [46, 47]<br>- Does the app allow multiple users, and if so, can access be granted and revoked? [40, 48]<br>- What expectations and wishes do patients have regarding the app and what do they expect to gain from it? [35, 49]<br>- Does the app provide all the information, functions and capabilities expected and wished for by the target population? [4, 11, 14, 16, 21, 29, 43, 44, 47, 49-54]<br>- How do patients perceive the app? [35]<br>- What is the app's burden on caregivers? [35]<br>- What specific issues may need to be communicated to patients to improve adherence? [35]<br>- Is the app recommended by a patient association or scientific society? [20]<br>- Would you recommend this app to patients / caregivers / people who might benefit from it? [9, 14, 16, 29, 36, 43, 55-59]<br>- How likely are you to recommend this app to a friend, family member or colleague? [27, 36, 37, 43, 49-51, 60-62]<br>- What is the estimated impact of the app on the clinician-patient relationship? [7, 32, 34, 48, 63]<br>- Is the app used for/by individuals that are especially vulnerable? [35]<br>- Is the app easily accessible to the target population even with disabilities through visual, auditory and/or motor support (e.g., adapted text size, screen

	readers, subtitles)? [1, 3, 8, 12, 21, 26, 33, 39, 50, 52, 64]<br>- Does the app take into account the user's socioeconomic status and age, and how these potentially affect their digital health literacy? [1, 11]<br>- How does the app affect access to health services? [27, 63]<br>- How does the app affect access to information technology? [65]
**B. STAKEHOLDER INVOLVEMENT**	
	- Were HCPs / clinicians involved in the app and/or content development process?<br>If yes, which types of stakeholders were involved in which processes and how? [12, 17, 20, 23, 31, 34, 39, 42, 66, 67]<br>- Were academics / researchers involved in app and/or content development process? If yes, which types of stakeholders were involved in which processes and how? [20, 67]
**C. DEVELOPMENT PROCESS**	
	- Does the app integrate third-party software or use third-party application programming interface (API)? [8]<br>- Were open standards, open data, open sources, and/or open innovation used in the development of the app? [2, 68]<br>- How was the app constructed? [25]<br>- What programming language was used in the development of the app? [25]<br>- Does it refer to continuing areas of uncertainty? [69]
Co-creation of technologies / User-centered design	- Were end-users (e.g., patients) involved in the app and/or content development process? If yes, which people were involved in which processes and how (e.g., 'user-centred design')? [2, 39, 66, 70]<br>- Is there a possibility to give feedback / user comments to the app developer? [13, 16, 22, 71]
Characteristics of the development team	- Who is/are the developer/s of the app (i.e., name, affiliation, country)? [8, 10, 12, 14-20, 23, 71, 72]<br>- What is the organization behind the app, its mission, and its purpose (e.g., profession/background, credentials and reputation; individual, non-profit, for profit, government agency, trusted health care company, academic institution)? [8, 9, 12, 14, 18, 19, 21, 29, 39]<br>- Credibility: Does the app come from a legitimate/acknowledged source (specified in app store description or within the app itself, e.g., uses a recognized logo or cites research)? [5, 11, 13, 14, 16, 29, 71, 73]<br>- Has the developer created similar apps in the past? [8, 12]<br>- Does the app and/or developer have an associated website? [12, 20]<br>- How much were the development costs? [25]<br>- How and by whom was the development funded and/or sponsored? [8, 12, 39]<br>- Does the app have commercial interests (i.e., profit orientation)? [8, 17]<br>- Are other potential conflicts of interest and/or external influences that could affect the objectivity of the content transparently disclosed? [12, 32, 34, 39]<br>- Are there in-app advertisements? If yes, for what (e.g., medical products, health care organizations, upcoming conferences, other)? [9, 10, 17-19, 23, 24, 30, 42, 72]<br>- If paid banners are shown in the app, is there an advertising policy, especially for advertising that concerns clinical or health-related products or services? This policy needs to explain how to distinguish between editorial and promotional content and what advertising is acceptable. [12, 39]
**D. IMPLEMENTATION**	
Adoption (integration into daily life)	- Perceived relevance / benefit of the app: What is the relevance of the content and functionalities, does it give control over physical activity in daily life and offer advantages over other means? [5, 6, 11, 16, 27, 30, 48-51, 53, 59, 63, 68,

	69, 73]<br>- Workflow integration: Is the app fast, easy, comfortable and efficient to use in its user's daily life / workflow / clinical setting (i.e., does it enable the user to perform tasks productively & effectively, without being disturbing)? [1, 4, 7, 13, 27, 30, 32, 37, 43, 48-51, 57, 60, 62, 68, 74]<br>- When there is a problem in the system, can you still perform your job? [50]<br>- Is there a good customer service system (e.g., in-app support service)? [30]<br>- Do you feel confident using the app? [5, 6, 45, 50, 57, 75-77]<br>- Do you intend to use the app/specific functionalities in daily life (e.g., longer than 6 months)? [5, 43, 45, 48, 51, 56, 57, 59, 61, 62, 73, 75, 77, 78]<br>- How often would you use this app (frequency from daily to never)? [14, 16, 29, 36, 37, 48, 50, 55, 58, 62, 77, 79]<br>- How much does the app cost / what is its price (i.e., free or chargeable; if chargeable: cost of downloading the basic version, cost of downloading the upgrade version (if applicable), cost of in-app purchases (if applicable), paid subscription)? [9, 10, 12, 14-19, 21-24, 30, 36, 41, 62, 65, 79]<br>- Would you pay for the app and if yes, how much? [5, 14, 16, 29]<br>- User engagement: What strategies does the app use to encourage continued engagement? E.g., entertainment (e.g., fun, interesting presentation, inspirational quote of the day, videos, audio), gamification (e.g., points, banners), incentivization (e.g., rewards), monetary benefit (e.g., coupons), interactivity (e.g., messages, feedback, chat, real time / asynchronous response), reminders/notifications to use the app. [5, 7, 9, 14, 21, 29, 30, 33, 37, 38, 45, 49, 51, 54, 62, 68, 73, 78, 80-82]<br>- What is your overall experience / impression of the app? [14, 30, 37, 44, 52, 56, 59, 62, 69, 76, 81]<br>- What are your most liked / helpful / best working app part(s) / functions? [2, 37, 45, 46, 53, 55, 57, 72, 83]<br>- What are your least liked / helpful app part(s) / functions / main limitations? [2, 36, 37, 54, 55, 57, 72]<br>- What suggestions for improvements / additional functions / information do you have? [2, 28, 36-38, 45, 53-55, 57, 63, 83-86]<br>- If you decide not to use this app, what will be the possible reasons for it? [85]
Maintenance	- Is the source code publicly available? [8]<br>- Is the app built for sustainability (e.g., integration in existing care services, securing long-term financing, engagement with policy makers) and scale? [2]<br>- Is there an opportunity to continue to gather feedback from users (e.g., patients, caregivers, HCP, clinics) after implementation? [2, 43]<br>- Is there an opportunity to continue to obtain digital analytics (e.g., number of openings, uses)? [43, 86]<br>- Is the app regularly updated / improved in a timely manner for improved security/technology, research, and recommendations? [2, 8, 22, 28, 64, 71]
**E. EVALUATION**	
	- Number of app store ratings [9, 12, 14, 15, 17, 21]<br>- Average rating on app store [9, 10, 12, 14-17, 19-22, 24]<br>- Number and length of written reviews [17]<br>- Perceived impact on awareness: How likely is it that the app will increase awareness, knowledge / understanding, intentions / motivation to change, modify attitudes or self-reflection, encourage help seeking and/or behavior change? [11, 14, 16, 17, 37, 61, 63, 73, 82]<br>- Perceived impact on patient empowerment: How likely is it that the app will improve self-management performance (e.g., facilitation of task fulfillment, increase in quality and quantity of tasks performed, supports good service)? [7,

	16, 50, 73]<br>- Perceived impact on confidence in managing the condition: How likely is it that the app will improve confidence in managing the condition? [58, 73]<br>- Perceived impact on control over the condition / self-management / work: How likely is it that the app will improve control over the condition / self-management / work? [32, 49, 50, 58, 63]<br>- Perceived impact on outcomes: How likely is it that the app will improve outcomes such as quality of health care, quality of life, coping ability or mental health? [28, 30, 50, 53, 62, 63, 86, 87]<br>- Satisfaction / acceptability: Does the app satisfy users and meet their needs and expectations? [1, 4-6, 30, 32, 36, 39, 43, 49-51, 55, 58, 63, 68, 74, 81, 88-90]
Scientific evaluation	- Scientific evaluation: Has the app been evaluated using scientific methods with respect to feasibility, acceptability, usability, efficacy, effectiveness, cost-effectiveness, efficiency, safety and/or implementation?<br>If yes, please list the details of the studies (e.g., design and size) and reference to the publications. [1-3, 6, 8, 13, 14, 18, 20-22, 26, 29, 31, 32, 34, 39, 41, 50, 65-68, 78, 89]<br>- What is the impact factor of the journal in which the studies are published? [21]<br>- Is there any specific clinical scenario or patient subgroup in which the app seems particularly likely to be useful? [13]
**F. REQUIREMENTS & FEATURES**	
	- What is the purpose of the app as claimed by market description (e.g., scope, accuracy (i.e., factual content or opinion), anticipated outcome (e.g., support behavior change; improve physical health), self-management, intended for hybrid use with a clinician)? [3, 8, 10, 12-14, 17-19, 21, 27, 29, 31, 65, 69, 76, 91]<br>- Are the intended goals achievable and measurable? [8, 16, 56, 69]<br>- Does the app include information / content that distracts from its purpose? [10, 91, 92]<br>- What is the app's target population (e.g., HCPs, patients, caregivers/parents, general/lay public, condition / situation, age groups)? [7-14, 16-19, 22, 29, 31, 42, 72]
Evidence-based content	- Is the app's postulated purpose backed by scientific research? [8]<br>- Is the app based on any specific theoretical framework, psychological approach / theory or guidelines (e.g., Cognitive Behavioral Therapy, behavior change theories)? [14, 39, 66, 67, 90]<br>- Are industry standards adhered to, so users are not confused about the meaning of certain standards (e.g., metric units) or conventions? [11, 36]<br>- Is the information provided and/or collected by the app relevant, accurate, up-to-date, comprehensive but concise and supported by solid research (i.e., app refers to current scientific literature or guidelines as appropriate)? [3, 7-14, 17, 19, 20, 23, 24, 26, 28-34, 38, 39, 42, 43, 47, 48, 54, 55, 60, 63-65, 69, 72, 73, 80, 82, 93, 94]<br>- Is the information in the app provided by HCPs / experts? [4, 24, 26, 30, 32, 43, 47, 93]<br>- Are all medical information (incl. calculations & formulas) attributed to an author, their training, a specific date of creation, and a date of last modification? [12, 19, 69, 71]
Functionality	- What are the app's **functionalities** (unspecific criteria)? [3, 16, 18, 25, 79, 80]<br><br>**App use initiation**<br>- Login: Is a user account / login necessary to use the app?<br>If yes, is the creation of a new user account and login delayed to allow the user to use a specific functionality first? [16, 77, 95]

	- Instruction: Is there a user manual / tutorial providing guidance how to use the app? [23, 62, 65, 72, 89, 91, 96]<br>- Help section: Are there helpful suggestions for users to follow when unsure how to proceed? [11, 17, 62, 81, 92]<br>- Contact: Is there a possibility to contact the developer (e.g., contact form)? [12, 42, 71, 79]<br>- Search: Is there a text search field? [23]<br><br>**Behavior change support (list following structure of BCT taxonomy (v1) [97]**<br>**Goals and planning**<br>- Goal setting: Is there a possibility of realistic goal setting (on behavior/outcome) considering willingness to change? [6, 14, 21, 24, 30, 43, 47, 62, 64, 66, 73]<br>- Problem solving: Is there a possibility to plan for barriers? [24, 39]<br>- Action planning: Is there a possibility to make and update an individualized action plan (e.g., using helpful tools, checklists)? [10, 39, 62, 64, 73]<br>- Review goals: Is there regular review or feedback on goals, difference between action and goals, as well as an update and change of the goals? [8, 24, 62, 73, 77]<br><br>**Feedback and monitoring**<br>- Monitoring: Is there a possibility of health tracking (e.g., symptoms, vital signs, medication, treatment, behavior, test results, images / photos, appointments)? Note: these functions need to be customizable and not overwhelming to be perfect (e.g., predefined symptoms to select plus free text field) [11, 13, 14, 16, 17, 20-22, 24, 28-30, 41, 43, 46-48, 50, 58, 60, 62, 64-66, 70, 74, 77, 79, 86, 90, 94, 96]<br>- Interoperability and Data Sharing: Does the app enable connection / communication, sharing of information, data, graphs, photos and/or behavior (e.g., via export, upload, messaging) with other devices / systems (e.g., medical records, weight scale, other apps / wearables, camera) and/or other people (e.g., HCP, caregivers, other patients, peers and/or coach)?<br>Note: these functions need to be customizable (e.g., choosing which data to share with whom and for how long) and not overwhelming to be perfect. [1, 6, 7, 9, 11, 14-16, 19-22, 24, 28-30, 38, 39, 41, 46-48, 50, 51, 58, 62-65, 73, 77, 94, 96]<br>- Feedback (data visualization): Does the app provide feedback based on the users' data, treatment, technique, screening and/or behavior (e.g., via statistics, automatic calculations, analytics, predictions)? [7, 10, 11, 13, 14, 16, 17, 19, 21, 22, 24, 28-30, 36, 41, 42, 60, 64, 65, 70, 91, 94, 96]<br>- Decision making support: Does the app provide guidance based on user-entered information (e.g., warnings based on changing health data, recommend consultation with HCPs, treatment or other actions)? [10, 11, 17, 28, 29, 47, 64, 69, 70, 94-96]<br><br>**Social support**<br>- Social support: Does the app enable a cooperative relationship, communication and/or interactive consultation service between HCPs and patients or among patients and/or caregivers (e.g., chatrooms, forum, teams, coaches) for social learning / facilitation, comparison, cooperation and/or connection to other patients? [16, 17, 21-24, 30, 43, 46, 47, 62-64, 66, 70, 77, 79, 82, 94]<br>- Emotional support: Does the app provide care and affection to help users feel safe, accepted, encouraged, valuable, important and cared about to help them cope with stressful feelings or difficult situations (e.g., via hope board)? [21, 32, 43]

Shaping knowledge / information about consequences

- Educational resources: Does the app provide information divided into short sections with informative headers in a logical sequence using a variety of formats (e.g., text, photo, video) including a summary (e.g., glossaries, textbooks, journals, drug guides, teaching tools, information)? [10, 12, 14, 17, 19, 20, 22, 23, 26, 36, 38, 45, 47, 52, 54, 62, 66, 69, 71, 79, 80, 91, 93, 96]
- Medical information: Does the app provide medical information, e.g., on the target condition, treatment options, medical dictionaries, nutrition, etc.? [19, 29, 42, 46, 47, 52, 69, 93, 94]
- Psychoeducation: Does the app provide psychoeducational references / information? [21]
- Information on tests: Does the app provide information on assessments or health tracking tools (i.e., information on useful tests, how and how often to use them)? [47]
- Behavior Change: Does the app provide information about the consequences of continuing and/or discontinuing specific behaviors? [24]
- Information about health professionals/institutions: Does the app provide helpful addresses / numbers, health news? [42, 48]
- Instruction / demonstration of behavior: Does the app provide instruction and/or demonstration on how to perform the behavior (e.g., video tutorials of using measurement instrument)? [10, 23, 24, 29, 39, 62, 64, 91]
- Quizzes: Does the app provide questions to assess users' status with feedback? [30, 47]

Associations

- Reminders: Does the app provide reminders, timers, notifications, prompts, to-do lists (e.g., on personal targets, appointment, treatment, activity, tests) and/or snooze function?
  Note: these functions need to be customizable and not overwhelming to be perfect (e.g., sight, sound and tactile stimulation) [8, 11, 14, 15, 21-24, 29, 30, 36, 42, 43, 45-48, 62, 64, 73, 86, 94, 96]
- Calendar: Does the app provide appointment management tools and/or calendar? [17, 22, 94]

Repetition and substitution

- Behavioral rehearsal: Does the app provide skills training, tips, advice and/or strategies for effective self-management and health promotion such as, e.g., monitoring, or lifestyle strategies (e.g., common measurement devices, time of measurement, avoidance of environmental/allergens exposure, regular physical activity)? [10, 11, 14, 17, 19, 24, 43, 45, 49, 60, 62, 64-66, 70]
- Habits: Does the app encourage positive habit formation? [21, 24, 38, 62, 90]

Comparison of outcomes

- Credible source: Does the app provide external links / list of services / connection to a telephone line? [9, 17, 19, 47, 63, 69, 81]
- Communicating/Sharing information among the general public and HCPs [42]

Rewards and threats

- Incentivization: Does the app use virtual rewards, dialogue support and/or gaming principles to promote health / behavior change (e.g., points, prizes, leader boards, badges, praise, liking)? [11, 14, 20, 21, 24, 30, 43]

Antecedents

- Restructuring the environment: Does the app instruct and/or support the

	restructuring of the physical or social environment? [24]<br>- Body changes: Does the app support mindfulness/meditation, relaxation and/or gratitude? [14, 21]<br><br>**Identity**<br>- Framing/reframing: Cognitive-behavior therapy, acceptance and commitment therapy, dialectical behavior therapy, physical health exercises [21]<br><br>**Functionalities for flexibility**<br>- Is it possible to add personally relevant information, medication, recipes, tips, strategies, to-do lists and/or notes? If yes, how? [22, 36, 41, 46, 47, 60, 65, 74]<br>- Customization (i.e., the end-users manually take changes to meet their needs): Is it possible for users to adapt certain features / contents of the app to their individual preferences (e.g., sound, content, notifications, wallpaper)? If yes, what aspects? Is there a basic and advanced mode for different users? [11, 16, 23, 24, 29, 30, 32, 36, 46, 48-50, 60, 73, 81, 86, 92]<br>- Personalization (i.e., end-users' data is processed by the company/algorithm to meet an individual's needs): Are features / contents of the app adapted by the app / provider to the users' individual preferences? If yes, what aspects? [3, 4, 7, 14, 24, 25, 30, 41, 43, 47, 48, 50, 60, 62, 65, 70, 77, 78, 95]<br><br>**Usefulness**<br>- Usefulness: Is the app useful for the claimed purpose / self-management? [5, 6, 24, 27, 28, 32, 33, 43, 46, 47, 49, 51, 52, 55, 61, 68, 86, 87]<br>- Usefulness of the specific functionalities: Are the specific functionalities of the app useful for the claimed purpose / self-management? [32, 47, 48, 50, 52, 58, 77]<br>- Perceived usefulness: Would you find the app useful for the claimed purpose / self-management? [5, 9, 26-28, 36, 44, 50, 51, 53, 55, 58-60, 62, 73, 88]<br>- Perceived usefulness of the specific functionalities: Would you find the specific functionalities of the app useful for the claimed purpose / self-management? [4, 36, 37, 43, 50, 57, 58, 74, 88]
Usability	- What are the app's usability characteristics (unspecific criteria)? [6, 7, 22, 24, 32, 33, 36, 39, 53, 60, 65, 78, 82, 86]<br>- Ease of learning: Is it easy to learn and memorize how to use the app (e.g., minimal user memory load)? [11, 14, 29, 41, 48, 49, 54, 55, 68, 71, 81, 84, 89, 92]<br>- Ease of use: Is the app easy to use / handle? [1, 3, 4, 6, 8, 11, 16, 19, 27, 30, 32, 41, 49, 51, 52, 59, 61, 65, 68, 78, 86, 88, 89]<br>- Ease of use of the specific functionalities: Is it easy to do certain tasks, such as finding information, collecting data and/or doing calculations? [11, 26, 29, 30, 36, 43, 46, 48, 54, 59]<br>- How is the user experience of the app (subjective: a person's perceptions of utility, ease of use, and efficiency)? [45, 47]<br>- Perceived ease of learning: Was it easy to learn and memorize how to use the app (e.g., did you need to learn a few things before you could use the app)? [1, 3-5, 14, 26, 28, 43, 45, 50, 51, 58, 62, 74, 75]<br>- Perceived ease of use: Would you find the app easy to use, not mentally / physically demanding to complete the tasks? [4, 5, 26, 31, 36, 43, 45, 50, 56, 58, 62, 73-76, 81]<br>- Perceived ease of use of the specific functionalities: Would you find it easy to do certain tasks, such as finding information, collecting data and/or doing calculations? [4, 5, 26, 29, 32, 37, 43, 50, 74]

	- Content understandability: Is the information easy to read and understand by the target population, are explanations in the users' language, instructions concise, precise and polite, spelling and grammar correct?<br>If applicable: Are there user-defined aliases for language? e.g., for patients, medical terms should be defined and used only to familiarize with the terms, terminology and language should be used concisely and consistently. [1, 3, 4, 10, 11, 27, 29, 30, 36, 39, 43, 44, 48, 50, 52, 54, 55, 58, 60, 62, 65, 68, 71-73, 81, 89, 91-95]<br>- Visualization: Does the app use visual aids / pictures to explain ideas or make content more easily understood (e.g., illustration of portion size) and/or use visual cues (e.g., arrows, boxes, bullets, bold) to draw attention to key points? [10, 16, 38, 62, 81, 91, 95]<br>- Automation: Does the app facilitate automation of tasks, e.g., automatic recording / data retrieving, pre-populated fields, automated customer service? [11, 64, 65, 89]<br><br>- How long does it take to use the app / perform the task the app should be used for? [36, 48, 50, 78, 90]<br>- Organization of the app: Is the structure consistent and clear, the arrangement of the contents / information well and clearly organized, easy to find, not confusing or unnecessarily complex and the various functions well integrated? [4, 26, 28, 36, 43, 45, 46, 49, 50, 52, 55, 58, 68, 71, 73, 75, 76, 81, 84, 90, 92, 93]<br>- Ease of navigation: Is the app easy to navigate, intuitive; are interactions (e.g., taps, swipes, pinches, scrolls) / moving between screens logical and consistent, information easy to find without unnecessary steps? [3, 11, 14, 16, 23, 26, 29, 30, 33, 36, 43, 46, 47, 49, 50, 55, 60, 62, 71, 72, 81, 89, 90, 92, 93, 95]<br>- Perceived ease of navigation: Is the app easy to navigate and find what you need? [5, 37, 43, 45, 57, 58, 73, 84]<br>- Does the app adapt to both horizontal and vertical orientations? [95]<br><br>- Users in control: Can users keep the app under control, i.e., are users the initiators of actions, rather than responders (e.g., no unexpected actions, outcomes, providing tangible tools to help user take action such as menu)? [5, 91, 92]<br>- Does the app provide prompt and informative (i.e., concrete and specific) feedback about the users' actions and the current system status (e.g., showing what part of the app is accessed, a progress bar; showing what can be done in current state and where users can go, providing a message when an action has been completed)? [4, 11, 48, 68, 81, 91, 92, 95]<br>- Consistency and standards: Are words, situations, and actions used consistently? [49, 50, 81, 92]<br>- Is access to the app and its data guaranteed at all times (e.g., does the app work offline) or is internet access required for the app to function? [6, 11, 14, 15, 21, 23-25, 28, 38, 43, 47, 49, 50, 63, 65]<br>- What problems / challenges did you have when using the app (e.g., did you sometimes not know what to do next with the app, problems with language / interface)? [26, 36]<br><br>- Aesthetic design/layout: Is the overall app visually appealing and harmonic, the interface and screen pleasant, the text visually clear and easy to read/hear (e.g., graphic design, overall visual appeal, not overcrowded, color scheme, contrast, stylistic consistency and coherence, font, font size & capitalization; arrangement, clarity and size of buttons/icons/menus/lists etc. on the screen

	appropriate or zoomable if needed)? [3, 4, 7, 8, 10, 11, 13, 14, 16, 23, 25, 26, 28-30, 36-38, 43, 44, 46-48, 50, 52-55, 57-60, 64, 68, 72, 73, 80-82, 89, 91-93, 95]<br>- If there are images, graphs, charts, tables and/or videos: Are they appealing, clear, logical, correct, able to use immediately, of high quality and resolution? They should be consistent in terms of style and types of information being presented, clear titles or captions. [3, 10, 11, 14, 16, 29, 38, 47, 55, 62, 64, 91, 92]<br>- Is the image of the app icon large enough and of high resolution? [3, 23, 91]
Privacy	- What about privacy of the app (unspecific criteria)? [2, 4, 31, 32, 34, 79]<br>- Does the app collect any information that makes the patient identifiable? If yes, what information? [13, 19]<br>- Is patient health / identified / anonymized / aggregate data shared? If yes, with whom (e.g., with health care institutions, 3rd party vendors such as google analytics etc.)? [21]<br>- Does the app require only minimal personal data of end-users? [8]<br>- Does the app have a privacy policy? [8, 9, 11, 12, 19-21, 24, 30, 34, 39, 40, 42, 93]<br>- If yes, does it contain information about what data is being collected or accessed, how and by whom end-user data are used, user controls, confidentiality and privacy protection? [1, 8, 12, 19, 21, 39, 93]<br>- Is it easily accessible, concise, clear and understandable for the target users? [1, 8, 9, 19, 21, 40]<br>- Are users notified of changes to the privacy policy? [40]<br>- Does the app ask permission for push notifications? [24]
Security	- What about security measures of the app (unspecific criteria)? [2, 7, 21, 25, 30-32, 34, 68, 79, 93, 98]<br>- Does the app report security measures / policies in place? If yes, what measures? [21, 71, 93]<br>- Perceived security: Do you think this app has appropriate security methods to protect data? [5, 13, 28, 50, 58, 78]<br>- Does the app include a strong data protection mechanism (e.g., identification / authentication mechanism via password / fingerprint, login required)? [9, 11, 14, 15, 17, 19, 20, 23, 24, 38-40, 43, 50, 71, 72]<br>- Does the app provide secure storage of user data (e.g., on the device or server, country of server location, access to the server, cloud backup, duration of data storage, data encryption / anonymization)? [1, 7, 8, 11, 17, 21, 22, 25, 34, 40, 43, 46, 65]<br>- Does the app provide secure transmission of user data (e.g., data end-to-end-encryption / anonymization)? [1, 8, 11, 17, 34, 40, 43]<br>- Is the app free of malware? [39]
Performance	- What are the performance requirements (unspecific criteria)? [3, 28, 79]<br>- Operability: Does the app cover issues such as ensuring the app downloads and installs, launches, and runs consistently on the target device(s) and operating system(s) / platform(s)? [1, 12, 14, 30, 31, 36, 37, 39, 55, 92]<br>- Do the app functions (e.g., measurements, calculations, responses to entered data) and components (e.g., buttons/menus) work accurately? [5, 8, 9, 13, 14, 16, 28, 29, 32, 39, 41, 50, 65, 72, 74, 81, 99]<br>- Does the app always perform as expected / is it predictable? [19, 49-51, 58, 68, 92]<br>- Have you found any technical issues ('bugs') in the app, or things it can't do? [36, 59, 78, 88]<br>- Error prevention (design interfaces that prevent errors from happening in the first place): Do the interfaces make errors impossible, are there workarounds (e.g., text wrapping), does the app prevent mistakes (e.g., entering letters in a

	numbers field), and is there a low error rate (e.g., crashes)? [4, 11, 28, 43, 50, 92]<br>- Error correction / good error messages: Can the app be recovered easily and quickly if an error (e.g., crash or freeze) occurs (i.e., clear error messages about nature of the error, recover and learn how to fix the problem, proper recovery of the app, e.g., clear messages, no obscure codes)? [4, 11, 36, 43, 49, 50, 81, 92]<br>- Speed: Do the app functions and components (buttons/menus) work and respond quickly? [11, 13, 14, 16, 25, 29, 30, 43, 45, 47, 50, 59, 65, 68, 81, 95]<br>- Does the performance remain the same even with a large number of users? [16, 45]<br>- Does the app use the available resources (e.g., battery) sensibly? [16, 30, 45, 50, 62]<br>- If the app shares an environment and resources with other products, is there a negative impact on the app's or the other products' performance? [45, 50]<br>- If the app shares an environment and resources with other users, is there confusion regarding the users' data? [13]<br>- Does the app lead to overheating of the device? [16]

References

1. Chan S, Torous J, Hinton L, Yellowlees P. Towards a framework for evaluating mobile mental health apps. Telemedicine and e-Health; 2015;21(12):1038-41.
2. Bauer AM, Hodsdon S, Bechtel JM, Fortney JC. Applying the Principles for Digital Development: Case Study of a Smartphone App to Support Collaborative Care for Rural Patients With Posttraumatic Stress Disorder or Bipolar Disorder. J Med Internet Res; 2018;20(6):e10048.
3. Cruz Zapata B, Fernández-Alemán JL, Toval A, Idri A. Reusable Software Usability Specifications for mHealth Applications. J Med Syst; 2018;42(3):45.
4. Jeon E, Park H, Jo S, Kang H, Lee JY. Mobile apps providing tailored nursing interventions for patients with metabolic syndrome. Nursing Informatics 2016: IOS Press; 2016. p. 510-4.
5. Collins R. Nurses' perceived usefulness of secure texting applications for the purpose of patient care. Online Journal of Nursing Informatics; 2019;23(1).
6. Schults J, Cooke M, Richards J, Theodoros J, Andresen E, Paterson R, et al. mHealth Applications for Children and Young People With Persistent Pain: A Scoping Review. Clin Nurs Res; 2019;28(7):779-94.
7. Goetz M, Müller M, Matthies LM, Hansen J, Doster A, Szabo A, et al. Perceptions of patient engagement applications during pregnancy: a qualitative assessment of the patient's perspective. JMIR mHealth and uHealth; 2017;5(5):e7040.
8. Vokinger KN, Nittas V, Witt CM, Fabrikant SI, von Wyl V. Digital health and the COVID-19 epidemic: an assessment framework for apps from an epidemiological and legal perspective. Swiss Med Wkly; 2020;150:w20282.
9. Singh K, Diamantidis CJ, Ramani S, Bhavsar NA, Mara P, Warner J, et al. Patients' and Nephrologists' Evaluation of Patient-Facing Smartphone Apps for CKD. Clinical journal of the American Society of Nephrology : CJASN; 2019;14(4):523-9.
10. Davis DW, Logsdon MC, Vogt K, Rushton J, Myers J, Lauf A, et al. Parent education is changing: a review of smartphone apps. MCN: The American Journal of Maternal/Child Nursing; 2017;42(5):248-56.
11. Anderson K, Burford O, Emmerton L. App chronic disease checklist: protocol to evaluate mobile apps for chronic disease self-management. JMIR Res Protoc; 2016;5(4):e6194.
12. Ben-Mussa A, Paget AM. Popular apps on the medical category targeting patients and the general public in the United Kingdom: Do they conform to the Health On the Net Foundation principles? Health Informatics Journal; 2018;24(3):259-76.
13. Wyatt JC, Thimbleby H, Rastall P, Hoogewerf J, Wooldridge D, Williams J. What makes a good clinical app? Introducing the RCP Health Informatics Unit checklist. Clinical Medicine; 2015;15(6):519.
14. Stoyanov SR, Hides L, Kavanagh DJ, Zelenko O, Tjondronegoro D, Mani M. Mobile app rating scale: a new tool for assessing the quality of health mobile apps. JMIR mHealth and uHealth; 2015;3(1):e3422.
15. Jovičić S, Siodmiak J, Watson ID. Quality evaluation of smartphone applications for laboratory medicine. Clin Chem Lab Med; 2019;57(3):388-97.

16. Kabir MA, Rahman SS, Islam MM, Ahmed S, Laird C. Mobile Apps for Foot Measurement in Pedorthic Practice: Scoping Review. Jmir Mhealth and Uhealth; 2021;9(3).
17. Mobasheri MH, Johnston M, King D, Leff D, Thiruchelvam P, Darzi A. Smartphone breast applications–What's the evidence? The Breast; 2014;23(5):683-9.
18. Lorca-Cabrera J, Martí-Arques R, Albacar-Riobóo N, Raigal-Aran L, Roldan-Merino J, Ferré-Grau C. Mobile Applications for Caregivers of Individuals with Chronic Conditions and/or Diseases: Quantitative Content Analysis. Int J Med Inform; 2021;145:104310.
19. Martinengo L, Stona AC, Tudor Car L, Lee J, Griva K, Car J. Education on Depression in Mental Health Apps: Systematic Assessment of Characteristics and Adherence to Evidence-Based Guidelines. J Med Internet Res; 2022;24(3):e28942.
20. Grau I, Kostov B, Gallego J, Fernández-Luque L, Sisó-Almirall A. Assessment method for mobile health applications in Spanish: The iSYScore index. Semergen; 2016;42(8):575-83.
21. Lagan S, Ramakrishnan A, Lamont E, Ramakrishnan A, Frye M, Torous J. Digital health developments and drawbacks: a review and analysis of top-returned apps for bipolar disorder. Int J Bipolar Disord; 2020;8(1):39.
22. Kaliyadan F, Ashique KT. Use of mobile applications in dermatology. Indian Journal of Dermatology; 2020;65(5):371.
23. Sudol NT, Adams-Piper E, Perry R, Lane F, Chen KT. In search of mobile applications for patients with pelvic floor disorders. Female Pelvic Medicine & Reconstructive Surgery; 2019;25(3):252-6.
24. McKay FH, Wright A, Shill J, Stephens H, Uccellini M. Using health and well-being apps for behavior change: a systematic search and rating of apps. JMIR mHealth and uHealth; 2019;7(7):e11926.
25. Martínez-Pérez B, de la Torre-Díez I, López-Coronado M, Rodrigues JJ, editors. Are mobile health cloud apps better than native? 2015 IEEE international conference on communications (ICC); 2015: IEEE.
26. Ahmed M, Oh A, Vanderlee L, Franco-Arellano B, Schermel A, Lou W, et al. A randomized controlled trial examining consumers' perceptions and opinions on using different versions of a FoodFlip© smartphone application for delivery of nutrition information. Int J Behav Nutr Phys Act; 2020;17(1):22.
27. Olfert MD, Barr ML, Hagedorn RL, Long DM, Haggerty TS, Weimer M, et al. Feasibility of a mhealth approach to nutrition counseling in an Appalachian state. Journal of personalized medicine; 2019;9(4):50.
28. Martínez-Pérez B, de la Torre-Díez I, Candelas-Plasencia S, López-Coronado M. Development and evaluation of tools for measuring the quality of experience (QoE) in mHealth applications. Journal of medical systems; 2013;37(5):1-8.
29. Bentvelsen RG, Holten E, Chavannes NH, Veldkamp KE. eHealth for the prevention of healthcare-associated infections: a scoping review. Journal of Hospital Infection; 2021;113:96-103.

30. Oyebode O, Alqahtani F, Orji R. Using machine learning and thematic analysis methods to evaluate mental health apps based on user reviews. IEEE Access; 2020;8:111141-58.
31. Yasini M, Marchand G. Mobile health applications, in the absence of an authentic regulation, does the usability score correlate with a better medical reliability? MedInfo; 2015;216:127-31.
32. Torous J, Firth J, Huckvale K, Larsen ME, Cosco TD, Carney R, et al. The emerging imperative for a consensus approach toward the rating and clinical recommendation of mental health apps. The Journal of nervous and mental disease; 2018;206(8):662-6.
33. Mansson L, Wiklund M, Öhberg F, Danielsson K, Sandlund M. Co-creation with older adults to improve user-experience of a smartphone self-test application to assess balance function. International journal of environmental research and public health; 2020;17(11):3768.
34. Kwan V, Hagen G, Noel M, Dobson K, Yeates K. Healthcare at your fingertips: the professional ethics of smartphone health-monitoring applications. Ethics & Behavior; 2017;27(8):615-31.
35. EUnetHTA Joint Action. 2, Work Package 8. HTA Core Model® version 3.0. 2016.
36. Jeffrey B, Bagala M, Creighton A, Leavey T, Nicholls S, Wood C, et al. Mobile phone applications and their use in the self-management of Type 2 Diabetes Mellitus: a qualitative study among app users and non-app users. Diabetol Metab Syndr; 2019;11:84.
37. Dubov A, Fraenkel L, Goldstein Z, Arroyo H, McKellar D, Shoptaw S. Development of a Smartphone App to Predict and Improve the Rates of Suicidal Ideation Among Transgender Persons (TransLife): Qualitative Study. J Med Internet Res; 2021;23(3):e24023.
38. Songtaweesin WN, LeGrand S, Bandara S, Piccone C, Wongharn P, Moonwong J, et al. Adaptation of a Theory-Based Social Networking and Gamified App-Based Intervention to Improve Pre-Exposure Prophylaxis Adherence Among Young Men Who Have Sex With Men in Bangkok, Thailand: Qualitative Study. J Med Internet Res; 2021;23(11):e23852.
39. Boulos MNK, Brewer AC, Karimkhani C, Buller DB, Dellavalle RP. Mobile medical and health apps: state of the art, concerns, regulatory control and certification. Online journal of public health informatics; 2014;5(3):229.
40. Zapata BC, Niñirola AH, Fernández-Alemán JL, Toval A, editors. Assessing the privacy policies in mobile personal health records. 2014 36th Annual International Conference of the IEEE Engineering in Medicine and Biology Society; 2014: IEEE.
41. Vasiloglou MF, Christodoulidis S, Reber E, Stathopoulou T, Lu Y, Stanga Z, et al. Perspectives and Preferences of Adult Smartphone Users Regarding Nutrition and Diet Apps: Web-Based Survey Study. JMIR Mhealth Uhealth; 2021;9(7):e27885.
42. Saeedi MG, Kalhori SRN, Nouria R, Yasini M, Nouri R, Yasini M, editors. Persian mHealth apps: A cross sectional study based on use case classification. MIE; 2016.

43. Al Ayubi SU, Parmanto B, Branch R, Ding D. A persuasive and social mHealth application for physical activity: a usability and feasibility study. JMIR mHealth and uHealth; 2014;2(2):e2902.
44. Hsieh KL, Fanning JT, Rogers WA, Wood TA, Sosnoff JJ. A Fall Risk mHealth App for Older Adults: Development and Usability Study. JMIR Aging; 2018;1(2):e11569.
45. Caballero P, Ortiz G, Garcia-de-Prado A, Boubeta-Puig J. Paving the way to collaborative context-aware mobile applications: a case study on preventing worsening of allergy symptoms. Multimedia Tools and Applications; 2021;80(14):21101-33.
46. Jiam N, Hoon Jr AH, Hostetter C, Khare M. IIAM (important information about me): a patient portability profile app for adults, children and families with neurodevelopmental disabilities. Disability and Rehabilitation: Assistive Technology; 2017;12(6):599-604.
47. O'Reilly SL, Laws R. Health ‐ e mums: Evaluating a smartphone app design for diabetes prevention in women with previous gestational diabetes. Nutrition & dietetics; 2019;76(5):507-14.
48. Eisner E, Drake RJ, Berry N, Barrowclough C, Emsley R, Machin M, et al. Development and Long-Term Acceptability of ExPRESS, a Mobile Phone App to Monitor Basic Symptoms and Early Signs of Psychosis Relapse. JMIR Mhealth Uhealth; 2019;7(3):e11568.
49. Gao M, Kortum P, Oswald F, editors. Psychometric evaluation of the use (usefulness, satisfaction, and ease of use) questionnaire for reliability and validity. Proceedings of the human factors and ergonomics society annual meeting; 2018: SAGE Publications Sage CA: Los Angeles, CA.
50. Maramba I, Chatterjee A, Newman C. Methods of usability testing in the development of eHealth applications: A scoping review. Int J Med Inform; 2019;126:95-104.
51. Naslund JA, Aschbrenner KA, Bartels SJ. Wearable devices and smartphones for activity tracking among people with serious mental illness. Mental health and physical activity; 2016;10:10-7.
52. Jain YS, Garg A, Jhamb DK, Jain P, Karar A. Preparing India to Leverage Power of Mobile Technology: Development of a Bilingual Mobile Health Tool for Heart Patients. Cardiovasc Hematol Agents Med Chem; 2019;17(2):125-34.
53. Signorelli GR, Monteiro-Guerra F, Rivera-Romero O, Nunez-Benjumea FJ, Fernandez-Luque L. Breast Cancer Physical Activity Mobile Intervention: Early Findings From a User Experience and Acceptability Mixed Methods Study. JMIR Form Res; 2022;6(6).
54. Groen G, Jörns-Presentati A, Dessauvagie A, Seedat S, van den Heuvel LL, Suliman S, et al. Development of a Mobile Application for Detection of Adolescent Mental Health Problems and Feasibility Assessment with Primary Health Care Workers. Issues in Mental Health Nursing; 2022;43(11):1046-55.
55. Le Marne FA, Butler S, Beavis E, Gill D, Bye AME. EpApp: Development and evaluation of a smartphone/tablet app for adolescents with epilepsy. J Clin Neurosci; 2018;50:214-20.

56. Sanatkar S, Counson I, Mackinnon A, Bartholomew A, Glozier N, Harvey S. Preliminary Investigation of Shift, a Novel Smartphone App to Support Junior Doctors' Mental Health and Well-being: Examination of Symptom Progression, Usability, and Acceptability After 1 Month of Use. J Med Internet Res; 2022;24(9):e38497.
57. Bosse JD, Hoffman K, Wiest K, Todd Korthuis P, Petluri R, Pertl K, et al. Patient evaluation of a smartphone application for telehealth care of opioid use disorder. Addict Sci Clin Pract; 2022;17(1):50.
58. Hampton P, Richardson D, Brown S, Goodhead C, Montague K, Olivier P. Usability testing of MySkinSelfie: a mobile phone application for skin self-monitoring. Clin Exp Dermatol; 2020;45(1):73-8.
59. Schmidt T, Mewes P, Hoffmann JD, Müller-von Aschwege F, Glitza JI, Schmitto JD, et al. Improved aftercare in LVAD patients: Development and feasibility of a smartphone application as a first step for telemonitoring. Artif Organs; 2020;44(3):248-56.
60. Nolen SL, Giblin-Scanlon LJ, Boyd LD, Rainchuso L. Development and Testing of a Smartphone Application Prototype for Oral Health Promotion. J Dent Hyg; 2018;92(2):6-14.
61. Werner-Seidler A, Wong Q, Johnston L, O'Dea B, Torok M, Christensen H. Pilot evaluation of the Sleep Ninja: a smartphone application for adolescent insomnia symptoms. BMJ Open; 2019;9(5):e026502.
62. Egan KJ, Hodgson W, Imperatore G, Dunlop MD, Maguire R, Kirk A. Supporting Physical Activity for Informal Caregivers during and beyond COVID-19: Exploring the Feasibility, Usability and Acceptability of a Digital Health Smartphone Application, 'CareFit'. Int J Environ Res Public Health; 2022;19(19).
63. Lao SSW, Chair SY. The feasibility of smartphone-based application on cardiac rehabilitation for Chinese patients with percutaneous coronary intervention in Macau: a qualitative evaluation. Int J Qual Stud Health Well-being; 2022;17(1):2023940.
64. Guan Z, Sun L, Xiao Q, Wang Y. Constructing an assessment framework for the quality of asthma smartphone applications. BMC Med Inform Decis Mak; 2019;19(1):192.
65. Vasiloglou MF, Christodoulidis S, Reber E, Stathopoulou T, Lu Y, Stanga Z, et al. What healthcare professionals think of “nutrition & diet” apps: an international survey. Nutrients; 2020;12(8):2214.
66. Lalloo C, Shah U, Birnie KA, Davies-Chalmers C, Rivera J, Stinson J, et al. Commercially available smartphone apps to support postoperative pain self-management: scoping review. JMIR mHealth and uHealth; 2017;5(10):e8230.
67. Bennion MR, Hardy GE, Moore RK, Kellett S, Millings A. e-Therapies in England for stress, anxiety or depression: how are apps developed? A survey of NHS e-therapy developers. BMJ Health Care Inform; 2019;26(1).
68. Laugwitz B, Held T, Schrepp M, editors. Construction and evaluation of a user experience questionnaire. Symposium of the Austrian HCI and usability engineering group; 2008: Springer.

69. Charnock D, Shepperd S, Needham G, Gann R. DISCERN: an instrument for judging the quality of written consumer health information on treatment choices. Journal of Epidemiology & Community Health; 1999;53(2):105-11.
70. Gorini A, Mazzocco K, Triberti S, Sebri V, Savioni L, Pravettoni G. A P5 Approach to m-Health: design suggestions for advanced mobile health technology. Frontiers in psychology; 2018;9:2066.
71. Karsalia M, Malik R. Evaluation of free mobile health applications for pelvic organ prolapse and urinary incontinence. Am J Surg; 2022;223(1):187-93.
72. East-Richard C, Laplante L, Vezina J, Cellard C. Psychological evaluation through the mobile applications. CANADIAN PSYCHOLOGY-PSYCHOLOGIE CANADIENNE; 2018;59(3):262-71.
73. Yong TSM, Perialathan K, Ahmad M, Juatan N, Majid LA, Johari MZ. Perceptions and Acceptability of a Smartphone App Intervention (ChildSafe) in Malaysia: Qualitative Exploratory Study. Jmir Pediatrics and Parenting; 2021;4(2).
74. Ambrosini GL, Hurworth M, Giglia R, Trapp G, Strauss P. Feasibility of a commercial smartphone application for dietary assessment in epidemiological research and comparison with 24-h dietary recalls. Nutr J; 2018;17(1):5.
75. Brooke J. SUS-A quick and dirty usability scale. Usability evaluation in industry; 1996;189(194):4-7.
76. Armenta V, Warrell L, Nazneen N, Escobedo L, Arriaga RI, editors. ActEarly: a bi-national evaluation study of a mobile application for tracking developmental milestones. Proceedings of the IX Latin American Conference on Human Computer Interaction; 2019.
77. Hartzler AL, Venkatakrishnan A, Mohan S, Silva M, Lozano P, Ralston JD, et al., editors. Acceptability of a team-based mobile health (mHealth) application for lifestyle self-management in individuals with chronic illnesses. 2016 38th Annual International Conference of the IEEE Engineering in Medicine and Biology Society (EMBC); 2016: IEEE.
78. Scott AR, Alore EA, Naik AD, Berger DH, Suliburk JW. Mixed-methods analysis of factors impacting use of a postoperative mHealth app. JMIR mHealth and uHealth; 2017;5(2):e6728.
79. Biviji R, Williams KS, Vest JR, Dixon BE, Cullen T, Harle CA. Consumer Perspectives on Maternal and Infant Health Apps: Qualitative Content Analysis. J Med Internet Res; 2021;23(9):e27403.
80. Fijacko N, Creber RM, Gosak L, Kocbek P, Cilar L, Creber P, et al. A Review of Mortality Risk Prediction Models in Smartphone Applications. Journal of Medical Systems; 2021;45(12).
81. Slaughter L, Harper B, Norman K, editors. Assessing the equivalence of the paper and on-line formats of the QUIS 5.5. Proceedings of Mid Atlantic Human Factors Conference; 1994.
82. Fuller-Tyszkiewicz M, Richardson B, Klein B, Skouteris H, Christensen H, Austin D, et al. A Mobile App-Based Intervention for Depression: End-User and Expert Usability Testing Study. JMIR Ment Health; 2018;5(3):e54.

83. Alnasser A, Kyle J, Alkhalifah A, Marais D. Relationship Between Evidence Requirements, User Expectations, and Actual Experiences: Usability Evaluation of the Twazon Arabic Weight Loss App. JMIR Hum Factors; 2018;5(2):e16.
84. Gomes N, Caroço J, Rijo R, Martinho R, Querido A, Peralta T, et al. Evaluation of an e-health platform for informal caregivers and health professionals: the case study of Help2Care. Inform Health Soc Care; 2022;47(2):144-58.
85. Soomro N, Chhaya M, Soomro M, Asif N, Saurman E, Lyle D, et al. Design, Development, and Evaluation of an Injury Surveillance App for Cricket: Protocol and Qualitative Study. JMIR Mhealth Uhealth; 2019;7(1):e10978.
86. Birkhoff S. Examining the Usability and Acceptability of a Patient-Centered Mobile Health Tracking App for Patients Undergoing Radiation Therapy for Cancer Treatment: Villanova University; 2017.
87. Bining M, Wasserman S, Brahim LO, Belzile E, Magalhaes M, Lambert SD. An Evaluation of Publicly Available Smartphone Apps to Support Unpaid Cancer Caregivers. J Pain Symptom Manage; 2022;63(3):430-9.
88. Forman EM, Goldstein SP, Zhang F, Evans BC, Manasse SM, Butryn ML, et al. OnTrack: development and feasibility of a smartphone app designed to predict and prevent dietary lapses. Transl Behav Med; 2019;9(2):236-45.
89. Zapata BC, Fernández-Alemán JL, Idri A, Toval A. Empirical studies on usability of mHealth apps: a systematic literature review. Journal of medical systems; 2015;39(2):1-19.
90. Gartenberg D, Thornton R, Masood M, Pfannenstiel D, Taylor D, Parasuraman R. Collecting health-related data on the smart phone: mental models, cost of collection, and perceived benefit of feedback. Personal and ubiquitous computing; 2013;17(3):561-70.
91. Shoemaker SJ, Wolf MS, Brach C. Development of the Patient Education Materials Assessment Tool (PEMAT): a new measure of understandability and actionability for print and audiovisual patient information. Patient education and counseling; 2014;96(3):395-403.
92. Nielsen J. Ten usability heuristics. http://www. nngroup. com/articles/ten-usability-heuristics/(acc-essed …; 2005.
93. Jin M, Kim J. Development and evaluation of an evaluation tool for healthcare smartphone applications. Telemedicine and e-Health; 2015;21(10):831-7.
94. Dunn Lopez K, Chae S, Michele G, Fraczkowski D, Habibi P, Chattopadhyay D, et al. Improved readability and functions needed for mHealth apps targeting patients with heart failure: An app store review. Research in Nursing & Health; 2021;44(1):71-80.
95. Cruz Zapata B, Hernandez Ninirola A, Idri A, Fernández-Alemán JL, Toval A. Mobile PHRs compliance with Android and iOS usability guidelines. Journal of medical systems; 2014;38(8):1-16.
96. Choi YK, Demiris G, Lin SY, Iribarren SJ, Landis CA, Thompson HJ, et al. Smartphone Applications to Support Sleep Self-Management: Review and Evaluation. J Clin Sleep Med; 2018;14(10):1783-90.
97. Michie S, Richardson M, Johnston M, Abraham C, Francis J, Hardeman W, et al. The behavior change technique taxonomy (v1) of 93 hierarchically clustered

techniques: building an international consensus for the reporting of behavior change interventions. Annals of behavioral medicine; 2013;46(1):81-95.
98. Bining M, Wasserman S, Brahim LO, Belzile E, Magalhaes M, Lambert SD. An Evaluation of Publicly Available Smartphone Apps to Support Unpaid Cancer Caregivers. Journal of Pain & Symptom Management; 2022;63(3):430-9.
99. Sarzynski E, Decker B, Thul A, Weismantel D, Melaragni R, Cholakis E, et al. Beta testing a novel smartphone application to improve medication adherence. Telemedicine and e-Health; 2017;23(4):339-48.
